# Effects of NaCl Concentration on the Behavior of *Vibrio brasiliensis* and Transcriptome Analysis

**DOI:** 10.3390/foods11060840

**Published:** 2022-03-15

**Authors:** Shuyang Hu, Yuwei Li, Boran Wang, Lijun Yin, Xin Jia

**Affiliations:** College of Food Science and Nutritional Engineering, China Agricultural University, Beijing 100083, China; hushuyangyllh@163.com (S.H.); liyuwei9@126.com (Y.L.); wang_remon@163.com (B.W.); ljyin@cau.edu.cn (L.Y.)

**Keywords:** *Vibrio*, outer membrane proteins, transcriptome analysis, osmotic stress, stress response

## Abstract

The growth of *Vibrio* bacteria is affected by environmental conditions, and unfavorable conditions will produce different degrees of stress on *Vibrio*. The cells respond to the stress on the bacteria through changes in biological characteristics and transcriptomes. To study the effect of NaCl concentration on *Vibrio brasiliensis*, we have determined the biological characteristics of the 0%, 1%, 2%, 3%, 5%, and 7% NaCl concentrations cultured *V. brasiliensis* to research the salt stress to bacteria. We found that the biological properties of *V. brasiliensis* cultured with different NaCl concentrations were different, and the expression of outer membrane proteins of *V. brasiliensis* changed when it was grown under different NaCl concentrations. When bacteria cultured in higher NaCl concentrations (3%, 5% and 7% NaCl), the sodium-type flagellar protein MotY was found. Finally, the transcriptome analysis of *V. brasiliensis* cultured with 0% NaCl and 7% NaCl was carried out to find out the differentially expressed genes. We found that the same gene have opposite up-regulated and down-regulated expression in two treatments, indicating that these types of genes are regulated different in low and high osmotic stress.

## 1. Introduction

Increasing seafood consumption around the world has attracted more attention to the control of bacterial infections. *Vibrio* species are Gram-negative bacteria which are facultative anaerobic and widely distributed in seawater and ocean animals [1]. In recent years, *V.* *parahaemolyticus*, *V. vulnificus*, and *V. cholerae*, have been reported to cause human intestinal infections, acute diarrhea and even shock death [2]. In recent years, the case of foodborne illnesses and wound infections caused by pathogenic *Vibrio* species have occurred. In the summer of 2015, there was an outbreak of *V. parahaemolyticus* infection caused by the consumption of raw oysters in British Columbia, Canada [3]. In August 2016, a cholera outbreak in South Korea resulted in acute watery diarrhea in three patients [4]. In 2018, an outbreak of cholera in the northern part of Algeria resulted in more than 200 patients with watery diarrhea symptoms and two deaths [5]. In many countries, pathogenic *Vibrio* species are a primary cause of bacterial diarrhea, possibly due to consumption of seafood or water contaminated with *Vibrio* pathogens. Among the pathogenic *Vibrio* species, the more harmful and pathogenic bacteria are *V. parahaemolyticus*, *V. vulnificus*, and *V. cholerae*. Clinical studies have found that there are three main diseases that occur after human infection with *V. parahaemolyticus*, that is acute gastroenteritis. *V. vulnificus* is an important opportunistic pathogen that can cause human wound infections and gastrointestinal disease, through exposure skin to seawater or consumption of seafood [6]. *V. cholerae* is the bacterial pathogen that causes cholera disease, devilling many developing countries and areas of poor environmental health, and causing approximately 95,000 deaths each year [7]. The growth and propagation of *Vibrio* need suitable environmental conditions. Although *Vibrio* most commonly occurred in large numbers in seafood, such as shellfish and fish [6], it has also been found in freshwater environment [8,9]. Their strong adaptability makes it difficult to control the Vibrio in many aquatic products, since treatments used in food processing and preservation often utilize stress factors for which *Vibrio* is resistant [10]. For instance, increasing salinity is one of the most commonly employed factors for food conservation and aquatic product processing due to the fact that NaCl does not only satisfy people’s tastes in aquatic products processing, but the high content of NaCl in food can potentially extend storage time by reducing water activity. Nonetheless, *V. parahaemolyticus* was isolated from pickled food containing high quantities of salt [11]. The ability to survive and breed in severe conditions is a prerequisite for food-borne bacteria.

Needless to say, microorganisms must be able to adapt to environmental changes or stresses. Outer membrane proteins (OMPs) located at the outermost area of the bacterial cells are likely to play a critical role in osmoregulation to adapt to environmental stress. The outer membrane structure and composition of Gram-negative bacteria are complex, so when extracting bacterial outer membrane proteins, multi-step processing is required to obtain relatively pure outer membrane proteins. At present, there are three main methods for the extraction of bacterial outer membrane proteins, namely Sodium dodecyl sulfate (SDS), Sarkosyl and PMSF methods [12]. Among them, Sarkosyl method is popular due to the variety of outer membrane proteins extracted. Sarkosyl (sodium lauryl sarcosinate) is a detergent that prepares the outer membrane by differential solubilization of the inner membrane [13]. In addition, liquid chromatography-tandem mass spectrometry (LC-MS/MS) provides a great deal of information about proteins and peptides in biological samples. Furthermore, LC-MS/MS can detect a relatively high number of peptides and amino acid sequences, so that the protein identification has a high confidence. For the past few years, LC-MS/MS methods were utilized in analysis and identification of bacterial OMPs. The possible roles of major OMPs of *V. angullarum* are shown to stimulate biofilm formation, which allows *Vibrio* to survive stressful environments [14]. The altered NaCl concentration of the culturing medium of *V. alginolyticus* [15] and *V. parahaemolyticus* [16] result in the change in the expression pattern of OMPs.

Bacteria may alter protein expression and metabolism to adaptation to the changing environment when they infect hosts or in a floating state. Additionally, *rsb*-encoding a signaling-hub stressosome has also been reported to respond to environmental stress through altered phosphorylated levels in *Vibrio* genera. Previous studies have shown that the stressosome phosphorylation level of the Gram-negative bacterium *V.* *brasiliensis* is regulated by environmental stress [17]. However, within Gram-negative bacteria, stressosome downstream proteins and activation of target genes have yet to know. Many studies have demonstrated that environmental stress has been manifested as the expression of virulence genes in some *Vibrio* that have implications on the competitiveness, stress tolerance, and the ability of pathogenic *Vibrio* to cause infection. For example, the high salt adaptability of *V.* *parahaemolyticus* strains significantly improves survival under environmental stresses [18]. Many studies have indicated that these conditions have a significant influence on the virulence and growth of pathogenic *Vibrio* species [19].

However, the survival strategies of *Vibrio* bacteria to withstand different kinds of environmental stress in the process of aquatic products processing are not well-understood. Most aquatic products need to be added with NaCl for further processing, but the *Vibrio* infected in aquatic products can survive by responses the NaCl stress. Although some studies on *Vibrio* bacteria such as *V. parahaemolyticus* and *V. alginolyticus* at different NaCl concentrations have been published [20,21], *Vibrio* how to respond to this environmental stress and related signal transduction pathways and which key genes expression changes are not yet clear.

Through the influence of different NaCl concentrations in the environment on the biological characteristics and gene expression of *V. brasiliensis*, we found that *V. brasiliensis* was affected differently under various NaCl concentrations. Transcriptome analysis further revealed that the bacteria are exposed to NaCl stress, eventually adapt to the new environment in response to stress by changing gene expression. In this study, *V. brasiliensis* has been used as a model strain to study the behavioral, morphometric and gene expression effect in response to NaCl stress. The morphology, viability, growth rate, swimming ability, outer membrane protein expression levels and transcriptomic analyses have been examined for *V. brasiliensis* cultivated in various NaCl conditions. We believe this study could provide mechanistic details as to how the *Vibrio* response to NaCl stress.

## 2. Materials and Methods

### 2.1. Bacterial Strains and Culture Conditions

*Vibrio brasiliensis* (LMG 20546) strain was purchased from Marine Culture Collection of China (MCCC). Cultures of *V. brasiliensis* were separately grown at 28 °C in 200 mL sterilized Marine Broth (Coolaber Science & Technology Co., Ltd., Beijing, China) medium supplemented with 0, 1%, 3%, 5% and 7% NaCl or on Marine Broth agar plates containing the same NaCl gradient. Liquid medium or solid plate containing 2% NaCl was use as a positive control of the salt stress experiment.

### 2.2. Growth Conditions of V. brasiliensis in Salt Stress

To study the tolerance of NaCl stress on the growth of *V. brasiliensis*, bacteria strain was cultured overnight in Marine Broth medium. The overnight cultures were diluted at 1:100 and then cultivated at 28 °C at 150 r/min in triplicate in Marine Broth medium containing various NaCl concentrations (0, 1%, 2%, 3%, 5%, and 7%) for 24 h. The bacterial growth rate was measured spectrophometrically (OD_600_) once every two hours. The bacterial liquids cultivated with 0%, 1%, 2%, 3%, 5%, and 7% NaCl were adjusted to an OD_600_ value of 0.8, and 100 μL of bacterial liquids were drawn to the liquid medium of the corresponding NaCl concentration and cultured for 18 h at 28 °C, and then the number of viable bacteria was calculated by the dilution coating method as the bacterial survival ability. Triplicate experiments and data were expressed as the mean ± standard deviation (SD).

### 2.3. Morphological Analysis of V. brasiliensis in Salt Stress

Morphological changes in liquid medium with different NaCl cultivated cells were monitored using a scanning electronic microscope (SEM) as previously described [22]. In brief, *V. brasiliensis* was cultured in 500 mL of Marine Broth 2216 at 28 °C for 150 r/min until OD_600_ reach 0.8. The *V. brasiliensis* were collected by centrifugation at 7645× *g* for 15 min at 4 °C. Cultural pellets were washed with phosphate buffer solution (PBS). Then, discarded the supernatant and added 2.5% glutaraldehyde (*v*/*v*) fixative solution, the mixture was stored at 4 °C overnight. Took appropriate number of cultural pellets were suspended and rinsed in 0.1 M (pH 7.0) PBS. Following, 1% osmic acid solution was used to immobilized samples with 1–2 h to fix cells on glass slides. The fixed cells were rinsed with 0.1 M PBS, dehydrated by ethanol treatment, and then, the samples so treated were mixed with a mixture of ethanol and isoamyl acetate (*v*/*v* = 1:1) for 30 min and dried. Bacterial culture samples were then coated and examined in a scanning electron microscope (SU8010, HITACHI, Tokyo, Japan) at an accelerating voltage of 3.0 kV.

### 2.4. Swimming Motility of V. brasiliensis in Salt Stress

The determination of the swimming ability of the bacteria was based on a previously published method with minor modification on OD_600_ value and the inoculation amount of bacterial solution [23,24]. Briefly, *V. brasiliensis* grown in MB medium with 0, 1%, 3%, 5%, and 7% NaCl concentration was cultivated to an OD_600_ of 0.6, and then 1.5 μL of the bacterial suspension was spread onto the Marine Broth plates containing 0.3% agar for the swimming test. The diameter of the halo resulting from bacteria swimming motion was measured after 18 h inoculation. Swimming motility were calculated from triplicate measurements and data were expressed as the mean ± standard deviation (SD).

### 2.5. Extraction and Analysis of Outer Membrane Proteins (OMPs)

#### 2.5.1. OMPs Extraction

The outer membrane protein extraction according to previous method [25,26]. The *V. brasiliensis* cells were harvested by centrifugation at 7645× *g* for 15 min at 4 °C. Cell pellets were washed three times with 20 mmol/L Tris-HCl buffer (pH 8.0), before re-suspension in 20 mL Tris-HCl (pH 8.0). Cells were lysed by sonication (JY92-IIN, Ningbo Xinyi ultrasonic equipment Co., Ltd., Ningbo, China) at an output power of 200 W (pulse on 3 s, pulse off 3 s) until the bacteria liquid was clear and transparent. Cell debris was removed by centrifugation at 7645× *g* for 10 min at 4 °C. The supernatant containing crude membrane fraction was centrifuged at 57,500× *g* for 60 min at 4 °C. The resulting pellet was suspended in 3 mL 20 mmol/L Tris-HCl (pH 8.0) containing 2 mmol/L EDTA and 0.5% Sarkosyl solution to solubilize OMPs, and the mixture incubated at room temperature for 30 min. OMPs were recovered by centrifugation at 71,000× *g* for 30 min at 4 °C; the resulting pellet was re-suspended in 10 mmol/L PBS (pH 7.4). Protein concentration was determined based on Bradford method with bovine serum albumin as standard.

#### 2.5.2. Sodium Dodecyl Sulfate—Polyacrylamide Gel Electrophoresis (SDS-PAGE)

The isolated OMPs as a result of salt stress was analyzed using a DYY-6C PowerStation (Beijing Liuyi Biological Technology Co., Ltd., Beijng, China) on 8–20% acrylamide precast gels (15 × 10 cm). Protein extraction were mixed in a 3:1 ratio with 4× loading buffer (10 μL) containing β-Mercaptoethanol. The mixture was denatured at 100 °C for 5 min before centrifugation at 10,000× *g* for 2 min using a benchtop centrifugation (Changsha Pingfan Instrument Co., Ltd., Changsha, China). Samples of 30 μL were loaded on the gel; the gels were stained with Coomassie brilliant blue R-250 dissolved in distaining solution. The resulting gels were scanned on a Clinx Geno Sens Capture system (Clinx Science Instruments Co., Ltd., Shanghai, China).

#### 2.5.3. In Gel Tryptic Digest for Mass Spectrometry Analysis

SDS-PAGE gel containing protein-of-interest was diced into 1 mm^3^ pieces, the individual gel pieces were placed into 0.65 mL siliconized tubes (Thermo Scientific, Waltham, MA, USA). Following, disulfide bond within proteins were reduced with 25 mM dithiothreitol (DTT) and alkylated with 55 mM iodoacetamide. In-gel digestion was performed using sequencing grade-modified trypsin in 50 mM ammonium bicarbonate at 37 °C overnight. The peptides were extracted twice with 1% trifluoroacetic acid in 50% acetonitrile aqueous solution for 30 min. The peptide extracts were then centrifuged in a SpeedVac to reduce the volume.

For LC-MS/MS analysis, digested peptides were separated by a 60 min gradient elution at a flow rate 0.300 μL/min on a Thermo-Dionex Ultimate 3000 HPLC system, which was directly interfaced with the Thermo Orbitrap Fusion mass spectrometer. The analytical column was on an analytical reverse phase fused silica capillary column (75 μm ID, 150 mm length; Upchurch, Oak Harbor, WA, USA) packed with C-18 resin (300 A, 5 μm; Varian, Lexington, MA, USA). Mobile phase A consisted of 0.1% formic acid, and mobile phase B consisted of 100% acetonitrile and 0.1% formic acid. The Orbitrap Fusion mass spectrometer was operated in the data-dependent acquisition mode using Xcalibur 3.0 software, with a single full-scan mass spectrum in the Orbitrap (350–1550 *m*/*z*, 120,000 resolution) followed by 3 s data-dependent MS/MS scans in an Ion Routing Multipole at 30% normalized collision energy (HCD). The MS/MS spectra from each LC-MS/MS run were searched against the selected database using Proteome Discovery searching algorithm (version 1.4).

### 2.6. Transcriptome Analysis

Transcriptome analysis was performed on *V. brasiliensis* cultured in 0, 2%, and 7% NaCl. Three biological replicates were prepared for the bacteria cultured in each group. Total RNA was extracted from the bacteria using TRIzol^®^ Reagent (Invitrogen, Carlsbad, CA, USA) according to the manufacturer’s instructions, and then the quality of RNA was determined by 2100 Bioanalyser (Agilent, Palo Alto, CA, USA) and Nanodrop 2000 (NanoDrop, Waltham, MA, USA) was used to quantify the RNA. Only high-quality RNA sample (OD260/280 = 1.8~2.0, RIN ≥ 6.5) was used to construct the library. RNA-seq transcriptome library was constructed using TruSeq^TM^ RNA sample preparation Kit from Illumina (San Diego, CA, USA). Libraries were size selected for cDNA target fragments of 200 bp on 2% Low Range Ultra Agarose followed by PCR amplified using Phusion DNA polymerase (NEB, Ipswich, MA, USA) for 15 PCR cycles. After quantified by TBS380, paired-end RNA-seq sequencing library was sequenced with the Illumina HiSeq × TEN (2 × 150 bp read length). The processing of original images to sequences, base-calling, and quality value calculations were performed using the Illumina GA Pipeline (version 1.6), in which 150 bp paired-end reads were obtained. A Perl program was written to select clean reads by removing low-quality sequences, reads with more than 5% of N bases (unknown bases) and reads containing adaptor sequences. The data generated from Illumina platform were used for bioinformaticis analysis. All of the analyses were performed using the online platform of Majorbio Cloud Platform (www.majorbio.com, data has been deposited on 6 January 2022) from Shanghai Majorbio Bio-pharm Technology Co., Ltd. (Shanghai, China). High quality reads in each sample were mapped to the reference genome by using the Bowtie2 (http://bowtie-bio.sourceforge.net/bowtie2/index.shtml, data has been deposited on 6 January 2022) analysis tool. Then the rRNA contamination rate was assessed by randomly selecting 10,000 raw reads in each sample are aligned to Rfam database (http://rfam.xfam.org/, data has been deposited on 6 January 2022) with blast method. Based on the annotation results, percentage of rRNA in each sample is calculated, which is estimated as rRNA contamination. After that, gene expression analysis is performed by using analysis tools. Quantity gene and isoform abundances form single-end or paired-end RNA-Seq data using RSEM (http://deweylab.github.io/RSEM/, data has been deposited on 6 January 2022). FPKM and TPM method is used to calculated expression level, FPKM represents fragments per kilpbase of exon model per million mapped reads, TPM represents transcript per million mapped reads. The correlation analysis between samples and principal component analysis (PCA) were analysed using the RSEM software (http://deweylab.github.io/RSEM/, data has been deposited on 6 January 2022) on the online platform of Majorbio Cloud Platform (www.majorbio.com, data has been deposited on 6 January 2022), and TPM was used to an expression indicator. Furthermore, the Pearson Correlation algorithm, the Complete-linkage clustering method and the Euclidean Distance algorithm were used to analyse the correlation between samples. For each data set, and for each alignment and quantification protocol, we identified differentially expressed genes by using the DESeq2 (http://bioconductor.org/packages/release/bioc/html/DESeq2.html, data has been deposited on 6 January 2022) packages [27,28], with a *p* < 0.05 and abs(log_2_ Fold Change) ≥ 1 as the threshold of differential expression [29].

## 3. Results and Discussion

### 3.1. Effect of Salt Stress on Growth and Survival of V. brasiliensis

**Figure 1:**
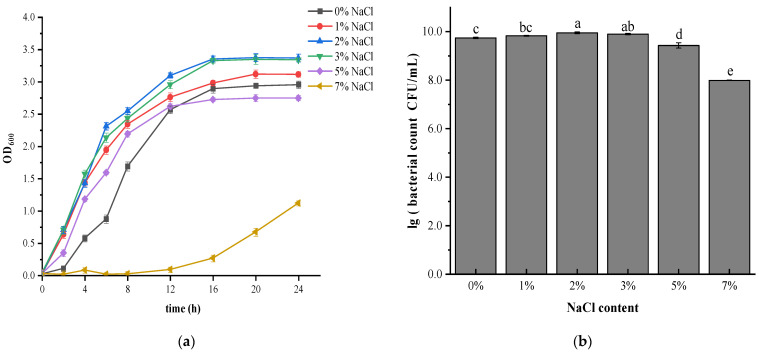
Effect of salt concentration on growth of *V. brasiliensis*. (**a**) Growth rate of *V. brasiliensis* incubated in culture medium with different NaCl content. (**b**) Survival ability determination of the *V. brasiliensis* cultivated in different NaCl content medium. Values are means of three independent experiments. Different lowercase letters denote significant differences (*p* < 0.05).

To study the effect of salt stress on the growth and survival of *V. brasiliensis*, growth rates of bacteria cultured in medium containing various NaCl concentrations are shown in Figure 1a. It was found that *V. brasiliensis* can survive without NaCl, as well as salinity as high as 7%. Previous experiments found that when salinity reached 9% or above, growth of *V. brasiliensis* was inhibited completely (Data not shown). Different strains of *Vibrio* spp. exhibited different salt tolerance. For example, pathogenic *V. parahaemolyticus*, a moderately halophilic and salt-requiring microorganism, cannot reproduce in a salt-free environment, but can grow in NaCl concentrations up to 8% [21].

Under salt conditions at 1% NaCl, 2% NaCl, and 3% NaCl, there was almost no lag phase after inoculation of *V. brasiliensis*, indicating that the strains quickly adapted to the new environment and began to grow exponentially. In addition, the log phase growth of *V. brasiliensis* was faster in 2% NaCl and 3% NaCl medium. However, for the cells grown in 0%, 5% and 7% NaCl, lag phase of the cells was longer, especially the cells cultured in 7% NaCl, it entered the logarithmic growth phase 12 h after inoculation, as seen in other *Vibrio* genus bacteria under salt stress [14]. This indicated that salinity had a certain effect on the adaptation of *V. brasiliensis* to the environment, making it stay longer in the adjustment period and grow slowly in the logarithmic phase. NaCl plays an important role in maintaining the osmotic pressure balance of cells. These findings suggested that lower salinities (2–3% NaCl) are most suitable or the growth and survival of *V. brasiliensis.* During salt stress when salt concentration is low, cells are in low osmotic pressure environment, causing the transportation of key substances exchanged between the cells and the environment cannot be reached since cell growth is affected. When salt concentration in the culture was 7%, cells were under high osmotic pressure conditions, material transportation were hindered, intracellular enzyme activity was reduced, metabolism was slow, and cell growth was restricted. The results showed that bacteria are able to adapt to survival in salt stress, suggesting that there should be a response regulation system to adjust different osmotic stresses [14]. The cell enumeration of *V. brasiliensis* cultivated in different NaCl content is shown in Figure 1b. Similar to the growth curve results, bacteria cultured in 2% and 3% NaCl medium showed the highest survival ability. The cell number of *V. brasiliensis* grew in culture medium with addition of 1%, 5% NaCl, and 7% NaCl were reduced significantly (*p* < 0.05). Additionally, the survival ability of cell cultured in 7% NaCl was reduced, mainly due to inhibition of cell growth in high salt condition. Previous studies using different concentrations of NaCl to culture *V. parahaemolyticus* showed that the survival ability of *Vibrio* gradually decreased with increasing (>6% NaCl) or decreasing (<1.0% NaCl) salinity [30]. And some studies shown that different strains of *V. cholerae* can tolerate the different maximum NaCl concentration. Growth curves shown that most of the strains can growth well in 0.5% to 5% NaCl. However, some strains barely grow or have a noticeable growth delay in 5% and 6% NaCl [31].

### 3.2. Morphological Analysis of V. brasiliensis in Response to Salt Stress

**Figure 2:**
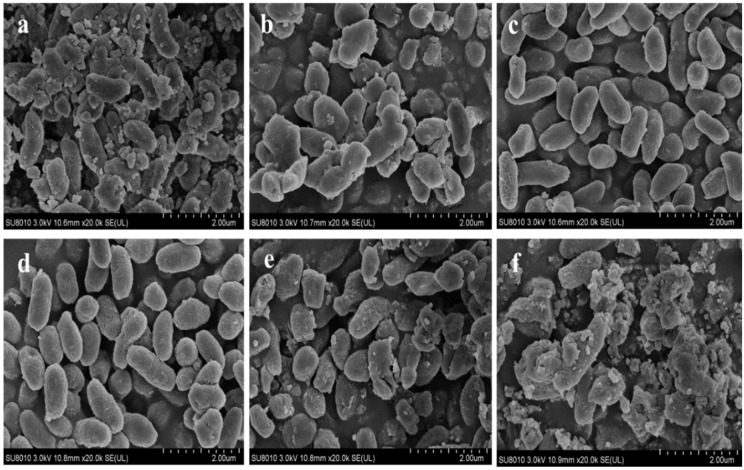
Morphological of *V. brasiliensis* cells after cultivation in different NaCl concentrations. (**a**) Cells cultured in without NaCl medium; (**b**) cells cultured in 1% NaCl medium; (**c**) cells cultured in 2% NaCl medium (optimal growth NaCl content of *V. brasiliensis*); (**d**) cells cultured in 3% NaCl medium; (**e**) cells cultured in 5% NaCl medium; (**f**) cells cultured in 7% NaCl medium.

Scanning electron micrograph (SEM) revealed that the 2% NaCl and 3% NaCl content medium cultivated *V. brasiliensis* were mainly short rods, but 2% NaCl cultured cells surface was relatively smoother and exhibited better cell integrity than 3% NaCl (Figure 2c,d). Bacteria cultured in medium without NaCl, most cells had lost their integrity, resulting in cell debris as shown in Figure 2a. When the cells are in a low osmotic pressure environment, the cells tend to swell and break due to water absorption. However, significant structural changes, including rapture of cells and deformation of cells were observed after cultured in 1% NaCl (Figure 2b). When cultured in medium containing 5% and 7% NaCl, the cells lost their original shape and cells appeared to be lysed and ruptured to varying degrees, (Figure 2e). The medium containing 7% NaCl has led to complete cellular deformation (Figure 2f).

### 3.3. Effect of Salt Stress on the Swimming Ability of V. brasiliensis

**Figure 3:**
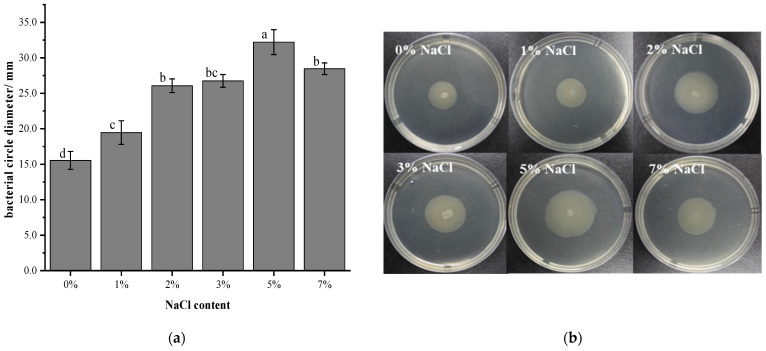
Swimming motility of *V. brasiliensis* under different NaCl concentrations. (**a**) 0.3% agar plates bacteria circle diameter (mm) were measured after cultivated in various NaCl medium, and the value is represented by three repetitions mean ± sd. Different lowercase letters denote significant differences (*p* < 0.05); (**b**) 0.3% agar medium obtained the swimming circle of *V. brasiliensis* cultured in various NaCl concentrations.

Swimming motility of *V. brasiliensis* affected by salt stress were determined by measuring the diameter of the circle on the agar plate. Results in Figure 3 showed that bacteria cultured in 0% NaCl had the slowest motility among the test groups, and as the concentration of NaCl increased from 0% to 5%, the diameter of the bacterial circle also increased gradually. However, when the NaCl concentration was greater than 5%, the diameter was decreased. In addition, the diameter of the circle formed by *V. brasiliensis* grown in 7% NaCl medium was higher than 2%, 3% NaCl, and the bacterial circle diameter of the bacteria grown in 2% NaCl was less than that cultured in 3% NaCl, but there are no significant differences between them (*p* > 0.05). This result indicated that the concentration of NaCl has an effect on the swimming ability of *V. brasiliensis*. When grown in low NaCl concentration condition, the swimming ability of *V. brasilienis* was poor. Previous studies have shown that the *V. salmonicida* cells grown in 2.5% NaCl possess a higher motility than thsoe grown at 1% NaCl [32].

The effect of NaCl on the swimming ability of *V. brasiliensis* is mainly caused by the difference of Na^+^ concentration [33]. Studies indicating that Na^+^ drives the flagellum of *V. cholerae*, *V. parahaemolyticus* [34] and *V. alginolyticus* [35], mainly due to that the swimming ability of bacteria that use Na^+^ to drive their flagellar motors. Without NaCl, these is not enough sodium ion to drive their flagellar motors, which is the reason why *V. brasiliensis* has diminished swimming motility. However, the swimming ability of bacteria cultured with 3%, 5%, and 7% NaCl medium are higher than that of bacteria cultured with 2% NaCl. This may be due to more Na^+^ has led to swimming toward favorable environments in media through reversibly rotating the flagellar filaments [36].

### 3.4. Analysis of Differentially Expressed OMPs in Response to Salt Stress

**Figure 4:**
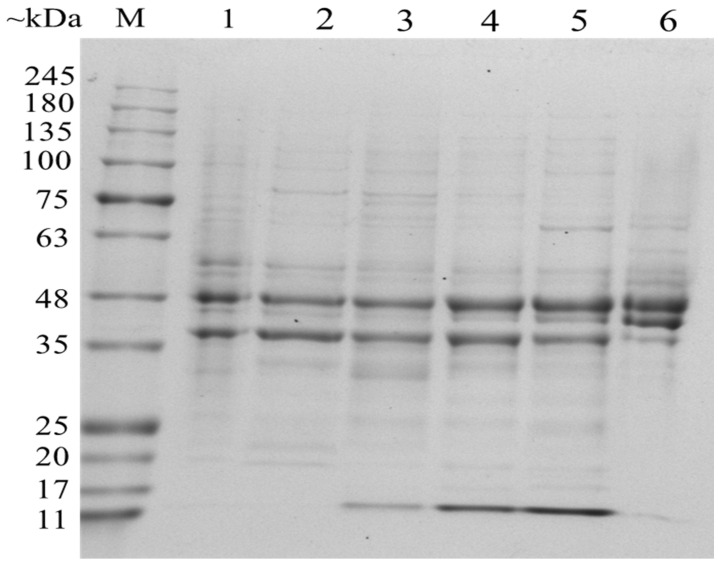
SDS-PAGE of OMPs of *V. brasiliensis* cultured in culture medium with different NaCl concentration. M: marker; lane 1, OMPs of *V. brasiliensis* cultured in 0% NaCl medium; lane 2, OMPs of *V. brasiliensis* cultured in 1% NaCl medium; lane 3, OMPs of *V. brasiliensis* cultured in 2% NaCl medium; lane 4, OMPs of *V. brasiliensis* cultured in 3% NaCl medium; lane 5, OMPs of *V. brasiliensis* cultured in 5% NaCl medium; lane 6, OMPs of *V. brasiliensis* cultured in 7% NaCl medium.

OMPs are special proteins that only exist in Gram-negative bacteria; they are composed of lipopolysaccharides (LPS), phospholipids (PLs), outer membrane proteins (OMPs) and lipoproteins (Lpp) [37]. OMPs play a vital role in material transportation of Gram-negative bacteria, ensuring the integrity of the outer membrane, sensing changes in the external environment, and signal transduction [38]. To examine the effect of NaCl on protein expression of the OMPs, SDS-PAGE electrophoresis was performed to analyze protein profiles of *V. brasiliensis* cultured in various NaCl concentration. As shown in Figure 4, two major protein bands with molecular weight in the range between 35 and 48 kDa (~48 kDa and ~37 kDa) with similar concentration and distribution have been expressed in most of the cultures, other than in cultures with 7% NaCl. The most obvious differences are expression of proteins around 15 kDa of *V. brasiliensis* cultured in 2%, 3%, and 5% NaCl, as well as the expression of protein with molecular weight around 55 kDa in cultures without NaCl. In addition, OMPs with molecular weight around 45 kDa cultured in 7% NaCl have higher protein expression than other NaCl concentration.

Identification of differentially expressed OMPs was performed by mass spectrometry. Some conserved OMPs were found (Table S1), the expression of these OMPs not affected by NaCl concentration, that is, these OMPs of *V. brasiliensis* are all expressed under various NaCl medium. Furthermore, there are OMPs that have porin activity in these conserved proteins, for example, outer membrane protein OmpU (*VIBR0546_08029*), outer membrane protein OmpA (*VIBR0546_09484*), Vitamin B12 transporter BtuB (*btuB*) and outer membrane protein (*VIBR0546_02985*). Among them, the OmpU is a major component of outer membrane, accounting for 60% of the outer membrane proteins [39]. Their conservation in *Vibrio* spp. suggesting it is importance to cell’s physiological processes. Previous studies suggesting that the OmpU seems to act as a sensor component in a signal transduction pathway in *Vibrio* [40]. But the synthesis of the OmpU protein is regulated by NaCl content of the cultured medium, OmpU accounts for 30% of the total outer membrane protein when *V. cholerae* cultured in 1.0% NaCl medium and 60% in without NaCl [39]. Besides, the outer membrane protein OmpK with nucleoside transmembrane transport protein activity is highly conserved in *Vibrio*, which has been recognized [41]. There have some proteins with efflux transmembrane transporter activity also conserved in various NaCl stress cultured, such as outer membrane channel protein (*tolC*), outer membrane protein (*VIBR0546_13282*), and outer membrane protein (*VIBR0546_20323*) that have not been reported.

However, the OMPs of *V. brasiliensis* changed when grown in different NaCl content medium, suggesting the bacterium can rapidly responding to salt stress through changes in outer membrane proteins. The results in Table 1 show that under NaCl stress conditions *V. brasiliensis* may need to regulate substances transport through special expression of its outer membrane proteins [42]. The expression of outer membrane proteins associated with bacterial pilus is affected by NaCl stress, and we found that Flp pilus assembly protein CpaB (*VIBR0546_18016*) and MSHA biogenesis protein MshL (*VIBR0546_05478*) only expressed when grown in 2% NaCl. The penicillin-binding protein activator LpoA was found in 2% NaCl and 3% NaCl medium cultured *V. brasiliensis*, respectively, which participated in the peptidoglycan biosynthetic process of bacterial and it was important for maintenance of cell shape and survival [43]. Such result explained that *V. brasiliensis* have typical *Vibrio* morphology when bacteria grown in the 2% NaCl and 3% NaCl medium. It should be pointed out that the outer membrane protein of sodium-type flagellar protein MotY (*VIBR0546_10974*), the component of the polar flagellar rotary motor is found only when the bacteria grown in 3%, 5%, and 7% NaCl. It is noteworthy that ferric aerobactin receptor protein was only found in 7% NaCl cultured bacteria’s outer membrane proteins, this may be due to that high salt stress affects iron absorption, and the expression of ferric aerobactin receptor protein is activated to improve iron utilization of the cells under high salt stress environment [44,45]. In addition, the putative long-chain fatty acid transport protein (*VIBR0546_07642*) has disappeared when bacteria cultivated in 5% and 7% NaCl stress medium, on the contrary, this protein has appeared when *V. parahaemolyticus* and *V. alginolyticus* were treated with Ultraviolet-C radiation stress [46]. Therefore, the OMPs that changes in salt stress can be considered as response salt stress proteins in *V. brasiliensis* and types of protein expressed in response to salt stress are different.

### 3.5. Transcriptome Analysis

**Figure 5:**
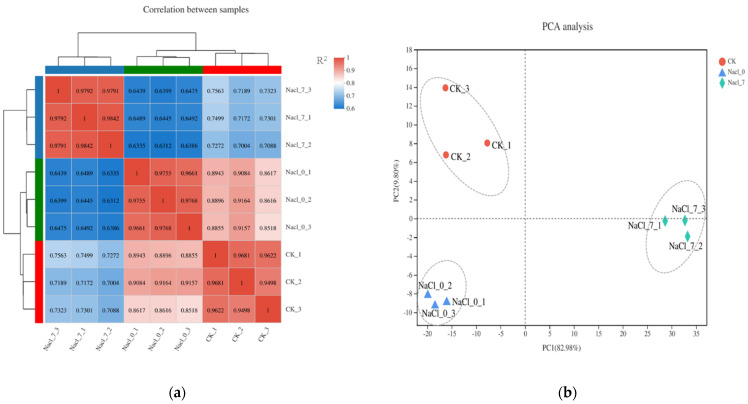
Correlation analysis between samples (**a**) and principal component analysis (PCA) of samples (**b**) of *V. brasiliensis* under different NaCl concentrations cultured. (**a**) Both the abscissa and the ordinate indicated the names of samples under different processing conditions, and each sample was set with three biological replicates. CK represent 2% NaCl concentration and as control; Nacl_0 represent 0% NaCl concentration; Nacl_7 represent 7% NaCl concentration. The shade of the square in the figure represents the correlation coefficient R^2^. Red indicates positive correlation, and blue indicates lower the correlation; (**b**) the three dots with the same color represent three biological replicates in each group.

It can be seen from Figure 5a that the correlation coefficient of CK (2% NaCl) of the control group samples were greater than 0.95, the correlation coefficient of the experimental group Nacl_0 (0% NaCl) samples were greater than 0.96, and the correlation coefficient of the experimental group Nacl_7 (7% NaCl) samples were greater than 0.98. The correlation coefficients of the repeated samples in the above groups were all above 0.95, indicated that the three biological replicate samples in each group had high similarity, therefore the transcription and sequencing results of the samples in each group should be reliable. In addition, the correlation coefficient of samples between different groups was relatively small, demonstrating that NaCl concentration had affected gene expression levels within *V. brasiliensis*. Similarly, PCA analysis in Figure 5b shows the repeated samples of each group have good correlation, which indicates the experimental data of *V. brasiliensis* transcriptome cultured in different NaCl are highly accurate.

**Figure 6:**
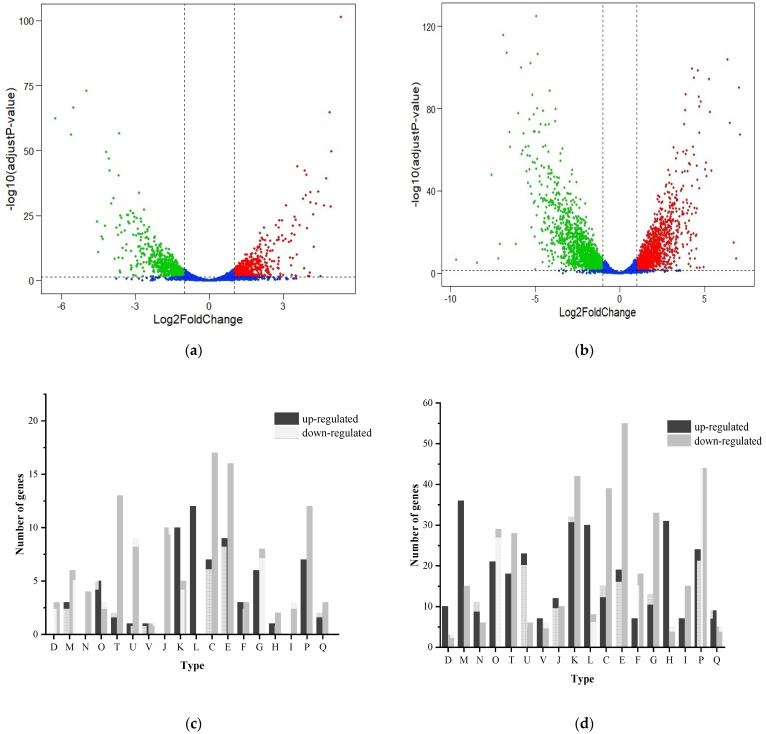
The volcano plots of genes and COG functional annotations between different treatment. (**a**) Volcano plots displaying differential expressed genes between 0% NaCl and 2% NaCl cultured *V. brasiliensis*; (**b**) Volcano plots displaying differential expressed genes between 7% NaCl and 2% NaCl cultured *V. brasiliensis*. (Screening criteria for differentially expressed genes: adjust *p* value < 0.05, Abs (log_2_Fold Change) ≥ 1); (**c**,**d**) COG functional annotations of differentially expressed genes identified in 0% NaCl and 7% NaCl cultured bacteria, respectively. (Abs (log_2_Fold Change) ≥ 2). COG functional description represented by capital letters. D: Cell cycle control, cell division, chromosome partitioning; M: Cell wall/membrane/envelope biogenesis; N: Cell motility; O: Posttranslational modification, protein turnover, chaperones; T: Signal transduction mechanisms; U: Intracellular trafficking, secretion, and vesicular transport; V: Defense mechanisms; J: Translation, ribosomal structure and biogenesis; K: Transcription; L: Replication, recombination and repair; C: Energy production and conversion; E: Amino acid transport and metabolism; F: Nucleotide transport and metabolism; G: Carbohydrate transport and metabolism; H: Coenzyme transport and metabolism; I: Lipid transport and metabolism; P: Inorganic ion transport and metabolism; Q: Secondary metabolites biosynthesis, transport and catabolism; S: Function unknown (not shown in the picture).

The *V. brasiliensis* makes a series of responses to changes in the external environment by regulating its own gene expression. We performed transcriptome analysis of *V. brasiliensis* which cultured under 0% NaCl and 7% NaCl respectively, to isolate genes associated with salt stress. Compared to *V. brasiliensis* cultured in 2% NaCl, 1230 genes (577 up-regulated and 653 down-regulated) and 2504 genes (1215 up-regulated and 1289 down-regulated) were observed after 0% NaCl and 7% NaCl treatments, respectively (Figure 6a,b, Tables S2 and S3). It is interesting to note that the number of differentially expressed genes in 7% NaCl was significantly higher than in 0% NaCl, suggesting that when *V. brasiliensis* grown in 7% NaCl medium, this high concentration salt environment will generate greater stress to cell growth so that the bacteria by up-regulated and down-regulated self-genes in response to osmotic stress caused by high salt [31]. In order to describe the types and functions of genes with significant transcriptional changes more intuitively, COG function annotation analysis (Figure 6c,d) shows that the genes related to replication, recombination and repair of *V. brasiliensis* in 0% NaCl and 7% NaCl are notably up-regulated, indicating that under the stress of low or high osmotic stress, bacteria can activate their own repair mechanism to response the unfavorable environment. In addition, for genes involved in signal transduction mechanisms, cell motility, energy production and transformation, amino acid transport and metabolism, carbohydrate transport and metabolism, inorganic ion transport and metabolic functions, and the number of down-regulated genes was higher than those of up-regulated genes. This may be due to cells in unfavorable growth environment and most of the metabolism in the cell is blocked. To further obtain the genes that respond to salt stress, we integrated the genes with transcriptional changes and found that two sigma factors, including the RNA polymerase sigma factor RpoS (*VIBR0546_05309*) and putative anti-sigma F factor antagonist (*VIBR0546_10019*) were down-regulated in 0% NaCl and 7% NaCl stress. The anti-RNA polymerase sigma 70 factor down-regulated in 7% NaCl, showed in high osmotic stress the RNA polymerase sigma 70 factor can function normally (Table S4). In Gram-negative bacteria, the RNA polymerase sigma 70 factor plays a key role in responses to osmotic stress [47,48].

However, bacteria in low and high osmotic environment have distinct responses stress mechanism, through analysis the differentially expressed genes (selection criteria: Abs (log_2_FC) ≥ 1, *p* < 0.05) related to stress (Table 2 and Table 3). Changes in expression levels of salt stress-response genes in *V. brasiliensis* were concentration-specific, suggesting that the bacteria stress response system is complicated. We found that the same gene have opposite up-regulated and down-regulated expression in two treatments, indicate that this type of gene is regulated differently in low and high osmotic stress. The osmolarity response regulator (*ompR*), universal stress protein VspE, universal stress protein family 8, universal stress protein A, and NptA protein genes were all up-regulated in 0% NaCl medium but down-regulated in 7% NaCl, demonstrates that these genes can response to the low osmotic pressure environment outside the cell, which are conducive to the survival of bacteria in the low osmotic stress conditions. NptA protein gene (*VIBR0546_00894*) have phosphate ion transmembrane transporter activity and sodium: phosphate symporter activity function and involved in sodium-dependent phosphate transport process. Besides, previous studies of NptA in *V. cholerae* suggested that this protein may contribute to *V. cholerae* colonization in host [49], and further research has determined the stress response of NptA that contributes to the growth or stress resistance in *V. vulnificus* [50]. Up-regulation of such genes was in response to 0% NaCl stress. However, it was down-regulated in 7% NaCl conditions might show that this gene was inhibited under a higher osmotic pressure, and for that reason the growth and viability of *V. brasiliensis* are significantly reduced. In addition, there are some universal stress protein genes that have the same expressive characteristics as the genes mentioned above, which are up-regulated in 0% NaCl medium but are down-regulated under high osmotic pressure of 7% NaCl. The universal stress protein UspE gene, encode protein belongs to the universal stress proteins (USP) family. The UspE protein is important for cellular motility, and study demonstrates that *uspE* mutants are devoid of flagella in *E. coli* [51], and the UspE proteins enable cells to defense and escape environmental stress.

As shown previously, NaCl concentrations have influence on outer membrane protein gene transcription level, we found that expression of genes encoding outer membrane protein W (OmpW) (*VIBR0546_07217*), outer membrane protein OmpA (*VIBR0546_09484*), and outer membrane protein OmpK (*VIBR0546_05598*) were all down-regulated in 0% and 7% NaCl conditions. The ability in salt-tolerance was increased in *E. coli* overexpressed OmpW, the finding suggests that OmpW is necessary for environmental salt regulation in bacteria [52]. Previous studies have shown that bacteria in different salt stress can response to the cells osmotic imbalance through transporter system. The Na^+^/H^+^ antiporter, which is a sodium ion channel commonly used by cells and generate the electrochemical potential for the Na^+^ driven flagella motor by extruding Na^+^. The key gene, *motY* (*VIBR0546_10974*), for the rotation of the Na^+^ driven motor was found to be up-regulated in high NaCl (7% NaCl) medium, and the sodium-type flagella protein MotY was found in 3%, 5%, and 7% NaCl cultured bacteria’s outer membrane proteins, respectively. Furthermore, the Na^+^/H^+^ antiporter gene (*VIBR0546_21150*) was up-regulated as well, so when the bacteria cultured under higher NaCl concentrations, the higher Na^+^ concentration in the environment will give cell higher swimming ability. However, when the NaCl concentration is too high, it will cause fatal stress to growth and survival of *V. brasiliensis*, which diminishes its swimming ability. Although putative Na^+^/H^+^ antiporter gene (*VIBR0546_01129*) expression was also up-regulated in 0% NaCl cultured *V. brasiliensis*, the up-regulation of *motY* gene and the presence of sodium-type flagella protein MotY were not found, indicating that the Na^+^/H^+^ antiporter ultimately drives flagella rotation though the MotY protein [35].

## 4. Conclusions

Based on the different NaCl concentrations on the growth rate, survival, cell swimming ability, cell outer membrane proteins, and the transcriptome of *V. brasiliensis* were caried out to investigate the salt stress to the bacteria and cells responses to stress in this study. The results showed that 0–5% NaCl concentration had little effect on *V. brasiliensis* growth and survival, but 7% NaCl had a great influence on the bacteria. The NaCl concentration of the medium has an effect on the cellular morphology of *V. brasiliensis*, and the cells’ morphology were severely damaged under low-salt and high-salt stress cultures. The Penicillin-binding protein activator LopA OMP was only found in the cells cultured in 2% and 3% NaCl, and the protein contributed to maintaining the integrity of the cells. The OMP of Sodium-type flagellar protein MotY was found in the cells cultured with 3%, 5%, and 7% NaCl, indicating that *V. brasiliensis* produced this protein in response to external high-salt stress. Furthermore, some key genes in response to salt stress were discovered, such as NptA protein gene (*VIBR0546_00894*), osmolarity response regulator gene (*ompR*), putative threonine efflux protein gene (*VIBR0546_17048*) and Na^+^/H^+^ antiporter gene (*VIBR0546_21150*) and so on. For survival, *V. brasiliensis* responds to salt stress through differential gene expression. This study is conducive to understanding how *V. brasiliensis* responds to salt stress in the environment.

## Figures and Tables

**Table 1:** The special expression outer membrane proteins of *V. brasiliensis* in different NaCl concentrations cultured.

NaCl (%)	Accession	Gene	Proteins	Mw [KDa]	Molecular Function
2	E8LNG0	lpoA	Penicillin-binding protein activator LpoA	67.3	enzyme regulator activity
2	E8LPR8	VIBR0546_18016	Flp pilus assembly protein CpaB	27.2	NA
2	E8LQ55	VIBR0546_05478	MSHA biogenesis protein MshL	59.2	NA
3	E8LNG0	lpoA	Penicillin-binding protein activator LpoA	67.3	enzyme regulator activity
3	E8LS16	VIBR0546_10974	Sodium-type flagellar protein MotY	33.5	NA
5	E8LS16	VIBR0546_10974	Sodium-type flagellar protein MotY	33.5	NA
5	E8M0I9	VIBR0546_09489	Outer membrane protein OmpA	35.1	porin activity
7	E8LS16	VIBR0546_10974	Sodium-type flagellar protein MotY	33.5	NA
7	E8M0I9	VIBR0546_09489	Outer membrane protein OmpA	35.1	porin activity
7	E8LX27	VIBR0546_17898	Ferric aerobactin receptor	77.7	siderophore uptake transmembrane transporter activity; signaling receptor activity

NA, it means there is no such content.

**Table 2:** The up and down-regulated genes of *V. brasiliensis* cultured in 0% NaCl medium.

Gene Name	Gene Description	FC	Log2FC	*p* Value	Regulate
VIBR0546_12042	proton/glutamate symporter	5.165	2.369	1.15 × 10^−13^	up
nhaA	pH-dependent sodium/proton antiporter	3.672	1.877	1.61 × 10^−12^	up
VIBR0546_00894	NptA protein	11.824	3.564	5.86 × 10^−12^	up
VIBR0546_21825	putative outer membrane protein	7.040	2.816	6.8 × 10^−11^	up
VIBR0546_00270	universal stress protein UspE	2.950	1.561	6.62 × 10^−8^	up
VIBR0546_01129	putative Na^+^/H^+^ antiporter	3.182	1.670	1.85 × 10^−7^	up
VIBR0546_09929	universal stress protein family 8	2.511	1.328	4.65 × 10^−6^	up
VIBR0546_10589	universal stress protein A	2.881	1.527	2.87 × 10^−5^	up
ompR	osmolarity response regulator	2.471	1.305	9.71 × 10^−5^	up
VIBR0546_03992	putative salt-induced outer membrane protein	2.567	1.360	4.52 × 10^−4^	up
VIBR0546_17778	OmpR family two-component response regulator	2.039	1.028	9.87 × 10^−3^	up
VIBR0546_02830	choline/carnitine/betaine transporter	0.079	−3.660	3.18 × 10^−60^	down
VIBR0546_00510	outer membrane protein	0.081	−3.631	7.45 × 10^−24^	down
VIBR0546_06992	sodium/proline symporter	0.097	−3.366	8.10 × 10^−14^	down
VIBR0546_03902	putative sodium/sulfate symporter	0.204	−2.295	2.04 × 10^−13^	down
VIBR0546_13277	Outer membrane protein	0.263	−1.926	5.11 × 10^−12^	down
VIBR0546_09484	outer membrane protein OmpA	0.250	−2.000	4.01 × 10^−11^	down
VIBR0546_07217	outer membrane protein W	0.253	−1.984	4.35 × 10^−11^	down
VIBR0546_05877	OmpA family protein	0.293	−1.772	1.04 × 10^−10^	down
VIBR0546_18581	sodium/alanine symporter	0.383	−1.385	8.86 × 10^−9^	down
VIBR0546_13282	outer membrane protein	0.396	−1.338	1.58 × 10^−6^	down
VIBR0546_03420	putative outer membrane protein	0.370	−1.434	2.10 × 10^−5^	down
VIBR0546_02985	Outer membrane protein	0.262	−1.933	9.81 × 10^−5^	down
VIBR0546_16166	putative arginine/ornithine antiporter	0.478	−1.066	2.62 × 10^−3^	down

**Table 3:** The up and down-regulated genes of *V. brasiliensis* cultured in 7% NaCl medium.

Gene Name	Gene Description	FC	Log2FC	*p* Value	Regulate
VIBR0546_17048	putative threonine efflux protein	26.152	4.709	1.9 × 10^−43^	up
VIBR0546_21150	Na^+^/H^+^ antiporter	9.496	3.247	4.47 × 10^−36^	up
VIBR0546_01064	Ca^2+^/Na^+^ antiporter	10.100	3.336	5.15 × 10^−32^	up
VIBR0546_01411	outer membrane protein	4.147	2.052	1.00 × 10^−30^	up
VIBR0546_01241	proton/glutamate symport protein	4.243	2.085	2.23 × 10^−17^	up
VIBR0546_05214	LPS assembly outer membrane complex protein LptD	2.683	1.424	6.98 × 10^−9^	up
VIBR0546_21825	putative outer membrane protein	3.554	1.830	1.01 × 10^−7^	up
VIBR0546_10974	sodium-type flagellar protein MotY precursor	2.906	1.539	1.09 × 10^−7^	up
VIBR0546_21600	putative sodium-type flagellar protein MotY precursor	6.971	2.801	5.54 × 10^−6^	up
VIBR0546_03992	putative salt-induced outer membrane protein	2.468	1.304	3.57 × 10^−5^	up
VIBR0546_06697	sodium/glutamate symporter	2.705	1.435	1.36 × 10^−4^	up
VIBR0546_12742	sodium/alanine symporter	2.326	1.218	2.10 × 10^−4^	up
VIBR0546_12482	putative outer membrane protein	2.272	1.184	7.17 × 10^−4^	up
VIBR0546_07217	outer membrane protein W	0.008	−6.889	9.60 × 10^−120^	down
VIBR0546_05309	RNA polymerase sigma factor RpoS	0.028	−5.163	7.74 × 10^−90^	down
VIBR0546_00510	outer membrane protein	0.038	−4.726	7.75 × 10^−47^	down
VIBR0546_20323	outer membrane protein	0.062	−4.007	3.32 × 10^−37^	down
VIBR0546_00505	putative anti-sigma regulatory factor	0.030	−5.066	5.08 × 10^−36^	down
VIBR0546_18581	sodium/alanine symporter	0.171	−2.552	1.12 × 10^−19^	down
VIBR0546_09929	universal stress protein family 8	0.160	−2.647	3.93 × 10^−19^	down
VIBR0546_00270	universal stress protein UspE	0.221	−2.179	3.5 × 10^−15^	down
VIBR0546_12042	proton/glutamate symporter	0.230	−2.123	8.13 × 10^−15^	down
VIBR0546_09484	outer membrane protein OmpA	0.232	−2.110	1.90 × 10^−13^	down
VIBR0546_10579	universal stress protein UspB	0.249	−2.005	1.88 × 10^−11^	down
VIBR0546_06992	sodium/proline symporter	0.111	−3.171	6.54 × 10^−10^	down
VIBR0546_10589	universal stress protein A	0.245	−2.026	3.43 × 10^−7^	down
ompR	osmolarity response regulator	0.365	−1.452	5.85 × 10^−7^	down
VIBR0546_00894	NptA protein	0.231	−2.116	1.03 × 10^−4^	down
VIBR0546_05877	OmpA family protein	0.427	−1.229	1.20 × 10^−4^	down
VIBR0546_05598	outer membrane protein OmpK	0.420	−1.252	1.06 × 10^−3^	down
VIBR0546_01821	Outer membrane protein	0.217	−2.202	2.26 × 10^−3^	down

## Data Availability

Data are contained within the article.

## Supplementary Materials

The following supporting information can be downloaded at: https://www.mdpi.com/article/10.3390/foods11060840/s1, Table S1: OMPs of *V. brasiliensis* all expressed in different NaCl concentrations, Table S2: Stress-related differentially expressed genes screened by *V. brasiliensis* cultured in 0% NaCl. (Screening criteria: Abs (log2FC) ≥ 1, *p* < 0.05), Table S3: Stress-related differentially expressed genes screened by *V. brasiliensis* cultured in 7% NaCl. (Screening criteria: Abs (log2FC) ≥ 1, *p* < 0.05), Table S4: Differentially expressed sigma factors of *V. brasiliensis* cultured with different NaCl concentrations in transcriptome analysis.
