# Supplementary material for: Effects of NaCl Concentration on the Behavior of Vibrio brasiliensis and Transcriptome Analysis
Source: Foods. 2022 Mar 15;11(6):840. doi: 10.3390/foods11060840 (PMC8955013; doi:10.3390/foods11060840)
Supplement: Supplementary file 1 [file foods-11-00840-s001.zip › foods-1625195-supplementary.pdf]

foods

Supplementary Materials

Effects of NaCl Concentration on the Behavior of *Vibrio brasiliensis* and Transcriptome Analysis

Shuyang Hu, Yuwei Li, Boran Wang, Lijun Yin, and Xin Jia *

College of Food Science and Nutritional Engineering, China Agricultural University, Beijing 100083, China; hushuyangyllh@163.com (S.H.); liyuwei9@126.com (Y.L.); wang_remon@163.com (B.W.); ljyin@cau.edu.cn (L.Y.)
* Correspondence: xinjia@cau.edu.cn

Table S1. OMPs of *V. brasiliensis* all expressed in different NaCl concentrations.

Accession	Gene	Proteins	Mw [KDa]	Molecular Function	Biological process
E8LZA2	VIBR0546_21035	OMPH_PHOPR porin-like protein H	36.9	porin activity	NA
E8LYH6	VIBR0546_02985	Outer membrane protein	35.4	porin activity	NA
E8LXL0	lpp	Major outer membrane lipoprotein Lpp	8.6	lipid binding; peptidoglycan binding	lipid modification; periplasmic space organization
E8LNC0	tolC	Outer membrane channel protein	47.1	efflux transmembrane transporter activity	NA
E8LPF1	VIBR0546_13282	Outer membrane protein	48.4	efflux transmembrane transporter activity	NA
E8LR24	VIBR0546_08029	Outer membrane protein OmpU	36.8	porin activity	NA
E8LY62	VIBR0546_20323	Outer membrane protein	48.4	efflux transmembrane transporter activity	NA
E8M0I8	VIBR0546_09484	Outer membrane protein OmpA	34.9	porin activity	ion transport
E8M095	btuB	Vitamin B12 transporter BtuB	66.3	ABC-type vitamin B12 transporter activity; porin activity	NA
E8LVK2	lptD	LPS-assembly protein LptD	89.1	NA	Gram-negative-bacterium-type cell outer membrane assembly; lipopolysaccharide transport; response to organic substance
E8LQ79	VIBR0546_05598	Outer membrane protein OmpK	31.4	nucleoside transmembrane transporter activity	NA

Table S2. Stress-related differentially expressed genes screened by *V. brasiliensis* cultured in 0% NaCl. (Screening criteria: Abs(log2FC) ≥ 1, $p < 0.05$).

Gene_id	Gene name	Gene description	FC(Nacl_0/CK)	Log2FC(Nacl_0/CK)	*p* Value	*p* Adjust	Signif-icant	Regu-late	Type
VIBR0546_16703	VIBR0546_16703	dihydrolipoamide dehydrogenase	40.593	5.34317	1E-105	6E-102	yes	up	mRNA
VIBR0546_02810	VIBR0546_02810	choline dehydrogenase	0.032	−4.98029	5.2E-77	1.1E-73	yes	down	mRNA
VIBR0546_06827	VIBR0546_06827	hypothetical protein	0.022	−5.50496	2.3E-70	3.2E-67	yes	down	mRNA
VIBR0546_14395	VIBR0546_14395	hypothetical protein	29.563	4.88571	2.5E-68	2.7E-65	yes	up	mRNA
VIBR0546_02805	VIBR0546_02805	glycine betaine/L-proline ABC transporter glycine betaine/L-proline-binding protein	0.013	−6.23119	6.3E-66	5.4E-63	yes	down	mRNA
VIBR0546_02830	VIBR0546_02830	choline/carnitine/betaine transporter	0.079	−3.65964	3.2E-60	2.3E-57	yes	down	mRNA
VIBR0546_02815	VIBR0546_02815	betaine aldehyde dehydrogenase	0.021	−5.60738	1.1E-59	6.5E-57	yes	down	mRNA
VIBR0546_06837	VIBR0546_06837	hypothetical protein	31.048	4.95643	5.7E-53	3.1E-50	yes	up	mRNA
VIBR0546_10034	VIBR0546_10034	chemoreceptor glutamine deamidase CheD	0.056	−4.16915	8.4E-53	4E-50	yes	down	mRNA
VIBR0546_10024	VIBR0546_10024	methyl-accepting chemotaxis protein	0.06	−4.05436	3.7E-50	1.6E-47	yes	down	mRNA
VIBR0546_09924	VIBR0546_09924	6-phospho-beta-glucosidase	12	3.58495	4.7E-47	1.8E-44	yes	up	mRNA
VIBR0546_09002	VIBR0546_09002	NADH dehydrogenase subunit II-related protein	14.83	3.89042	1.6E-45	5.9E-43	yes	up	mRNA
VIBR0546_10044	VIBR0546_10044	methyl-accepting chemotaxis protein	0.061	−4.04626	2.2E-45	7.2E-43	yes	down	mRNA
VIBR0546_13825	secD	preprotein translocase subunit SecD	15.529	3.95689	6.7E-44	2E-41	yes	up	mRNA
VIBR0546_10029	VIBR0546_10029	protein-glutamate methylesterase CheB	0.078	−3.6797	1.3E-43	3.7E-41	yes	down	mRNA
VIBR0546_11807	VIBR0546_11807	putative glutamine synthetase	26.766	4.74231	2.2E-42	5.9E-40	yes	up	mRNA
VIBR0546_06707	VIBR0546_06707	ATP-dependent OLD family endonuclease	21.467	4.42408	3.3E-37	8.4E-35	yes	up	mRNA
VIBR0546_11812	VIBR0546_11812	putative glutamine amidotransferase	17.127	4.09823	5.2E-37	1.2E-34	yes	up	mRNA
VIBR0546_02279	rpsA	30S ribosomal protein S1	0.139	−2.84291	1E-36	2.3E-34	yes	down	mRNA
VIBR0546_11837	VIBR0546_11837	hypothetical protein	15.292	3.9347	6.7E-36	1.4E-33	yes	up	mRNA
VIBR0546_20333	VIBR0546_20333	Hcp protein	0.068	−3.88596	1.3E-34	2.6E-32	yes	down	mRNA
VIBR0546_03180	malE	maltose ABC transporter periplasmic protein	14.047	3.81223	2.8E-34	5.6E-32	yes	up	mRNA
VIBR0546_11802	VIBR0546_11802	hypothetical protein	17.22	4.10605	4.6E-33	8.7E-31	yes	up	mRNA
VIBR0546_13875	VIBR0546_13875	putative cation efflux system component	0.064	−3.97556	1.5E-32	2.7E-30	yes	down	mRNA
VIBR0546_16698	VIBR0546_16698	peroxiredoxin family protein/glutaredoxin	20.209	4.33695	2.3E-32	3.9E-30	yes	up	mRNA
VIBR0546_11842	VIBR0546_11842	succinate-semialdehyde dehydrogenase	8.653	3.11323	9.7E-32	1.6E-29	yes	up	mRNA
VIBR0546_07032	VIBR0546_07032	hypothetical protein	25.246	4.65796	1E-31	1.6E-29	yes	up	mRNA
VIBR0546_09914	VIBR0546_09914	hypothetical protein	30.786	4.9442	3.5E-31	5.3E-29	yes	up	mRNA
VIBR0546_10059	VIBR0546_10059	chemotaxis protein histidine kinase	0.161	−2.63168	5E-30	7.4E-28	yes	down	mRNA
VIBR0546_05339	trmD	tRNA (guanine-N(1)-)-methyltransferase	0.109	−3.19329	1.4E-29	2E-27	yes	down	mRNA

VIBR0546_06817	VIBR0546_06817	glycine betaine ABC transporter ATP-binding protein	0.118	−3.08686	8.8E-29	1.2E-26	yes	down	mRNA
VIBR0546_10014	VIBR0546_10014	response regulator	0.119	−3.06652	1.3E-28	1.7E-26	yes	down	mRNA
VIBR0546_11832	VIBR0546_11832	putative carbon-nitrogen hydrolase	18.78	4.23113	2.6E-28	3.4E-26	yes	up	mRNA
VIBR0546_02970	VIBR0546_02970	collagenase	0.1	−3.32356	5.2E-28	6.5E-26	yes	down	mRNA
VIBR0546_10069	VIBR0546_10069	hypothetical protein	0.103	−3.27406	5.6E-28	6.9E-26	yes	down	mRNA
VIBR0546_00515	VIBR0546_00515	periplasmic protein involved in polysaccharide export	0.083	−3.5953	9.2E-28	1.1E-25	yes	down	mRNA
VIBR0546_18616	VIBR0546_18616	hemolysin	11.07	3.46858	2.9E-27	3.3E-25	yes	up	mRNA
VIBR0546_00005	VIBR0546_00005	hypothetical protein	0.123	−3.02543	3.8E-27	4.3E-25	yes	down	mRNA
VIBR0546_06382	VIBR0546_06382	IcmF-related protein	0.13	−2.93795	7.5E-27	8.3E-25	yes	down	mRNA
VIBR0546_10019	VIBR0546_10019	putative anti-sigma F factor antagonist	0.085	−3.56193	7.8E-27	8.4E-25	yes	down	mRNA
VIBR0546_07567	VIBR0546_07567	hypothetical protein	0.145	−2.78214	2.2E-26	2.3E-24	yes	down	mRNA
VIBR0546_02001	VIBR0546_02001	hypothetical protein	11.372	3.50741	6E-26	6.1E-24	yes	up	mRNA
VIBR0546_16868	VIBR0546_16868	hypothetical protein	7.777	2.95918	8.6E-26	8.6E-24	yes	up	mRNA
VIBR0546_03370	VIBR0546_03370	LyrR family transcriptional regulator	7.166	2.84108	9E-26	8.8E-24	yes	up	mRNA
VIBR0546_17173	VIBR0546_17173	phosphonate ABC transporter%2C periplasmic phosphonate-binding protein	0.103	−3.28027	2.2E-25	2.1E-23	yes	down	mRNA
VIBR0546_01741	VIBR0546_01741	putative acetyltransferase	0.147	−2.76162	2.2E-25	2.1E-23	yes	down	mRNA
VIBR0546_10054	VIBR0546_10054	purine-binding chemotaxis protein CheW	0.12	−3.05989	2.7E-25	2.5E-23	yes	down	mRNA
VIBR0546_02800	VIBR0546_02800	glycine betaine/L-proline ABC transporter permease protein	0.043	−4.54283	3.5E-25	3.2E-23	yes	down	mRNA
VIBR0546_12982	VIBR0546_12982	putative multidrug resistance protein	0.107	−3.22495	1.4E-24	1.2E-22	yes	down	mRNA
VIBR0546_09267	VIBR0546_09267	phosphoribosylaminoimidazole carboxylase catalytic subunit	6.501	2.70056	5.6E-24	4.8E-22	yes	up	mRNA
VIBR0546_11827	VIBR0546_11827	putative oxidoreductase	12.758	3.67338	5.8E-24	4.9E-22	yes	up	mRNA
VIBR0546_10049	VIBR0546_10049	chemotaxis signal transduction protein	0.161	−2.63638	6.8E-24	5.7E-22	yes	down	mRNA
VIBR0546_00510	VIBR0546_00510	outer membrane protein	0.081	−3.63084	7.5E-24	6.1E-22	yes	down	mRNA
VIBR0546_11822	VIBR0546_11822	putative aldehyde dehydrogenase	10.58	3.40325	1.1E-23	8.8E-22	yes	up	mRNA
VIBR0546_00505	VIBR0546_00505	putative anti-sigma regulatory factor	0.053	−4.22554	1.7E-23	1.3E-21	yes	down	mRNA
VIBR0546_05304	VIBR0546_05304	membrane protein	0.184	−2.44149	4.3E-23	3.3E-21	yes	down	mRNA
VIBR0546_10039	VIBR0546_10039	chemotaxis protein methyltransferase CheR	0.074	−3.76611	5.6E-23	4.2E-21	yes	down	mRNA
VIBR0546_05084	VIBR0546_05084	putative DNA-binding protein	4.695	2.23101	7.2E-23	5.4E-21	yes	up	mRNA
VIBR0546_06502	VIBR0546_06502	saccharopine dehydrogenase	7.066	2.82084	8.6E-23	6.3E-21	yes	up	mRNA
VIBR0546_13820	secF	preprotein translocase subunit SecF	15.74	3.97634	9.7E-23	7E-21	yes	up	mRNA
VIBR0546_00340	VIBR0546_00340	formate dehydrogenase%2C cytochrome B556 subunit	0.135	−2.89224	2.4E-22	1.7E-20	yes	down	mRNA
VIBR0546_05872	VIBR0546_05872	hypothetical protein	0.178	−2.48927	3.5E-22	2.4E-20	yes	down	mRNA

VIBR0546_15866	VIBR0546_15866	SpoVR family protein	0.175	−2.51062	3.7E-22	2.5E-20	yes	down	mRNA
VIBR0546_13392	VIBR0546_13392	general secretion pathway protein H	0.094	−3.41299	1.2E-21	8.2E-20	yes	down	mRNA
VIBR0546_13412	VIBR0546_13412	general secretion pathway protein L	0.176	−2.50646	2.2E-21	1.4E-19	yes	down	mRNA
VIBR0546_05892	VIBR0546_05892	cytochrome c peroxidase	0.085	−3.55806	6.2E-21	4.1E-19	yes	down	mRNA
VIBR0546_18066	VIBR0546_18066	TPR repeat-containing protein	0.165	−2.60024	6.3E-21	4.1E-19	yes	down	mRNA
VIBR0546_15941	VIBR0546_15941	NADH dehydrogenase	4.221	2.07742	1E-20	6.4E-19	yes	up	mRNA
VIBR0546_07577	VIBR0546_07577	hypothetical protein	0.153	−2.70543	1.4E-20	8.7E-19	yes	down	mRNA
VIBR0546_18276	VIBR0546_18276	isocitrate lyase	10.198	3.35023	1.5E-20	9.3E-19	yes	up	mRNA
VIBR0546_00525	VIBR0546_00525	hypothetical protein	0.137	−2.86866	1.6E-20	9.5E-19	yes	down	mRNA
VIBR0546_06917	tnaA	tryptophanase/L-cysteine desulfhydrase%2C PLP-dependent	0.143	−2.80435	3.1E-20	1.8E-18	yes	down	mRNA
VIBR0546_15396	VIBR0546_15396	methyl-accepting chemotaxis protein	0.119	−3.06764	4.4E-20	2.6E-18	yes	down	mRNA
VIBR0546_05089	VIBR0546_05089	LysE/YggA family protein	10.983	3.45719	4.5E-20	2.6E-18	yes	up	mRNA
VIBR0546_00335	VIBR0546_00335	hypothetical protein	0.078	−3.68898	5.1E-20	3E-18	yes	down	mRNA
VIBR0546_00765	VIBR0546_00765	phosphoribosylaminoimidazole-succinocar-boxamide synthase	5.156	2.36629	7.4E-20	4.2E-18	yes	up	mRNA
VIBR0546_11617	VIBR0546_11617	polyhydroxyalkanoic acid synthase	0.129	−2.95163	9.2E-20	5.2E-18	yes	down	mRNA
VIBR0546_10334	VIBR0546_10334	N-acetylglucosamine-binding protein A	7.837	2.9703	1.1E-19	6.2E-18	yes	up	mRNA
VIBR0546_11732	VIBR0546_11732	diaminobutyrate--2-oxoglutarate aminotrans-ferase	0.049	−4.35356	2.9E-19	1.6E-17	yes	down	mRNA
VIBR0546_15811	VIBR0546_15811	histidine transport ATP-binding protein HisP	0.216	−2.21279	3.1E-19	1.7E-17	yes	down	mRNA
VIBR0546_11153	VIBR0546_11153	GTPase HflX	0.148	−2.75217	3.8E-19	2E-17	yes	down	mRNA
VIBR0546_15097	secY	preprotein translocase subunit SecY	0.175	−2.51379	4.8E-19	2.5E-17	yes	down	mRNA
VIBR0546_11847	VIBR0546_11847	4-aminobutyrate aminotransferase	7.046	2.81676	6.1E-19	3.2E-17	yes	up	mRNA
VIBR0546_05613	VIBR0546_05613	putative transcriptional regulator	4.383	2.13188	6.7E-19	3.5E-17	yes	up	mRNA
VIBR0546_13765	VIBR0546_13765	DNA polymerase III subunit epsilon	0.214	−2.22702	7.4E-19	3.8E-17	yes	down	mRNA
VIBR0546_16441	VIBR0546_16441	hypothetical protein	0.169	−2.56326	1.1E-18	5.3E-17	yes	down	mRNA
VIBR0546_18221	glpD	glycerol-3-phosphate dehydrogenase	0.05	−4.30874	2.4E-18	1.2E-16	yes	down	mRNA
VIBR0546_04579	VIBR0546_04579	hypothetical protein	8.504	3.0882	2.6E-18	1.3E-16	yes	up	mRNA
VIBR0546_05309	VIBR0546_05309	RNA polymerase sigma factor RpoS	0.183	−2.44737	3E-18	1.4E-16	yes	down	mRNA
VIBR0546_14235	VIBR0546_14235	hypothetical protein	4.569	2.19176	3.5E-18	1.7E-16	yes	up	mRNA
VIBR0546_06497	VIBR0546_06497	aminotransferase%2C class III/decarbox-ylase%2C group II	7.06	2.81968	5.3E-18	2.5E-16	yes	up	mRNA
VIBR0546_16863	VIBR0546_16863	hypothetical protein	6.804	2.76645	6.5E-18	3E-16	yes	up	mRNA
VIBR0546_02795	VIBR0546_02795	ATP-binding component of ABC transporter	0.147	−2.76605	7.6E-18	3.5E-16	yes	down	mRNA
VIBR0546_03575	VIBR0546_03575	permease	4.857	2.27995	8E-18	3.7E-16	yes	up	mRNA
VIBR0546_08149	VIBR0546_08149	putative glutathione S-transferase	5.543	2.47058	1.3E-17	5.7E-16	yes	up	mRNA

VIBR0546_12207	VIBR0546_12207	binding protein component of ABC transporter	4.207	2.07273	1.7E-17	7.6E-16	yes	up	mRNA
VIBR0546_10324	VIBR0546_10324	hypothetical protein	8.041	3.00739	1.7E-17	7.6E-16	yes	up	mRNA
VIBR0546_14025	VIBR0546_14025	Glyoxalase/bleomycin resistance protein/dioxygenase	10.036	3.32712	1.8E-17	7.8E-16	yes	up	mRNA
VIBR0546_10064	VIBR0546_10064	chemotaxis protein CheY	0.215	−2.21431	2.6E-17	1.1E-15	yes	down	mRNA
VIBR0546_07037	VIBR0546_07037	GlpM family protein	9.499	3.24773	3.7E-17	1.6E-15	yes	up	mRNA
VIBR0546_10309	VIBR0546_10309	cold shock protein CspE	0.179	−2.47896	1.8E-16	7.8E-15	yes	down	mRNA
VIBR0546_06822	VIBR0546_06822	glycine betaine/L-proline ABC transporter permease protein	0.183	−2.45078	1.9E-16	8E-15	yes	down	mRNA
VIBR0546_18196	VIBR0546_18196	aldo/keto reductase family oxidoreductase	6.283	2.65145	2.7E-16	1.1E-14	yes	up	mRNA
VIBR0546_01391	VIBR0546_01391	fructose-1%2C6-bisphosphatase	0.165	−2.60001	2.9E-16	1.2E-14	yes	down	mRNA
VIBR0546_09584	VIBR0546_09584	hypothetical protein	0.098	−3.3522	3.3E-16	1.3E-14	yes	down	mRNA
VIBR0546_05608	VIBR0546_05608	RNA polymerase sigma factor	4.189	2.06661	4.6E-16	1.8E-14	yes	up	mRNA
VIBR0546_05334	rimM	16S rRNA-processing protein RimM	0.225	−2.1503	5E-16	2E-14	yes	down	mRNA
VIBR0546_10099	VIBR0546_10099	chitodextrinase precursor	4.945	2.30605	6.6E-16	2.6E-14	yes	up	mRNA
VIBR0546_16126	VIBR0546_16126	LexA repressor	0.248	−2.01243	7.6E-16	3E-14	yes	down	mRNA
VIBR0546_17178	VIBR0546_17178	ABC transporter: ATP-binding protein	0.141	−2.8237	1.8E-15	6.8E-14	yes	down	mRNA
VIBR0546_11148	hfq	RNA-binding protein Hfq	0.184	−2.44533	1.8E-15	7.1E-14	yes	down	mRNA
VIBR0546_21430	VIBR0546_21430	inosine/uridine-preferring nucleoside hydrolase	0.141	−2.8309	2.1E-15	8.1E-14	yes	down	mRNA
VIBR0546_09919	VIBR0546_09919	putative transcriptional regulator	18.921	4.24195	3.1E-15	1.1E-13	yes	up	mRNA
VIBR0546_11622	VIBR0546_11622	hypothetical protein	0.192	−2.38075	3.5E-15	1.3E-13	yes	down	mRNA
VIBR0546_07562	VIBR0546_07562	ParA family protein	0.2	−2.32105	3.5E-15	1.3E-13	yes	down	mRNA
VIBR0546_06387	VIBR0546_06387	hypothetical protein	0.136	−2.87906	3.7E-15	1.4E-13	yes	down	mRNA
VIBR0546_15222	VIBR0546_15222	2-isopropylmalate synthase	4.124	2.04413	3.8E-15	1.4E-13	yes	up	mRNA
VIBR0546_17463	VIBR0546_17463	UDP-glucose 6-dehydrogenase	5.291	2.40347	5.3E-15	1.9E-13	yes	up	mRNA
VIBR0546_15092	rplO	50S ribosomal protein L15	0.236	−2.08299	5.7E-15	2E-13	yes	down	mRNA
VIBR0546_00520	VIBR0546_00520	putative capsular polysaccharide biosynthesis protein	0.087	−3.5175	1.3E-14	4.4E-13	yes	down	mRNA
VIBR0546_18051	VIBR0546_18051	Flp pilus assembly protein	0.234	−2.098	1.5E-14	5.3E-13	yes	down	mRNA
VIBR0546_20555	sucC	succinyl-CoA synthetase subunit beta	0.157	−2.66743	1.8E-14	5.9E-13	yes	down	mRNA
VIBR0546_08209	VIBR0546_08209	F0F1 ATP synthase subunit B	0.148	−2.75258	2.1E-14	7.1E-13	yes	down	mRNA
VIBR0546_21690	moaA	molybdenum cofactor biosynthesis protein A	4.266	2.093	2.6E-14	8.7E-13	yes	up	mRNA
VIBR0546_13137	VIBR0546_13137	putative protein disaggregation chaperone	0.174	−2.52259	2.9E-14	9.3E-13	yes	down	mRNA
VIBR0546_13387	VIBR0546_13387	general secretion pathway protein G	0.202	−2.30491	3.1E-14	1E-12	yes	down	mRNA
VIBR0546_06362	VIBR0546_06362	clpB protein	0.235	−2.09031	3.7E-14	1.2E-12	yes	down	mRNA
VIBR0546_13097	VIBR0546_13097	hypothetical protein	0.201	−2.31608	5E-14	1.6E-12	yes	down	mRNA

VIBR0546_06992	VIBR0546_06992	sodium/proline symporter	0.097	−3.36551	8.1E-14	2.5E-12	yes	down	mRNA
VIBR0546_12042	VIBR0546_12042	proton/glutamate symporter	5.165	2.36871	1.1E-13	3.6E-12	yes	up	mRNA
VIBR0546_19649	VIBR0546_19649	hypothetical protein	5.958	2.57476	1.2E-13	3.7E-12	yes	up	mRNA
VIBR0546_16431	VIBR0546_16431	hypothetical protein	0.206	−2.28259	1.4E-13	4.3E-12	yes	down	mRNA
VIBR0546_18061	VIBR0546_18061	Flp pilus assembly protein TadC	0.231	−2.11412	1.6E-13	4.7E-12	yes	down	mRNA
VIBR0546_09579	VIBR0546_09579	putative nitrogen regulatory protein P-II family protein	0.144	−2.80036	1.6E-13	4.7E-12	yes	down	mRNA
VIBR0546_18947	VIBR0546_18947	putative cytoplasmic protein	0.111	−3.17269	1.8E-13	5.3E-12	yes	down	mRNA
VIBR0546_13860	VIBR0546_13860	acetoacetyl-CoA synthetase	0.235	−2.08927	2E-13	5.8E-12	yes	down	mRNA
VIBR0546_03902	VIBR0546_03902	putative sodium/sulfate symporter	0.204	−2.29502	2E-13	6E-12	yes	down	mRNA
VIBR0546_18341	VIBR0546_18341	DNA-binding response regulator PhoB	5.607	2.48718	2.1E-13	6.2E-12	yes	up	mRNA
VIBR0546_15067	rpsH	30S ribosomal protein S8	0.236	−2.08361	2.5E-13	7.2E-12	yes	down	mRNA
VIBR0546_15167	VIBR0546_15167	hypothetical protein	4.323	2.11188	4.3E-13	1.2E-11	yes	up	mRNA
VIBR0546_08224	VIBR0546_08224	F0F1 ATP synthase subunit gamma	0.214	−2.22567	6.3E-13	1.8E-11	yes	down	mRNA
VIBR0546_11737	VIBR0546_11737	L-2%2C4-diaminobutyric acid acetyltransferase	0.044	−4.50322	6.4E-13	1.8E-11	yes	down	mRNA
VIBR0546_07317	VIBR0546_07317	putative aminotransferase	5.084	2.3459	1.3E-12	3.5E-11	yes	up	mRNA
VIBR0546_09574	VIBR0546_09574	hypothetical protein	0.156	−2.68433	1.3E-12	3.6E-11	yes	down	mRNA
VIBR0546_03100	VIBR0546_03100	hypothetical protein	0.249	−2.00725	1.9E-12	5E-11	yes	down	mRNA
VIBR0546_10134	VIBR0546_10134	hypothetical protein	0.159	−2.65612	2E-12	5.2E-11	yes	down	mRNA
VIBR0546_08134	VIBR0546_08134	oligopeptide ABC transporter%2C permease protein	0.151	−2.72439	2.1E-12	5.5E-11	yes	down	mRNA
VIBR0546_15052	rplX	50S ribosomal protein L24	0.231	−2.11466	3.6E-12	9.4E-11	yes	down	mRNA
VIBR0546_11235	VIBR0546_11235	hypothetical protein	0.201	−2.318	3.9E-12	1E-10	yes	down	mRNA
VIBR0546_13162	VIBR0546_13162	hypothetical protein	0.206	−2.27684	4.4E-12	1.1E-10	yes	down	mRNA
VIBR0546_02339	VIBR0546_02339	TRAP-type C4-dicarboxylate transport system small permease component	0.197	−2.34103	5.1E-12	1.3E-10	yes	down	mRNA
VIBR0546_08214	VIBR0546_08214	F0F1 ATP synthase subunit delta	0.213	−2.23337	5.4E-12	1.3E-10	yes	down	mRNA
VIBR0546_18546	VIBR0546_18546	GGDEF family protein	0.163	−2.61297	5.6E-12	1.4E-10	yes	down	mRNA
VIBR0546_00894	VIBR0546_00894	NptA protein	11.824	3.56367	5.9E-12	1.4E-10	yes	up	mRNA
VIBR0546_15736	VIBR0546_15736	response regulator	0.163	−2.61961	8.7E-12	2.1E-10	yes	down	mRNA
VIBR0546_09904	VIBR0546_09904	hypothetical protein	0.199	−2.33199	1E-11	2.5E-10	yes	down	mRNA
VIBR0546_17543	VIBR0546_17543	hypothetical protein	0.247	−2.01583	1.2E-11	2.7E-10	yes	down	mRNA
VIBR0546_09252	VIBR0546_09252	hypothetical protein	4.993	2.32001	1.7E-11	4E-10	yes	up	mRNA
VIBR0546_16436	VIBR0546_16436	hypothetical protein	0.225	−2.14915	1.8E-11	4E-10	yes	down	mRNA
VIBR0546_14555	VIBR0546_14555	putative deoxycytidylate deaminase	4.257	2.08975	2.6E-11	5.9E-10	yes	up	mRNA
VIBR0546_18431	VIBR0546_18431	sugar ABC transporter ATPase	0.127	−2.97338	2.7E-11	6.1E-10	yes	down	mRNA
VIBR0546_15062	VIBR0546_15062	30S ribosomal protein S14	0.221	−2.1811	2.7E-11	6.1E-10	yes	down	mRNA

VIBR0546_05099	VIBR0546_05099	oxidative stress defense protein	4.272	2.09479	3.5E-11	7.7E-10	yes	up	mRNA
VIBR0546_09484	VIBR0546_09484	outer membrane protein OmpA	0.25	−2.00021	4E-11	8.7E-10	yes	down	mRNA
VIBR0546_00345	VIBR0546_00345	formate dehydrogenase%2C iron-sulfur subunit	0.203	−2.29953	4.2E-11	9.1E-10	yes	down	mRNA
VIBR0546_19097	rplJ	50S ribosomal protein L10	0.25	−2.00063	4.5E-11	9.7E-10	yes	down	mRNA
VIBR0546_10714	glgA	glycogen synthase	0.148	−2.76067	4.8E-11	1E-09	yes	down	mRNA
VIBR0546_03857	VIBR0546_03857	FKBP-type peptidyl-prolyl cis-trans isomerase FklB	0.221	−2.17482	5.2E-11	1.1E-09	yes	down	mRNA
VIBR0546_21825	VIBR0546_21825	putative outer membrane protein	7.04	2.81568	6.8E-11	1.4E-09	yes	up	mRNA
VIBR0546_17293	VIBR0546_17293	NADH-dependent gamma-hydroxybutyrate dehydrogenase	9.795	3.29208	7.7E-11	1.6E-09	yes	up	mRNA
VIBR0546_00570	VIBR0546_00570	putative glycosyltransferase	0.079	−3.66573	8.1E-11	1.7E-09	yes	down	mRNA
VIBR0546_15591	VIBR0546_15591	glycine betaine ABC transporter substrate-binding protein	4.43	2.1473	9.6E-11	2E-09	yes	up	mRNA
VIBR0546_18056	VIBR0546_18056	Flp pilus assembly protein TadB	0.19	−2.39693	1E-10	2.1E-09	yes	down	mRNA
VIBR0546_01134	VIBR0546_01134	hypothetical protein	10.621	3.40882	1.4E-10	2.9E-09	yes	up	mRNA
VIBR0546_00840	VIBR0546_00840	peptidoglycan-binding LysM	0.088	−3.51175	1.6E-10	3.2E-09	yes	down	mRNA
VIBR0546_18747	VIBR0546_18747	NrfD protein	0.185	−2.43607	1.8E-10	3.5E-09	yes	down	mRNA
VIBR0546_10229	VIBR0546_10229	hypothetical protein	0.088	−3.51015	2E-10	4E-09	yes	down	mRNA
VIBR0546_03907	VIBR0546_03907	adenylylsulfate kinase	0.189	−2.40451	2.5E-10	4.8E-09	yes	down	mRNA
VIBR0546_02820	VIBR0546_02820	transcriptional regulator BetI	0.07	−3.83834	2.7E-10	5.2E-09	yes	down	mRNA
VIBR0546_09959	VIBR0546_09959	hypothetical protein	0.19	−2.39408	3.6E-10	6.9E-09	yes	down	mRNA
VIBR0546_10104	VIBR0546_10104	hypothetical protein	4.761	2.25141	4.7E-10	8.7E-09	yes	up	mRNA
VIBR0546_14667	VIBR0546_14667	hypothetical protein	5.525	2.46598	5.6E-10	1E-08	yes	up	mRNA
VIBR0546_11722	VIBR0546_11722	aspartate kinase	0.244	−2.03332	6E-10	1.1E-08	yes	down	mRNA
VIBR0546_16241	VIBR0546_16241	hypothetical protein	0.238	−2.06829	6.5E-10	1.2E-08	yes	down	mRNA
VIBR0546_08199	VIBR0546_08199	F0F1 ATP synthase subunit A	0.189	−2.4038	7.1E-10	1.3E-08	yes	down	mRNA
VIBR0546_18241	VIBR0546_18241	transcriptional regulator	4.155	2.05468	9E-10	1.6E-08	yes	up	mRNA
VIBR0546_13132	VIBR0546_13132	hypothetical protein	0.235	−2.08874	1.1E-09	1.9E-08	yes	down	mRNA
VIBR0546_00545	VIBR0546_00545	putative galactosyltransferase	0.132	−2.92507	1.2E-09	2E-08	yes	down	mRNA
VIBR0546_01746	VIBR0546_01746	putative fatty acid desaturase	0.194	−2.36948	1.7E-09	2.8E-08	yes	down	mRNA
VIBR0546_20585	VIBR0546_20585	hypothetical protein	6.242	2.64197	2.1E-09	3.5E-08	yes	up	mRNA
VIBR0546_06347	VIBR0546_06347	putative lipoprotein	0.217	−2.20237	2.2E-09	3.5E-08	yes	down	mRNA
VIBR0546_18131	VIBR0546_18131	DMT family permease	0.184	−2.43955	2.2E-09	3.5E-08	yes	down	mRNA
VIBR0546_17808	VIBR0546_17808	pirin-related protein	5.815	2.53985	2.4E-09	3.9E-08	yes	up	mRNA
VIBR0546_01251	dnaK	molecular chaperone DnaK	7.086	2.825	2.5E-09	4E-08	yes	up	mRNA
VIBR0546_00835	VIBR0546_00835	hypothetical protein	0.197	−2.34476	3.2E-09	4.9E-08	yes	down	mRNA
VIBR0546_21880	VIBR0546_21880	transcriptional regulator	4.279	2.09734	3.6E-09	5.6E-08	yes	up	mRNA

VIBR0546_00240	VIBR0546_00240	cytochrome c oxidase subunit CcoP	0.185	−2.43589	4.1E-09	6.3E-08	yes	down	mRNA
VIBR0546_07407	VIBR0546_07407	Multidrug resistance protein	0.214	−2.22512	4.2E-09	6.4E-08	yes	down	mRNA
VIBR0546_00979	VIBR0546_00979	phospho-2-dehydro-3-deoxyheptonate aldolase	4.108	2.03831	5.3E-09	7.9E-08	yes	up	mRNA
VIBR0546_12522	VIBR0546_12522	periplasmic substrate-binding transport protein%2C putative	0.246	−2.02161	6.4E-09	9.5E-08	yes	down	mRNA
VIBR0546_18636	VIBR0546_18636	methylmalonyl CoA epimerase	4.812	2.26658	7.3E-09	1.1E-07	yes	up	mRNA
VIBR0546_08204	VIBR0546_08204	F0F1 ATP synthase subunit C	0.158	−2.6618	7.7E-09	1.1E-07	yes	down	mRNA
VIBR0546_18732	nrfA	cytochrome c nitrite reductase subunit c552	0.171	−2.55093	8.3E-09	1.2E-07	yes	down	mRNA
VIBR0546_16131	VIBR0546_16131	O-methyltransferase-related protein	0.235	−2.08736	8.4E-09	1.2E-07	yes	down	mRNA
VIBR0546_18436	VIBR0546_18436	ABC transporter ATP-binding protein	0.212	−2.24094	9.6E-09	1.4E-07	yes	down	mRNA
VIBR0546_21485	VIBR0546_21485	methylmalonate-semialdehyde dehydrogenase	0.246	−2.02083	9.8E-09	1.4E-07	yes	down	mRNA
VIBR0546_00350	VIBR0546_00350	putative formate dehydrogenase large subunit	0.228	−2.13115	1.3E-08	1.8E-07	yes	down	mRNA
VIBR0546_08124	VIBR0546_08124	oligopeptide ABC transporter%2C periplasmic oligopeptide-binding protein	0.237	−2.07665	1.3E-08	1.8E-07	yes	down	mRNA
VIBR0546_13147	VIBR0546_13147	hypothetical protein	0.241	−2.05503	1.5E-08	2E-07	yes	down	mRNA
VIBR0546_13187	VIBR0546_13187	hypothetical protein	0.242	−2.04924	1.6E-08	2.2E-07	yes	down	mRNA
VIBR0546_04182	VIBR0546_04182	2-succinyl-6-hydroxy-2%2C4-cyclohexadiene-1-carboxylate synthase	0.224	−2.16081	1.8E-08	2.4E-07	yes	down	mRNA
VIBR0546_20560	VIBR0546_20560	succinyl-CoA synthetase subunit alpha	0.242	−2.04883	1.8E-08	2.4E-07	yes	down	mRNA
VIBR0546_08887	VIBR0546_08887	ABC transporter%2C periplasmic substrate-binding protein	5.108	2.35263	2E-08	2.6E-07	yes	up	mRNA
VIBR0546_13397	VIBR0546_13397	general secretion pathway protein I	0.136	−2.87955	2.2E-08	2.9E-07	yes	down	mRNA
VIBR0546_00230	VIBR0546_00230	cbb3-type cytochrome c oxidase subunit II	0.192	−2.37986	2.5E-08	3.3E-07	yes	down	mRNA
VIBR0546_12032	VIBR0546_12032	LysR family transcriptional regulator	4.024	2.00874	3.3E-08	4.1E-07	yes	up	mRNA
VIBR0546_19424	ndk	mulitfunctional nucleoside diphosphate kinase/apyrimidinic endonuclease	0.232	−2.1088	3.4E-08	4.2E-07	yes	down	mRNA
VIBR0546_15741	VIBR0546_15741	response regulator	0.186	−2.42777	4.2E-08	5.2E-07	yes	down	mRNA
VIBR0546_18456	VIBR0546_18456	sugar ABC transporter periplasmic protein	0.184	−2.44207	4.9E-08	5.9E-07	yes	down	mRNA
VIBR0546_08882	VIBR0546_08882	hypothetical protein	6.971	2.80137	5.4E-08	6.5E-07	yes	up	mRNA
VIBR0546_10094	VIBR0546_10094	hypothetical protein	4.06	2.02163	6.2E-08	7.3E-07	yes	up	mRNA
VIBR0546_06397	VIBR0546_06397	S-type Pyocin domain protein	0.145	−2.78189	6.5E-08	7.5E-07	yes	down	mRNA
VIBR0546_21425	VIBR0546_21425	protein kinase	0.227	−2.13728	7.2E-08	8.2E-07	yes	down	mRNA
VIBR0546_13142	VIBR0546_13142	hypothetical protein	0.219	−2.19217	1.1E-07	1.3E-06	yes	down	mRNA
VIBR0546_10599	VIBR0546_10599	hypothetical protein	4.489	2.1664	1.2E-07	1.4E-06	yes	up	mRNA
VIBR0546_21270	VIBR0546_21270	AcrB/AcrD/AcrF family transporter	0.204	−2.2942	1.3E-07	1.5E-06	yes	down	mRNA
VIBR0546_03155	VIBR0546_03155	hypothetical protein	10.057	3.33013	1.3E-07	1.5E-06	yes	up	mRNA

VIBR0546_15661	VIBR0546_15661	thiol:disulfide interchange protein DsbA	0.131	−2.93148	1.4E-07	1.5E-06	yes	down	mRNA
VIBR0546_06412	VIBR0546_06412	hypothetical protein	0.112	−3.16481	1.4E-07	1.6E-06	yes	down	mRNA
VIBR0546_18206	VIBR0546_18206	tRNA-binding protein	0.222	−2.17451	1.7E-07	1.9E-06	yes	down	mRNA
VIBR0546_09879	VIBR0546_09879	hypothetical protein	4.913	2.29667	1.8E-07	2E-06	yes	up	mRNA
VIBR0546_02680	VIBR0546_02680	hypothetical protein	4.267	2.09312	2E-07	2.1E-06	yes	up	mRNA
VIBR0546_20545	sucA	2-oxoglutarate dehydrogenase E1 component	0.205	−2.28418	2.4E-07	2.5E-06	yes	down	mRNA
VIBR0546_18742	VIBR0546_18742	hypothetical protein	0.238	−2.06834	3.1E-07	3.1E-06	yes	down	mRNA
VIBR0546_05344	rplS	50S ribosomal protein L19	0.225	−2.15378	3.1E-07	3.2E-06	yes	down	mRNA
VIBR0546_18752	VIBR0546_18752	cytochrome c biogenesis factor	0.235	−2.09194	3.2E-07	3.2E-06	yes	down	mRNA
VIBR0546_04177	VIBR0546_04177	dihydroxynaphthoic acid synthetase	0.242	−2.04571	3.3E-07	3.3E-06	yes	down	mRNA
VIBR0546_08580	VIBR0546_08580	hypothetical protein	0.13	−2.94497	4.3E-07	4.2E-06	yes	down	mRNA
VIBR0546_09544	VIBR0546_09544	heat shock protein 90	5.631	2.49338	5.7E-07	5.5E-06	yes	up	mRNA
VIBR0546_07137	VIBR0546_07137	phosphate ABC transporter%2C permease protein	5.154	2.36559	5.7E-07	5.5E-06	yes	up	mRNA
VIBR0546_20675	VIBR0546_20675	hypothetical protein	4.226	2.07942	6.6E-07	6.2E-06	yes	up	mRNA
VIBR0546_02780	VIBR0546_02780	hypothetical protein	4.45	2.15366	7.3E-07	6.8E-06	yes	up	mRNA
VIBR0546_03220	VIBR0546_03220	hypothetical protein	0.248	−2.00973	8.5E-07	7.8E-06	yes	down	mRNA
VIBR0546_06887	VIBR0546_06887	hypothetical protein	0.234	−2.09598	9E-07	8.2E-06	yes	down	mRNA
VIBR0546_07142	VIBR0546_07142	phosphate ABC transporter ATP-binding protein	4.636	2.21301	9.4E-07	8.5E-06	yes	up	mRNA
VIBR0546_00250	VIBR0546_00250	putative cation transport ATPase	0.205	−2.28652	1E-06	9E-06	yes	down	mRNA
VIBR0546_13705	VIBR0546_13705	electron transfer flavoprotein subunit beta	0.227	−2.13686	1.1E-06	9.9E-06	yes	down	mRNA
VIBR0546_13157	VIBR0546_13157	hypothetical protein	0.212	−2.24094	1.3E-06	1.2E-05	yes	down	mRNA
VIBR0546_15411	VIBR0546_15411	hypothetical protein	0.156	−2.67636	1.4E-06	1.2E-05	yes	down	mRNA
VIBR0546_08997	VIBR0546_08997	hypothetical protein	4.496	2.16879	1.5E-06	1.3E-05	yes	up	mRNA
VIBR0546_13407	VIBR0546_13407	general secretion pathway protein K	0.247	−2.01827	1.5E-06	1.3E-05	yes	down	mRNA
VIBR0546_18246	VIBR0546_18246	hypothetical protein	6.86	2.77826	1.5E-06	1.3E-05	yes	up	mRNA
VIBR0546_10339	VIBR0546_10339	hypothetical protein	4.038	2.0135	1.9E-06	1.6E-05	yes	up	mRNA
VIBR0546_11727	ectC	L-ectoine synthase	0.114	−3.1343	1.9E-06	1.6E-05	yes	down	mRNA
VIBR0546_14600	VIBR0546_14600	hypothetical protein	14.438	3.85185	2.8E-06	2.3E-05	yes	up	mRNA
VIBR0546_19362	VIBR0546_19362	DNA polymerase III subunit chi	7.127	2.83327	3.1E-06	2.5E-05	yes	up	mRNA
VIBR0546_18191	VIBR0546_18191	LysR family substrate binding transcriptional regulator	4.217	2.07624	3.4E-06	2.8E-05	yes	up	mRNA
VIBR0546_12242	VIBR0546_12242	hypothetical protein	4.032	2.0114	4.4E-06	3.5E-05	yes	up	mRNA
VIBR0546_15152	VIBR0546_15152	hypothetical protein	5.244	2.39076	5.4E-06	4.2E-05	yes	up	mRNA
VIBR0546_20580	VIBR0546_20580	hypothetical protein	6.084	2.60495	6.2E-06	4.7E-05	yes	up	mRNA
VIBR0546_02745	VIBR0546_02745	hypothetical protein	5.067	2.34118	6.3E-06	4.8E-05	yes	up	mRNA
VIBR0546_00565	VIBR0546_00565	acetyltransferase	0.111	−3.17114	1.1E-05	8.1E-05	yes	down	mRNA

VIBR0546_17493	VIBR0546_17493	LysR family transcriptional regulator	4.191	2.06724	1.3E-05	9.3E-05	yes	up	mRNA
VIBR0546_21410	VIBR0546_21410	hypothetical protein	0.212	−2.23994	2.1E-05	0.00014	yes	down	mRNA
VIBR0546_01986	VIBR0546_01986	hypothetical protein	5.19	2.3757	2.1E-05	0.00014	yes	up	mRNA
VIBR0546_17183	VIBR0546_17183	ABC transporter: transmembrane protein	0.24	−2.05613	2.4E-05	0.00015	yes	down	mRNA
VIBR0546_13700	VIBR0546_13700	acyl-CoA dehydrogenase%2C short-chain specific	0.249	−2.00429	2.5E-05	0.00016	yes	down	mRNA
VIBR0546_03230	VIBR0546_03230	lipoprotein-releasing system ATP-binding protein LolD	0.248	−2.01306	2.8E-05	0.00018	yes	down	mRNA
VIBR0546_05039	VIBR0546_05039	hypothetical protein	4.946	2.30621	2.9E-05	0.00018	yes	up	mRNA
VIBR0546_06407	VIBR0546_06407	LysM domain protein	0.081	−3.63249	3.5E-05	0.00022	yes	down	mRNA
VIBR0546_07132	VIBR0546_07132	phosphate ABC transporter%2C permease protein	4.326	2.11293	3.5E-05	0.00022	yes	up	mRNA
VIBR0546_21210	VIBR0546_21210	hypothetical protein	0.249	−2.00843	4.5E-05	0.00027	yes	down	mRNA
VIBR0546_11452	VIBR0546_11452	hypothetical protein	5.287	2.40256	6.9E-05	0.0004	yes	up	mRNA
VIBR0546_17928	VIBR0546_17928	ABC-type hydroxamate-dependent iron transport system%2C periplasmic component	0.207	−2.27482	7.8E-05	0.00044	yes	down	mRNA
VIBR0546_18446	VIBR0546_18446	binding-protein-dependent transport systems inner membrane component	0.16	−2.64329	8.2E-05	0.00046	yes	down	mRNA
VIBR0546_12797	VIBR0546_12797	hypothetical protein	0.213	−2.22861	9.7E-05	0.00054	yes	down	mRNA
VIBR0546_18136	VIBR0546_18136	putative oxidoreductase	0.237	−2.07466	0.00015	0.00082	yes	down	mRNA
VIBR0546_09102	VIBR0546_09102	hypothetical protein	0.082	−3.61138	0.00017	0.00089	yes	down	mRNA
VIBR0546_08972	VIBR0546_08972	small heat shock protein IbpA	16.875	4.07684	0.00024	0.0012	yes	up	mRNA
VIBR0546_18311	VIBR0546_18311	hypothetical protein	5.184	2.374	0.00025	0.00125	yes	up	mRNA
VIBR0546_02409	VIBR0546_02409	lactoylglutathione lyase	5.319	2.41105	0.00026	0.00131	yes	up	mRNA
VIBR0546_04032	VIBR0546_04032	tryptophan-specific transport protein	4.291	2.10127	0.00037	0.00175	yes	up	mRNA
VIBR0546_20083	VIBR0546_20083	hypothetical protein	4.32	2.11093	0.00047	0.00214	yes	up	mRNA
VIBR0546_19047	VIBR0546_19047	hypothetical protein	5.433	2.44188	0.00059	0.0026	yes	up	mRNA
VIBR0546_12737	VIBR0546_12737	hypothetical protein	7.46	2.89914	0.00081	0.00348	yes	up	mRNA
VIBR0546_03275	VIBR0546_03275	drug/metabolite transporter superfamily permease	4.292	2.10179	0.00169	0.00643	yes	up	mRNA
VIBR0546_20078	VIBR0546_20078	hypothetical protein	4.036	2.01282	0.00198	0.00735	yes	up	mRNA
VIBR0546_06207	VIBR0546_06207	metal dependent phosphohydrolase	10.191	3.34921	0.00198	0.00735	yes	up	mRNA
VIBR0546_19993	VIBR0546_19993	two component transcriptional regulator	4.729	2.2414	0.00208	0.00769	yes	up	mRNA
VIBR0546_00845	VIBR0546_00845	hypothetical protein	0.119	−3.06708	0.00255	0.00915	yes	down	mRNA
VIBR0546_13007	VIBR0546_13007	glutaredoxin	4.383	2.13204	0.00301	0.01046	yes	up	mRNA
VIBR0546_02640	VIBR0546_02640	hypothetical protein	7.998	2.99966	0.00334	0.01146	yes	up	mRNA
VIBR0546_18601	VIBR0546_18601	hypothetical protein	5.654	2.49933	0.00335	0.01146	yes	up	mRNA
VIBR0546_12262	VIBR0546_12262	hypothetical protein	4.824	2.27029	0.00359	0.01218	yes	up	mRNA

VIBR0546_17263	VIBR0546_17263	hypothetical protein	11.415	3.51287	0.0056	0.01789	yes	up	mRNA
VIBR0546_10234	VIBR0546_10234	hypothetical protein	0.127	−2.97856	0.00617	0.01942	yes	down	mRNA
VIBR0546_20955	VIBR0546_20955	hypothetical protein	16.219	4.0196	0.00678	0.02102	yes	up	mRNA
VIBR0546_17933	VIBR0546_17933	ABC-type hydroxamate-dependent iron transport system%2C ATPase component	0.196	−2.3545	0.00684	0.02118	yes	down	mRNA
VIBR0546_04589	VIBR0546_04589	hypothetical protein	0.155	−2.69344	0.00748	0.02263	yes	down	mRNA
VIBR0546_19973	VIBR0546_19973	hypothetical protein	16.746	4.0657	0.00824	0.02455	yes	up	mRNA
VIBR0546_21560	VIBR0546_21560	hypothetical protein	4.096	2.03421	0.00849	0.02515	yes	up	mRNA
VIBR0546_12272	VIBR0546_12272	hypothetical protein	22.809	4.51155	0.00967	0.02797	yes	up	mRNA
VIBR0546_11168	VIBR0546_11168	hypothetical protein	4.551	2.18633	0.01009	0.02907	yes	up	mRNA
VIBR0546_07889	VIBR0546_07889	nitrogen regulatory protein P-II	0.243	−2.03963	0.0101	0.02907	yes	down	mRNA
VIBR0546_00160	VIBR0546_00160	thiol-disulfide isomerase and thioredoxin	0.207	−2.26975	0.01065	0.03049	yes	down	mRNA
VIBR0546_12267	VIBR0546_12267	hypothetical protein	11.594	3.53532	0.0111	0.03155	yes	up	mRNA
VIBR0546_05822	VIBR0546_05822	hypothetical protein	7.381	2.8838	0.01161	0.03274	yes	up	mRNA
VIBR0546_18101	VIBR0546_18101	hypothetical protein	4.407	2.13975	0.01388	0.03797	yes	up	mRNA
VIBR0546_13062	VIBR0546_13062	ABC transporter transmembrane protein	5.617	2.48989	0.01633	0.04346	yes	up	mRNA
VIBR0546_19212	VIBR0546_19212	PTS system%2C fructose-specific IIA component	4.447	2.15298	0.01821	0.0476	yes	up	mRNA
VIBR0546_02645	VIBR0546_02645	hypothetical protein	5.125	2.35763	0.01828	0.04769	yes	up	mRNA
VIBR0546_01886	VIBR0546_01886	hypothetical protein	7.125	2.83282	0.0184	0.04793	yes	up	mRNA

Note: where the E stands for the times 10 to the power, for example, 1.8E-12 = 1.8×10^{-12}.

Table S3. Stress-related differentially expressed genes screened by *V. brasiliensis* cultured in 7% NaCl. (Screening criteria: Abs(log2FC) ≥ 1, $p < 0.05$).

Gene_id	Gene name	Gene description	FC(Nacl_7/CK)	Log2FC(Nacl_7/CK)	*p* Value	*p* Adjust	Significant	Regulate	Type
VIBR0546_07347	VIBR0546_07347	putative aldehyde dehydrogenase	0.033	−4.92584	2.3E-129	1E-125	yes	down	mRNA
VIBR0546_07217	VIBR0546_07217	outer membrane protein W	0.008	−6.88864	9.6E-120	2.1E-116	yes	down	mRNA
VIBR0546_00690	VIBR0546_00690	hypothetical protein	0.01	−6.67451	5.5E-111	7.9E-108	yes	down	mRNA
VIBR0546_17173	VIBR0546_17173	phosphonate ABC transporter%2C periplasmic phosphonate-binding protein	0.035	−4.83896	4.1E-110	4.4E-107	yes	down	mRNA
VIBR0546_04287	VIBR0546_04287	hypothetical protein	82.66	6.369114	2.2E-107	1.9E-104	yes	up	mRNA
VIBR0546_21070	VIBR0546_21070	hypothetical protein	0.026	−5.26912	1.4E-105	1E-102	yes	down	mRNA
VIBR0546_17523	VIBR0546_17523	putative polysaccharide deacetylase family protein	0.018	−5.83399	1.9E-103	1.2E-100	yes	down	mRNA
VIBR0546_09319	flgL	flagellar hook-associated protein FlgL	19.246	4.266512	7.2E-103	3.9E-100	yes	up	mRNA
VIBR0546_20755	VIBR0546_20755	cell division protein FtsQ	24.273	4.601306	7.3E-102	3.5E-99	yes	up	mRNA
VIBR0546_16166	VIBR0546_16166	putative arginine/ornithine antiporter	20.768	4.376299	3.8E-98	1.64E-95	yes	up	mRNA
VIBR0546_05688	VIBR0546_05688	DNA polymerase IV	38.546	5.268496	1.7E-97	6.66E-95	yes	up	mRNA
VIBR0546_04282	VIBR0546_04282	hypothetical protein	132.889	7.054079	2.49E-93	8.95E-91	yes	up	mRNA
VIBR0546_07057	VIBR0546_07057	hypothetical protein	0.057	−4.12563	7.49E-92	2.48E-89	yes	down	mRNA
VIBR0546_09324	flgK	flagellar hook-associated protein FlgK	14.957	3.902794	3.59E-90	1.11E-87	yes	up	mRNA
VIBR0546_05309	VIBR0546_05309	RNA polymerase sigma factor RpoS	0.028	−5.16285	7.74E-90	2.22E-87	yes	down	mRNA
VIBR0546_16136	VIBR0546_16136	DNA-damage-inducible protein F	25.298	4.660963	5.75E-89	1.55E-86	yes	up	mRNA
VIBR0546_18641	VIBR0546_18641	hypothetical protein	27.699	4.791759	1.42E-86	3.6E-84	yes	up	mRNA
VIBR0546_16131	VIBR0546_16131	O-methyltransferase-related protein	26.001	4.700473	4.27E-84	1.02E-81	yes	up	mRNA
VIBR0546_07322	VIBR0546_07322	extracellular solute-binding protein family 1	0.034	−4.88689	4.49E-83	1.02E-80	yes	down	mRNA
VIBR0546_05304	VIBR0546_05304	membrane protein	0.073	−3.77475	1.12E-82	2.42E-80	yes	down	mRNA
VIBR0546_10559	VIBR0546_10559	DNA recombination protein RmuC	14.519	3.859864	3.39E-82	6.97E-80	yes	up	mRNA
VIBR0546_07352	VIBR0546_07352	DNA-binding protein	0.044	−4.52217	6.78E-82	1.33E-79	yes	down	mRNA
VIBR0546_04292	VIBR0546_04292	error-prone DNA polymerase	39.804	5.314853	2.1E-81	3.95E-79	yes	up	mRNA
VIBR0546_21725	VIBR0546_21725	inosine monophosphate dehydrogenase-related protein	0.016	−6.00444	1.14E-80	2.05E-78	yes	down	mRNA
VIBR0546_05059	VIBR0546_05059	hypothetical protein	0.03	−5.06662	5.56E-80	9.6E-78	yes	down	mRNA
VIBR0546_04814	VIBR0546_04814	hypothetical protein	0.025	−5.33246	1.26E-77	2.09E-75	yes	down	mRNA
VIBR0546_07362	VIBR0546_07362	methylmalonate-semialdehyde dehydrogenase	0.071	−3.81913	1.06E-76	1.69E-74	yes	down	mRNA
VIBR0546_16708	VIBR0546_16708	hypothetical protein	91.36	6.513485	6.16E-76	9.48E-74	yes	up	mRNA
VIBR0546_05044	VIBR0546_05044	putative cytochrome c oxidase%2C subunit I	0.055	−4.18742	2.84E-75	4.22E-73	yes	down	mRNA
VIBR0546_17663	VIBR0546_17663	xanthine dehydrogenase accessory factor XdhC	0.039	−4.68347	3.6E-75	5.18E-73	yes	down	mRNA
VIBR0546_21810	VIBR0546_21810	DNA topoisomerase III	14.074	3.814962	4.24E-75	5.9E-73	yes	up	mRNA
VIBR0546_15406	VIBR0546_15406	hypothetical protein	0.048	−4.3836	5.04E-75	6.8E-73	yes	down	mRNA
VIBR0546_15856	VIBR0546_15856	PrkA family serine protein kinase	0.031	−5.02253	1.28E-71	1.67E-69	yes	down	mRNA

VIBR0546_17813	VIBR0546_17813	histidine kinase	0.03	−5.07774	1.44E-71	1.82E-69	yes	down	mRNA
VIBR0546_05892	VIBR0546_05892	cytochrome c peroxidase	0.011	−6.51214	3.09E-71	3.8E-69	yes	down	mRNA
VIBR0546_11267	VIBR0546_11267	CBS domain-containing protein	0.037	−4.76402	3.83E-71	4.59E-69	yes	down	mRNA
VIBR0546_11079	VIBR0546_11079	N5-glutamine S-adenosyl-L-methionine-dependent methyl-transferase	25.77	4.687638	7.25E-71	8.45E-69	yes	up	mRNA
VIBR0546_17533	VIBR0546_17533	allantoicase	0.019	−5.68475	8.68E-71	9.85E-69	yes	down	mRNA
VIBR0546_19397	recX	recombination regulator RecX	136.716	7.095034	4.28E-70	4.73E-68	yes	up	mRNA
VIBR0546_03125	VIBR0546_03125	hypothetical protein	0.048	−4.38082	1.17E-64	1.26E-62	yes	down	mRNA
VIBR0546_12002	VIBR0546_12002	hypothetical protein	0.024	−5.38339	1.94E-64	2.04E-62	yes	down	mRNA
VIBR0546_11477	VIBR0546_11477	TraF protein	0.021	−5.573	3.17E-64	3.26E-62	yes	down	mRNA
VIBR0546_09649	VIBR0546_09649	cysteine synthase A	0.043	−4.5374	6.16E-64	5.97E-62	yes	down	mRNA
VIBR0546_11622	VIBR0546_11622	hypothetical protein	0.011	−6.47309	6.21E-64	5.97E-62	yes	down	mRNA
VIBR0546_07819	VIBR0546_07819	ABC transporter%2C ATP-binding protein	20.161	4.333519	6.23E-64	5.97E-62	yes	up	mRNA
VIBR0546_10544	ubiB	putative ubiquinone biosynthesis protein UbiB	8.981	3.166796	1.24E-63	1.17E-61	yes	up	mRNA
VIBR0546_10779	VIBR0546_10779	Integration host factor%2C alpha subunit	0.095	−3.39812	4.06E-63	3.72E-61	yes	down	mRNA
VIBR0546_02394	VIBR0546_02394	hypothetical protein	15.481	3.952414	2.77E-62	2.49E-60	yes	up	mRNA
VIBR0546_15177	VIBR0546_15177	hypothetical protein	0.019	−5.73723	4.12E-62	3.62E-60	yes	down	mRNA
VIBR0546_01576	VIBR0546_01576	hypothetical protein	0.054	−4.21786	1.26E-61	1.08E-59	yes	down	mRNA
VIBR0546_08349	VIBR0546_08349	1%2C4-dihydroxy-2-naphthoate octaprenyltransferase	13.462	3.750858	1.31E-61	1.11E-59	yes	up	mRNA
VIBR0546_00625	VIBR0546_00625	hypothetical protein	10.515	3.394392	6.56E-61	5.44E-59	yes	up	mRNA
VIBR0546_02945	VIBR0546_02945	peptidase M14%2C carboxypeptidase A	0.088	−3.50856	1.31E-60	1.06E-58	yes	down	mRNA
VIBR0546_00365	VIBR0546_00365	iron-sulfur cluster-binding protein	14.798	3.88729	1.49E-60	1.19E-58	yes	up	mRNA
VIBR0546_11987	VIBR0546_11987	hypothetical protein	0.018	−5.7981	1.87E-60	1.47E-58	yes	down	mRNA
VIBR0546_04769	VIBR0546_04769	sigma factor RpoE regulatory protein RseC	20.889	4.384691	5.17E-60	3.98E-58	yes	up	mRNA
VIBR0546_13890	VIBR0546_13890	hypothetical protein	0.023	−5.465	1.01E-59	7.62E-58	yes	down	mRNA
VIBR0546_17528	VIBR0546_17528	hypothetical protein	0.025	−5.31045	1.26E-59	9.36E-58	yes	down	mRNA
VIBR0546_16506	VIBR0546_16506	group 1 glycosyl transferase	9.973	3.318059	3.29E-58	2.4E-56	yes	up	mRNA
VIBR0546_17668	VIBR0546_17668	xanthine dehydrogenase%2C molybdopterin binding subunit	0.034	−4.88976	5.11E-58	3.67E-56	yes	down	mRNA
VIBR0546_03892	VIBR0546_03892	sulfate adenylyltransferase subunit 2	0.088	−3.50648	3.98E-57	2.81E-55	yes	down	mRNA
VIBR0546_10064	VIBR0546_10064	chemotaxis protein CheY	0.029	−5.12534	2E-56	1.39E-54	yes	down	mRNA
VIBR0546_15866	VIBR0546_15866	SpoVR family protein	0.031	−5.00952	2.61E-56	1.79E-54	yes	down	mRNA
VIBR0546_11722	VIBR0546_11722	aspartate kinase	34.466	5.107098	2.88E-56	1.94E-54	yes	up	mRNA
VIBR0546_12567	VIBR0546_12567	zinc import ATP-binding protein ZnuC 2	17.184	4.103011	6.39E-56	4.24E-54	yes	up	mRNA
VIBR0546_08019	VIBR0546_08019	Mg2+ transporter mgtE	17.797	4.153526	2.78E-55	1.82E-53	yes	up	mRNA
VIBR0546_12852	VIBR0546_12852	histidine utilization repressor	23.5	4.554596	1.14E-54	7.31E-53	yes	up	mRNA
VIBR0546_01656	glgC	glucose-1-phosphate adenylyltransferase	0.041	−4.61369	1.57E-54	9.97E-53	yes	down	mRNA
VIBR0546_07397	VIBR0546_07397	GGDEF family protein	0.054	−4.19864	1.65E-54	1.03E-52	yes	down	mRNA
VIBR0546_00525	VIBR0546_00525	hypothetical protein	0.045	−4.47312	8.97E-54	5.52E-52	yes	down	mRNA

VIBR0546_09202	VIBR0546_09202	hypothetical protein	0.055	−4.19223	1.54E-53	9.35E-52	yes	down	mRNA
VIBR0546_17673	VIBR0546_17673	putative xanthine dehydrogenase%2C XdhA subunit	0.032	−4.94839	4.27E-53	2.56E-51	yes	down	mRNA
VIBR0546_09097	VIBR0546_09097	hypothetical protein	32.657	5.029333	7.92E-53	4.68E-51	yes	up	mRNA
VIBR0546_17538	VIBR0546_17538	ureidoglycolate hydrolase	0.025	−5.32986	1.02E-52	5.92E-51	yes	down	mRNA
VIBR0546_05433	VIBR0546_05433	hypothetical protein	7.327	2.873291	1.13E-52	6.47E-51	yes	up	mRNA
VIBR0546_17178	VIBR0546_17178	ABC transporter: ATP-binding protein	0.06	−4.05224	1.16E-52	6.59E-51	yes	down	mRNA
VIBR0546_05753	VIBR0546_05753	DNA-binding transcriptional regulator Crl	0.143	−2.80334	1.86E-52	1.04E-50	yes	down	mRNA
VIBR0546_11727	ectC	L-ectoine synthase	42.257	5.401134	3.25E-52	1.8E-50	yes	up	mRNA
VIBR0546_13367	VIBR0546_13367	general secretion pathway protein C	9.974	3.318118	4.99E-52	2.72E-50	yes	up	mRNA
VIBR0546_16486	VIBR0546_16486	hypothetical protein	14.197	3.827481	5.93E-52	3.2E-50	yes	up	mRNA
VIBR0546_08074	VIBR0546_08074	ribosomal-protein-alanine acetyltransferase	16.168	4.015037	7.14E-52	3.8E-50	yes	up	mRNA
VIBR0546_11567	VIBR0546_11567	putative AcrA/AcrE family protein	0.022	−5.51287	7.69E-52	4.04E-50	yes	down	mRNA
VIBR0546_12342	VIBR0546_12342	nicotinate phosphoribosyltransferase	12.854	3.684119	1.95E-51	1.01E-49	yes	up	mRNA
VIBR0546_01019	VIBR0546_01019	uncharacterized flavoprotein	0.061	−4.03029	4.01E-51	2.06E-49	yes	down	mRNA
VIBR0546_17693	VIBR0546_17693	Methyl-accepting chemotaxis protein	0.058	−4.11354	1.04E-50	5.26E-49	yes	down	mRNA
VIBR0546_03135	VIBR0546_03135	hypothetical protein	0.054	−4.21986	1.2E-50	6.04E-49	yes	down	mRNA
VIBR0546_19569	VIBR0546_19569	cold shock-like protein CspD	0.14	−2.83255	2.43E-50	1.2E-48	yes	down	mRNA
VIBR0546_11133	VIBR0546_11133	N-acetylmuramoyl-L-alanine amidase AmiB precursor	7.708	2.946323	4.45E-50	2.18E-48	yes	up	mRNA
VIBR0546_03420	VIBR0546_03420	putative outer membrane protein	0.005	−7.55814	5.75E-50	2.79E-48	yes	down	mRNA
VIBR0546_07824	VIBR0546_07824	inner membrane transport permease	34.917	5.125837	1.47E-49	7.05E-48	yes	up	mRNA
VIBR0546_13860	VIBR0546_13860	acetoacetyl-CoA synthetase	0.037	−4.74185	1.98E-49	9.39E-48	yes	down	mRNA
VIBR0546_12727	VIBR0546_12727	response regulator receiver modulated metal dependent phos-phohydrolase	0.068	−3.88389	2.54E-49	1.19E-47	yes	down	mRNA
VIBR0546_03480	VIBR0546_03480	phosphoglucomutase/phosphomannomutase alpha/beta/subu-nit	0.077	−3.69104	6.61E-49	3.06E-47	yes	down	mRNA
VIBR0546_05827	VIBR0546_05827	ATPase of the AAA+ class	0.053	−4.23127	9.2E-49	4.22E-47	yes	down	mRNA
VIBR0546_00125	VIBR0546_00125	hypothetical protein	0.083	−3.59535	1.81E-48	8.23E-47	yes	down	mRNA
VIBR0546_07357	VIBR0546_07357	beta alanine--pyruvate transaminase	0.059	−4.07642	5.75E-48	2.58E-46	yes	down	mRNA
VIBR0546_01226	VIBR0546_01226	recombination and repair protein	23.823	4.57428	1.31E-47	5.84E-46	yes	up	mRNA
VIBR0546_00510	VIBR0546_00510	outer membrane protein	0.038	−4.72644	7.75E-47	3.41E-45	yes	down	mRNA
VIBR0546_06842	VIBR0546_06842	coniferyl aldehyde dehydrogenase	0.129	−2.9538	1.06E-46	4.6E-45	yes	down	mRNA
VIBR0546_13217	prpB	2-methylisocitrate lyase	0.169	−2.56606	1.23E-46	5.3E-45	yes	down	mRNA
VIBR0546_02805	VIBR0546_02805	glycine betaine/L-proline ABC transporter glycine betaine/L-proline-binding protein	0.07	−3.84652	1.26E-46	5.37E-45	yes	down	mRNA
VIBR0546_15701	VIBR0546_15701	hypothetical protein	0.023	−5.41429	2.87E-46	1.21E-44	yes	down	mRNA
VIBR0546_16541	VIBR0546_16541	glucose dehydrogenase	0.08	−3.63523	3.06E-46	1.28E-44	yes	down	mRNA
VIBR0546_00090	yebU	rRNA (cytosine-C(5)-)-methyltransferase RsmF	7.51	2.908807	4.83E-46	2E-44	yes	up	mRNA
VIBR0546_04774	rseB	anti-sigma E factor	8.397	3.069896	1.37E-45	5.64E-44	yes	up	mRNA

VIBR0546_10739	VIBR0546_10739	hypothetical protein	0.076	−3.71963	1.71E-45	6.97E-44	yes	down	mRNA
VIBR0546_00919	VIBR0546_00919	bifunctional riboflavin kinase/FMN adenylyltransferase	6.776	2.760413	2.16E-45	8.72E-44	yes	up	mRNA
VIBR0546_01751	VIBR0546_01751	hypothetical protein	0.041	−4.62272	2.47E-45	9.86E-44	yes	down	mRNA
VIBR0546_04307	VIBR0546_04307	pyruvate kinase	0.076	−3.71298	4.76E-45	1.88E-43	yes	down	mRNA
VIBR0546_21055	VIBR0546_21055	8-amino-7-oxononanoate synthase	19.362	4.275131	1.12E-44	4.4E-43	yes	up	mRNA
VIBR0546_16648	VIBR0546_16648	DNA adenine methylase	13.463	3.750916	1.16E-44	4.51E-43	yes	up	mRNA
VIBR0546_16521	VIBR0546_16521	undecaprenyl-phosphate beta-N-acetyl-D-fucosa-minephosphotransferase	6.615	2.725752	1.74E-44	6.69E-43	yes	up	mRNA
VIBR0546_06162	VIBR0546_06162	hypothetical protein	0.026	−5.283	1.79E-44	6.83E-43	yes	down	mRNA
VIBR0546_06862	VIBR0546_06862	hypothetical protein	0.051	−4.28816	2.08E-44	7.86E-43	yes	down	mRNA
VIBR0546_11069	VIBR0546_11069	protein SirB1	9.04	3.176386	7.57E-44	2.84E-42	yes	up	mRNA
VIBR0546_17828	VIBR0546_17828	hypothetical protein	0.104	−3.26006	8.45E-44	3.14E-42	yes	down	mRNA
VIBR0546_17048	VIBR0546_17048	putative threonine efflux protein	26.152	4.708827	1.9E-43	7E-42	yes	up	mRNA
VIBR0546_02815	VIBR0546_02815	betaine aldehyde dehydrogenase	0.07	−3.83942	4.15E-43	1.52E-41	yes	down	mRNA
VIBR0546_21050	VIBR0546_21050	biotin synthase	12.649	3.660976	4.89E-43	1.77E-41	yes	up	mRNA
VIBR0546_05698	VIBR0546_05698	hypothetical protein	12.22	3.611206	2.55E-42	9.17E-41	yes	up	mRNA
VIBR0546_09694	lnt	apolipoprotein N-acyltransferase	10.181	3.347872	7.34E-42	2.62E-40	yes	up	mRNA
VIBR0546_04764	VIBR0546_04764	GTP-binding protein LepA	7.076	2.823036	1.06E-41	3.73E-40	yes	up	mRNA
VIBR0546_06917	tnaA	tryptophanase/L-cysteine desulfhydrase%2C PLP-dependent	0.065	−3.93606	2.03E-41	7.12E-40	yes	down	mRNA
VIBR0546_19499	VIBR0546_19499	putative S-adenosyl-L-methionine-dependent methyltransfer-ase	7.581	2.922409	2.93E-41	1.02E-39	yes	up	mRNA
VIBR0546_14125	rplT	50S ribosomal protein L20	0.081	−3.62662	3.23E-41	1.11E-39	yes	down	mRNA
VIBR0546_17168	VIBR0546_17168	glycerophosphoryl diester phosphodiesterase	0.076	−3.70859	4.2E-41	1.44E-39	yes	down	mRNA
VIBR0546_10079	VIBR0546_10079	transcriptional regulator	0.117	−3.09452	5.53E-41	1.88E-39	yes	down	mRNA
VIBR0546_11472	VIBR0546_11472	ABC-type sugar transport system%2C periplasmic compo-nent	0.133	−2.90944	6.89E-41	2.32E-39	yes	down	mRNA
VIBR0546_10929	VIBR0546_10929	electron transport complex protein RsxA	6.282	2.651201	6.98E-41	2.33E-39	yes	up	mRNA
VIBR0546_11542	VIBR0546_11542	aromatic amino acid aminotransferase	0.123	−3.02032	8.95E-41	2.97E-39	yes	down	mRNA
VIBR0546_10104	VIBR0546_10104	hypothetical protein	0.027	−5.20852	9.85E-41	3.24E-39	yes	down	mRNA
VIBR0546_17853	VIBR0546_17853	signal transduction protein	0.086	−3.54634	1.52E-40	4.95E-39	yes	down	mRNA
VIBR0546_09894	VIBR0546_09894	signal transduction protein	0.038	−4.72823	1.53E-40	4.95E-39	yes	down	mRNA
VIBR0546_07602	VIBR0546_07602	cytochrome c-type biogenesis protein CcmE	8.517	3.090323	2.6E-40	8.38E-39	yes	up	mRNA
VIBR0546_12147	VIBR0546_12147	antioxidant putative	0.074	−3.76167	2.88E-40	9.2E-39	yes	down	mRNA
VIBR0546_12477	VIBR0546_12477	hypothetical protein	10.161	3.344993	3.5E-40	1.11E-38	yes	up	mRNA
VIBR0546_15861	VIBR0546_15861	hypothetical protein	0.05	−4.32021	4.7E-40	1.48E-38	yes	down	mRNA
VIBR0546_16576	pheS	phenylalanyl-tRNA synthetase subunit alpha	4.744	2.246234	8.61E-40	2.69E-38	yes	up	mRNA
VIBR0546_18982	VIBR0546_18982	ATPase	5.111	2.353572	3.07E-39	9.52E-38	yes	up	mRNA
VIBR0546_19983	VIBR0546_19983	sensory box sensor histidine kinase/response regulator VieS	9.616	3.265424	5.46E-39	1.68E-37	yes	up	mRNA

VIBR0546_14515	VIBR0546_14515	glutamine amidotransferase	0.167	−2.58501	8.06E-39	2.46E-37	yes	down	mRNA
VIBR0546_01079	VIBR0546_01079	YrbK protein	7.56	2.91843	8.71E-39	2.65E-37	yes	up	mRNA
VIBR0546_11347	VIBR0546_11347	UDP-glucose 4-epimerase	0.107	−3.2308	1.24E-38	3.73E-37	yes	down	mRNA
VIBR0546_17543	VIBR0546_17543	hypothetical protein	0.04	−4.65544	1.76E-38	5.27E-37	yes	down	mRNA
VIBR0546_03680	VIBR0546_03680	hypothetical protein	0.046	−4.43653	1.83E-38	5.43E-37	yes	down	mRNA
VIBR0546_19392	recA	recombinase A	7.875	2.977203	2.15E-38	6.35E-37	yes	up	mRNA
VIBR0546_20333	VIBR0546_20333	Hcp protein	0.071	−3.81703	2.21E-38	6.5E-37	yes	down	mRNA
VIBR0546_14000	VIBR0546_14000	hypothetical protein	0.043	−4.52951	2.87E-38	8.37E-37	yes	down	mRNA
VIBR0546_09684	VIBR0546_09684	metal-binding heat shock protein	10.273	3.360748	3.62E-38	1.05E-36	yes	up	mRNA
VIBR0546_15341	VIBR0546_15341	para-aminobenzoate synthase component I	10.376	3.375175	3.98E-38	1.14E-36	yes	up	mRNA
VIBR0546_11084	prfA	peptide chain release factor 1	4.655	2.21891	4.35E-38	1.24E-36	yes	up	mRNA
VIBR0546_16236	VIBR0546_16236	hypothetical protein	0.198	−2.33309	4.75E-38	1.35E-36	yes	down	mRNA
VIBR0546_17503	VIBR0546_17503	hydroxydechloroatrazine ethylaminohydrolase	0.097	−3.35942	9.33E-38	2.63E-36	yes	down	mRNA
VIBR0546_09117	VIBR0546_09117	hypothetical protein	10.622	3.409047	9.64E-38	2.7E-36	yes	up	mRNA
VIBR0546_03030	VIBR0546_03030	DNA and RNA helicase	0.148	−2.75303	1.11E-37	3.08E-36	yes	down	mRNA
VIBR0546_00175	VIBR0546_00175	ribosome modulation factor	0.08	−3.63712	1.12E-37	3.11E-36	yes	down	mRNA
VIBR0546_02800	VIBR0546_02800	glycine betaine/L-proline ABC transporter permease protein	0.059	−4.07721	1.28E-37	3.52E-36	yes	down	mRNA
VIBR0546_07587	VIBR0546_07587	heme exporter protein B	15.489	3.953188	1.45E-37	3.95E-36	yes	up	mRNA
VIBR0546_13047	VIBR0546_13047	GGDEF family protein	10.361	3.373136	1.66E-37	4.5E-36	yes	up	mRNA
VIBR0546_10069	VIBR0546_10069	hypothetical protein	0.051	−4.30235	1.76E-37	4.75E-36	yes	down	mRNA
VIBR0546_01646	VIBR0546_01646	PTS system fructose-specific IIBC component	0.048	−4.39263	1.86E-37	4.99E-36	yes	down	mRNA
VIBR0546_13222	VIBR0546_13222	methylcitrate synthase	0.112	−3.15879	2.52E-37	6.72E-36	yes	down	mRNA
VIBR0546_20323	VIBR0546_20323	outer membrane protein	0.062	−4.00714	3.32E-37	8.77E-36	yes	down	mRNA
VIBR0546_04017	VIBR0546_04017	bacterioferritin	0.137	−2.86388	3.44E-37	9.04E-36	yes	down	mRNA
VIBR0546_12077	VIBR0546_12077	hypothetical protein	0.064	−3.96179	4.02E-37	1.05E-35	yes	down	mRNA
VIBR0546_13945	VIBR0546_13945	putative periplasmic solute-binding protein	0.031	−5.01487	4.18E-37	1.09E-35	yes	down	mRNA
VIBR0546_00770	VIBR0546_00770	hypothetical protein	0.043	−4.54053	4.34E-37	1.12E-35	yes	down	mRNA
VIBR0546_16491	VIBR0546_16491	hypothetical protein	4.955	2.308859	7.15E-37	1.84E-35	yes	up	mRNA
VIBR0546_02169	VIBR0546_02169	hypothetical protein	0.059	−4.09422	8.7E-37	2.22E-35	yes	down	mRNA
VIBR0546_13770	VIBR0546_13770	hypothetical protein	0.061	−4.02862	8.81E-37	2.24E-35	yes	down	mRNA
VIBR0546_20750	ftsA	cell division protein FtsA	5.729	2.518183	1.1E-36	2.78E-35	yes	up	mRNA
VIBR0546_11697	VIBR0546_11697	ATP-dependent Clp protease proteolytic subunit	0.055	−4.19079	1.34E-36	3.35E-35	yes	down	mRNA
VIBR0546_05034	VIBR0546_05034	endonuclease I precursor	13.493	3.754117	1.91E-36	4.75E-35	yes	up	mRNA
VIBR0546_04192	VIBR0546_04192	menaquinone-specific isochorismate synthase	7.875	2.977324	1.99E-36	4.94E-35	yes	up	mRNA
VIBR0546_02940	VIBR0546_02940	L-serine dehydratase 1	0.158	−2.65826	2.1E-36	5.16E-35	yes	down	mRNA
VIBR0546_12812	VIBR0546_12812	hypothetical protein	15.685	3.971348	2.75E-36	6.73E-35	yes	up	mRNA
VIBR0546_00515	VIBR0546_00515	periplasmic protein involved in polysaccharide export	0.065	−3.94145	3.03E-36	7.39E-35	yes	down	mRNA
VIBR0546_14992	rpsJ	30S ribosomal protein S10	5.534	2.468411	4.42E-36	1.07E-34	yes	up	mRNA

VIBR0546_21150	VIBR0546_21150	Na+/H+ antiporter	9.496	3.247267	4.47E-36	1.08E-34	yes	up	mRNA
VIBR0546_00505	VIBR0546_00505	putative anti-sigma regulatory factor	0.03	−5.06565	5.08E-36	1.22E-34	yes	down	mRNA
VIBR0546_16081	VIBR0546_16081	cell division protein FtsX	8.629	3.109257	5.5E-36	1.31E-34	yes	up	mRNA
VIBR0546_01311	VIBR0546_01311	oxidoreductase	0.242	−2.0445	5.99E-36	1.42E-34	yes	down	mRNA
VIBR0546_08857	VIBR0546_08857	hypothetical protein	0.085	−3.56463	6.05E-36	1.43E-34	yes	down	mRNA
VIBR0546_08842	VIBR0546_08842	response regulator	0.087	−3.52708	7.23E-36	1.69E-34	yes	down	mRNA
VIBR0546_13487	VIBR0546_13487	tRNA guanosine-2'-O-methyltransferase	18.584	4.21598	8.5E-36	1.98E-34	yes	up	mRNA
VIBR0546_02810	VIBR0546_02810	choline dehydrogenase	0.081	−3.62887	1.08E-35	2.51E-34	yes	down	mRNA
VIBR0546_08359	VIBR0546_08359	multidrug efflux pump	8.722	3.124578	1.37E-35	3.16E-34	yes	up	mRNA
VIBR0546_03165	VIBR0546_03165	hypothetical protein	0.067	−3.89127	1.39E-35	3.19E-34	yes	down	mRNA
VIBR0546_15466	VIBR0546_15466	SanA protein	9.413	3.234675	1.43E-35	3.27E-34	yes	up	mRNA
VIBR0546_15806	VIBR0546_15806	hypothetical protein	0.115	−3.12059	2.02E-35	4.59E-34	yes	down	mRNA
VIBR0546_04614	VIBR0546_04614	ribonuclease activity regulator protein RraA	0.142	−2.81829	2.28E-35	5.14E-34	yes	down	mRNA
VIBR0546_01956	VIBR0546_01956	hypothetical protein	0.062	−4.01614	2.66E-35	5.97E-34	yes	down	mRNA
VIBR0546_00530	VIBR0546_00530	Signal transduction histidine kinase	0.172	−2.54265	2.74E-35	6.12E-34	yes	down	mRNA
VIBR0546_07312	VIBR0546_07312	glutamine synthetase	0.094	−3.40945	3.74E-35	8.3E-34	yes	down	mRNA
VIBR0546_07112	VIBR0546_07112	outer membrane lipoprotein	0.056	−4.14982	4.56E-35	1.01E-33	yes	down	mRNA
VIBR0546_00050	VIBR0546_00050	amino acid ABC transporter periplasmic amino acid-binding protein	0.073	−3.78239	4.63E-35	1.02E-33	yes	down	mRNA
VIBR0546_05414	VIBR0546_05414	ssDNA exonuclease RecJ	8.694	3.120008	6.42E-35	1.41E-33	yes	up	mRNA
VIBR0546_16481	VIBR0546_16481	glycosyltransferase	8.813	3.139672	6.85E-35	1.49E-33	yes	up	mRNA
VIBR0546_05254	VIBR0546_05254	putative glycerol-3-phosphate acyltransferase PlsY	8.396	3.069629	7.59E-35	1.64E-33	yes	up	mRNA
VIBR0546_05568	VIBR0546_05568	hypothetical protein	7.649	2.935321	1.29E-34	2.79E-33	yes	up	mRNA
VIBR0546_12817	VIBR0546_12817	hypothetical protein	0.085	−3.56229	2.91E-34	6.24E-33	yes	down	mRNA
VIBR0546_17138	VIBR0546_17138	methyl-accepting chemotaxis sensory transducer	0.035	−4.8205	3E-34	6.41E-33	yes	down	mRNA
VIBR0546_19963	VIBR0546_19963	methyl-accepting chemotaxis protein	17.527	4.131534	3.52E-34	7.48E-33	yes	up	mRNA
VIBR0546_06852	VIBR0546_06852	oxidoreductase	0.079	−3.65623	4E-34	8.46E-33	yes	down	mRNA
VIBR0546_16953	VIBR0546_16953	glyoxylate carboligase	0.049	−4.34012	4.47E-34	9.41E-33	yes	down	mRNA
VIBR0546_08009	VIBR0546_08009	membrane protein	7.836	2.970056	7.57E-34	1.58E-32	yes	up	mRNA
VIBR0546_19978	VIBR0546_19978	histidine kinase	21.414	4.420462	8.31E-34	1.73E-32	yes	up	mRNA
VIBR0546_03580	VIBR0546_03580	Small-conductance mechanosensitive channel	0.134	−2.89917	1.02E-33	2.12E-32	yes	down	mRNA
VIBR0546_04092	VIBR0546_04092	putative lipoprotein	0.193	−2.37038	1.14E-33	2.35E-32	yes	down	mRNA
VIBR0546_04869	VIBR0546_04869	hypothetical protein	13.565	3.761795	1.47E-33	3.02E-32	yes	up	mRNA
VIBR0546_04824	VIBR0546_04824	2-octaprenyl-6-methoxyphenyl hydroxylase	5.296	2.404794	1.86E-33	3.8E-32	yes	up	mRNA
VIBR0546_00535	VIBR0546_00535	putative LuxO repressor protein	0.071	−3.81277	1.93E-33	3.92E-32	yes	down	mRNA
VIBR0546_10549	VIBR0546_10549	hypothetical protein	10.025	3.325493	2.08E-33	4.2E-32	yes	up	mRNA
VIBR0546_10179	VIBR0546_10179	hypothetical protein	23.337	4.544572	2.1E-33	4.24E-32	yes	up	mRNA
VIBR0546_19449	hscB	co-chaperone HscB	8.775	3.13347	2.34E-33	4.68E-32	yes	up	mRNA

VIBR0546_05912	VIBR0546_05912	zinc protease	0.121	−3.04365	2.68E-33	5.35E-32	yes	down	mRNA
VIBR0546_20905	VIBR0546_20905	cyclic 3'%2C5'-adenosine monophosphate phosphodiesterase	5.508	2.461494	3.17E-33	6.3E-32	yes	up	mRNA
VIBR0546_14105	VIBR0546_14105	putative membrane protein	0.082	−3.60157	3.35E-33	6.62E-32	yes	down	mRNA
VIBR0546_14510	VIBR0546_14510	putative transporter	11.421	3.513609	3.74E-33	7.36E-32	yes	up	mRNA
VIBR0546_16788	VIBR0546_16788	hypothetical protein	0.101	−3.30782	4.69E-33	9.19E-32	yes	down	mRNA
VIBR0546_20795	VIBR0546_20795	peptidoglycan synthetase ftsI precursor	5.718	2.515612	5.07E-33	9.9E-32	yes	up	mRNA
VIBR0546_05987	VIBR0546_05987	ABC transporter: substrate binding protein precursor	0.109	−3.20214	5.47E-33	1.06E-31	yes	down	mRNA
VIBR0546_16386	VIBR0546_16386	hypothetical protein	5.451	2.4465	6.27E-33	1.21E-31	yes	up	mRNA
VIBR0546_13755	VIBR0546_13755	formate--tetrahydrofolate ligase	0.108	−3.21426	8.99E-33	1.73E-31	yes	down	mRNA
VIBR0546_13975	VIBR0546_13975	isochorismatase hydrolase	0.136	−2.8757	1.2E-32	2.3E-31	yes	down	mRNA
VIBR0546_13670	VIBR0546_13670	histidine kinase/response regulator hybrid protein	0.123	−3.02019	1.57E-32	2.99E-31	yes	down	mRNA
VIBR0546_21880	VIBR0546_21880	transcriptional regulator	16.354	4.031582	1.81E-32	3.44E-31	yes	up	mRNA
VIBR0546_13880	VIBR0546_13880	putative acetyltransferase	0.105	−3.25476	1.91E-32	3.6E-31	yes	down	mRNA
VIBR0546_16171	VIBR0546_16171	hypothetical protein	6.401	2.678318	3.44E-32	6.49E-31	yes	up	mRNA
VIBR0546_14570	VIBR0546_14570	hypothetical protein	0.08	−3.63798	3.71E-32	6.96E-31	yes	down	mRNA
VIBR0546_18456	VIBR0546_18456	sugar ABC transporter periplasmic protein	0.089	−3.48409	4.76E-32	8.89E-31	yes	down	mRNA
VIBR0546_16511	VIBR0546_16511	glycosyl transferase%2C group 1 family protein	5.004	2.323221	5.09E-32	9.45E-31	yes	up	mRNA
VIBR0546_01064	VIBR0546_01064	Ca2+/Na+ antiporter	10.1	3.336316	5.15E-32	9.54E-31	yes	up	mRNA
VIBR0546_13247	VIBR0546_13247	MutT/nudix family protein	7.624	2.930514	5.29E-32	9.75E-31	yes	up	mRNA
VIBR0546_04457	VIBR0546_04457	phage integrase family protein	13.005	3.701045	7.85E-32	1.44E-30	yes	up	mRNA
VIBR0546_01741	VIBR0546_01741	putative acetyltransferase	0.104	−3.26507	8.59E-32	1.57E-30	yes	down	mRNA
VIBR0546_14772	VIBR0546_14772	acetyl-CoA synthetase	0.13	−2.93868	1.11E-31	2.02E-30	yes	down	mRNA
VIBR0546_02274	ihfB	integration host factor subunit beta	0.131	−2.93696	1.15E-31	2.08E-30	yes	down	mRNA
VIBR0546_07092	VIBR0546_07092	hypothetical protein	0.123	−3.02531	1.47E-31	2.65E-30	yes	down	mRNA
VIBR0546_13452	VIBR0546_13452	hypothetical protein	0.125	−3.0001	1.7E-31	3.05E-30	yes	down	mRNA
VIBR0546_08560	ruvA	Holliday junction DNA helicase RuvA	8.819	3.140584	1.77E-31	3.17E-30	yes	up	mRNA
VIBR0546_18727	VIBR0546_18727	putative Cytochrome c biogenesis factor	8.308	3.054534	1.8E-31	3.2E-30	yes	up	mRNA
VIBR0546_01916	VIBR0546_01916	putative transcription regulator protein	0.098	−3.34683	2E-31	3.55E-30	yes	down	mRNA
VIBR0546_11732	VIBR0546_11732	diaminobutyrate--2-oxoglutarate aminotransferase	22.739	4.507067	2.45E-31	4.33E-30	yes	up	mRNA
VIBR0546_19327	VIBR0546_19327	PhoH family protein	0.146	−2.77556	2.69E-31	4.73E-30	yes	down	mRNA
VIBR0546_12947	VIBR0546_12947	fructose-2%2C6-bisphosphatase	7.212	2.85035	2.99E-31	5.24E-30	yes	up	mRNA
VIBR0546_12887	VIBR0546_12887	cytotoxin%2C cytolysin precursor VvhA	0.048	−4.38052	3.45E-31	6.03E-30	yes	down	mRNA
VIBR0546_11847	VIBR0546_11847	4-aminobutyrate aminotransferase	0.118	−3.07796	4.04E-31	7.02E-30	yes	down	mRNA
VIBR0546_12607	VIBR0546_12607	cell wall endopeptidase family M23/M37	15.73	3.97545	4.27E-31	7.39E-30	yes	up	mRNA
VIBR0546_06722	VIBR0546_06722	putative membrane protein	0.111	−3.17313	5.09E-31	8.77E-30	yes	down	mRNA
VIBR0546_10164	VIBR0546_10164	maltodextrin transport system permease MalC	13.458	3.750437	5.31E-31	9.12E-30	yes	up	mRNA
VIBR0546_17053	VIBR0546_17053	AraC family transcriptional regulator	8.266	3.047125	8.79E-31	1.5E-29	yes	up	mRNA
VIBR0546_01411	VIBR0546_01411	outer membrane protein	4.147	2.052029	1E-30	1.71E-29	yes	up	mRNA

VIBR0546_20870	VIBR0546_20870	hypothetical protein	5.238	2.389118	1.11E-30	1.89E-29	yes	up	mRNA
VIBR0546_10184	VIBR0546_10184	UDP-3-O-[3-hydroxymyristoyl] glucosamine N-acyltransfer-ase	19.37	4.275769	1.49E-30	2.51E-29	yes	up	mRNA
VIBR0546_07637	VIBR0546_07637	hypothetical protein	13.707	3.776885	1.6E-30	2.7E-29	yes	up	mRNA
VIBR0546_09634	ligA	NAD-dependent DNA ligase LigA	4.593	2.1993	1.71E-30	2.87E-29	yes	up	mRNA
VIBR0546_03465	VIBR0546_03465	hypothetical protein	12.872	3.686208	1.76E-30	2.95E-29	yes	up	mRNA
VIBR0546_00520	VIBR0546_00520	putative capsular polysaccharide biosynthesis protein	0.033	−4.90634	5.08E-30	8.45E-29	yes	down	mRNA
VIBR0546_10084	VIBR0546_10084	ParA family protein	0.118	−3.07895	5.82E-30	9.66E-29	yes	down	mRNA
VIBR0546_18066	VIBR0546_18066	TPR repeat-containing protein	0.099	−3.33159	7.87E-30	1.3E-28	yes	down	mRNA
VIBR0546_13815	VIBR0546_13815	hypothetical protein	0.055	−4.18985	1.66E-29	2.72E-28	yes	down	mRNA
VIBR0546_05084	VIBR0546_05084	putative DNA-binding protein	0.184	−2.44249	1.73E-29	2.84E-28	yes	down	mRNA
VIBR0546_14200	VIBR0546_14200	hypothetical protein	0.122	−3.03874	1.91E-29	3.12E-28	yes	down	mRNA
VIBR0546_13660	VIBR0546_13660	hypothetical protein	0.166	−2.59197	2.08E-29	3.38E-28	yes	down	mRNA
VIBR0546_04759	VIBR0546_04759	signal peptidase I	8.028	3.005035	2.38E-29	3.86E-28	yes	up	mRNA
VIBR0546_16446	VIBR0546_16446	heteropolysaccharide repeat-containing protein	7.018	2.81114	2.4E-29	3.88E-28	yes	up	mRNA
VIBR0546_15296	VIBR0546_15296	GGDEF family protein	0.088	−3.51304	2.53E-29	4.07E-28	yes	down	mRNA
VIBR0546_12847	VIBR0546_12847	imidazolonepropionase	11.361	3.505985	2.56E-29	4.1E-28	yes	up	mRNA
VIBR0546_06027	VIBR0546_06027	hypothetical protein	0.053	−4.23997	3.12E-29	4.98E-28	yes	down	mRNA
VIBR0546_07999	VIBR0546_07999	hypothetical protein	5.507	2.461367	3.22E-29	5.13E-28	yes	up	mRNA
VIBR0546_17996	VIBR0546_17996	hypothetical protein	0.098	−3.34556	3.39E-29	5.36E-28	yes	down	mRNA
VIBR0546_06312	VIBR0546_06312	hypothetical protein	5.834	2.544421	3.43E-29	5.4E-28	yes	up	mRNA
VIBR0546_14235	VIBR0546_14235	hypothetical protein	0.126	−2.99267	4.38E-29	6.87E-28	yes	down	mRNA
VIBR0546_14575	VIBR0546_14575	glyceraldehyde-3-phosphate dehydrogenase	0.15	−2.73797	6.92E-29	1.08E-27	yes	down	mRNA
VIBR0546_11532	VIBR0546_11532	MutT/nudix family protein	0.101	−3.30824	7.74E-29	1.2E-27	yes	down	mRNA
VIBR0546_02955	VIBR0546_02955	acyl-CoA synthetase	0.098	−3.3503	1.13E-28	1.75E-27	yes	down	mRNA
VIBR0546_10934	VIBR0546_10934	electron transport complex protein RnfB	7.831	2.969232	1.21E-28	1.88E-27	yes	up	mRNA
VIBR0546_06512	VIBR0546_06512	glycine cleavage system regulatory protein	0.066	−3.92778	1.65E-28	2.54E-27	yes	down	mRNA
VIBR0546_03770	VIBR0546_03770	hypothetical protein	0.199	−2.33102	2E-28	3.07E-27	yes	down	mRNA
VIBR0546_17443	VIBR0546_17443	zinc-dependent hydrolase	0.153	−2.71187	2.02E-28	3.09E-27	yes	down	mRNA
VIBR0546_14435	VIBR0546_14435	methyl-accepting chemotaxis protein	0.172	−2.54103	2.07E-28	3.15E-27	yes	down	mRNA
VIBR0546_02254	VIBR0546_02254	tRNA-(MS[2]IO[6]A)-hydroxylase	5.41	2.43564	2.09E-28	3.18E-27	yes	up	mRNA
VIBR0546_16958	VIBR0546_16958	hydroxypyruvate isomerase	0.046	−4.44384	2.2E-28	3.33E-27	yes	down	mRNA
VIBR0546_01193	metL	bifunctional aspartate kinase II/homoserine dehydrogenase II	5.652	2.498719	2.24E-28	3.38E-27	yes	up	mRNA
VIBR0546_16923	VIBR0546_16923	phage integrase	4.726	2.240658	2.26E-28	3.4E-27	yes	up	mRNA
VIBR0546_16778	VIBR0546_16778	ABC-type transport system periplasmic substrate-binding protein	0.086	−3.54259	2.36E-28	3.53E-27	yes	down	mRNA
VIBR0546_18566	guaA	GMP synthase	4.278	2.096972	3.14E-28	4.69E-27	yes	up	mRNA
VIBR0546_01726	VIBR0546_01726	methyl-accepting chemotaxis protein	0.172	−2.53705	3.46E-28	5.14E-27	yes	down	mRNA

VIBR0546_07017	VIBR0546_07017	hypothetical protein	0.055	−4.18717	3.79E-28	5.62E-27	yes	down	mRNA
VIBR0546_08597	VIBR0546_08597	hypothetical protein	6.432	2.685274	5.09E-28	7.51E-27	yes	up	mRNA
VIBR0546_05832	VIBR0546_05832	regulatory P domain of the subtilisin-like proprotein convertase	0.089	−3.48243	5.61E-28	8.25E-27	yes	down	mRNA
VIBR0546_12502	VIBR0546_12502	tryptophanyl-tRNA synthetase II	8.802	3.137795	5.83E-28	8.55E-27	yes	up	mRNA
VIBR0546_18261	VIBR0546_18261	hypothetical protein	0.222	−2.17071	6.42E-28	9.39E-27	yes	down	mRNA
VIBR0546_02795	VIBR0546_02795	ATP-binding component of ABC transporter	0.095	−3.39525	6.67E-28	9.72E-27	yes	down	mRNA
VIBR0546_01906	VIBR0546_01906	transaldolase B	0.208	−2.2687	6.97E-28	1.01E-26	yes	down	mRNA
VIBR0546_10794	VIBR0546_10794	hypothetical protein	5.191	2.37599	7.15E-28	1.04E-26	yes	up	mRNA
VIBR0546_09072	VIBR0546_09072	integrase	7.282	2.864342	1.12E-27	1.62E-26	yes	up	mRNA
VIBR0546_04302	VIBR0546_04302	hypothetical protein	7.499	2.906607	1.14E-27	1.64E-26	yes	up	mRNA
VIBR0546_04082	VIBR0546_04082	LysR family transcriptional regulator	0.114	−3.13524	1.57E-27	2.25E-26	yes	down	mRNA
VIBR0546_07447	VIBR0546_07447	DedD protein	5.421	2.438483	1.64E-27	2.34E-26	yes	up	mRNA
VIBR0546_14560	VIBR0546_14560	hypothetical protein	0.232	−2.10953	1.84E-27	2.62E-26	yes	down	mRNA
VIBR0546_18241	VIBR0546_18241	transcriptional regulator	7.96	2.992799	1.93E-27	2.74E-26	yes	up	mRNA
VIBR0546_17658	VIBR0546_17658	guanine deaminase	0.185	−2.43824	2.17E-27	3.07E-26	yes	down	mRNA
VIBR0546_19689	VIBR0546_19689	C-di-GMP phosphodiesterase MbaA repressor of biofilm formation	10.614	3.407894	2.41E-27	3.4E-26	yes	up	mRNA
VIBR0546_18937	VIBR0546_18937	transporting ATPase	16.929	4.081448	2.49E-27	3.5E-26	yes	up	mRNA
VIBR0546_14130	rpmI	50S ribosomal protein L35	0.076	−3.71939	2.71E-27	3.79E-26	yes	down	mRNA
VIBR0546_09629	VIBR0546_09629	putative chitoporin	8.948	3.161607	3E-27	4.19E-26	yes	up	mRNA
VIBR0546_13287	VIBR0546_13287	putative hemolysin-type calcium-binding region	4.411	2.141052	3.51E-27	4.88E-26	yes	up	mRNA
VIBR0546_07974	VIBR0546_07974	chitin catabolic cascade sensor histidine kinase ChiS	8.446	3.07833	3.61E-27	5.01E-26	yes	up	mRNA
VIBR0546_18867	fliL	flagellar basal body-associated protein FliL	4.646	2.215962	4.05E-27	5.59E-26	yes	up	mRNA
VIBR0546_08772	VIBR0546_08772	preprotein translocase subunit SecB	0.118	−3.07772	4.4E-27	6.06E-26	yes	down	mRNA
VIBR0546_02149	VIBR0546_02149	starvation lipoprotein Slp like protein	4.353	2.122032	4.41E-27	6.06E-26	yes	up	mRNA
VIBR0546_04427	VIBR0546_04427	hypothetical protein	9.793	3.291706	5.41E-27	7.41E-26	yes	up	mRNA
VIBR0546_06712	VIBR0546_06712	peptidyl-prolyl cis-trans isomerase B	0.125	−2.99429	5.9E-27	8.05E-26	yes	down	mRNA
VIBR0546_20770	VIBR0546_20770	cell division protein FtsW	6.002	2.585364	7.19E-27	9.78E-26	yes	up	mRNA
VIBR0546_05798	VIBR0546_05798	thiamine monophosphate kinase	8.026	3.004673	7.68E-27	1.04E-25	yes	up	mRNA
VIBR0546_19684	VIBR0546_19684	hypothetical protein	5.98	2.580143	9.45E-27	1.28E-25	yes	up	mRNA
VIBR0546_11462	VIBR0546_11462	3-hydroxy-3-methylglutaryl-coenzyme A reductase	6.668	2.737258	1.12E-26	1.51E-25	yes	up	mRNA
VIBR0546_02164	VIBR0546_02164	putative ATP-dependent helicase	4.134	2.047431	1.2E-26	1.61E-25	yes	up	mRNA
VIBR0546_06807	VIBR0546_06807	Glyoxalase/Bleomycin resistance protein/Dioxygenase superfamily	0.178	−2.49264	1.24E-26	1.66E-25	yes	down	mRNA
VIBR0546_09414	VIBR0546_09414	hypothetical protein	6.619	2.72666	1.28E-26	1.71E-25	yes	up	mRNA
VIBR0546_07617	VIBR0546_07617	cytochrome c-type biogenesis protein	6.992	2.805639	1.34E-26	1.79E-25	yes	up	mRNA
VIBR0546_16036	VIBR0546_16036	hypothetical protein	0.045	−4.4797	1.44E-26	1.91E-25	yes	down	mRNA

VIBR0546_00030	VIBR0546_00030	hypothetical protein	0.112	−3.16113	1.75E-26	2.32E-25	yes	down	mRNA
VIBR0546_13167	VIBR0546_13167	hypothetical protein	0.1	−3.32469	2.28E-26	3E-25	yes	down	mRNA
VIBR0546_02484	VIBR0546_02484	hypothetical protein	5.409	2.435475	2.34E-26	3.08E-25	yes	up	mRNA
VIBR0546_04247	VIBR0546_04247	hypothetical protein	8.175	3.031287	2.4E-26	3.15E-25	yes	up	mRNA
VIBR0546_09909	VIBR0546_09909	hypothetical protein	0.064	−3.95948	2.56E-26	3.34E-25	yes	down	mRNA
VIBR0546_03957	VIBR0546_03957	sulfite reductase subunit beta	0.248	−2.0101	2.78E-26	3.62E-25	yes	down	mRNA
VIBR0546_17878	VIBR0546_17878	hypothetical protein	0.208	−2.26395	2.83E-26	3.68E-25	yes	down	mRNA
VIBR0546_09444	VIBR0546_09444	hypothetical protein	11.182	3.48308	2.89E-26	3.74E-25	yes	up	mRNA
VIBR0546_20368	VIBR0546_20368	chromosome segregation ATPase	0.126	−2.98946	3E-26	3.87E-25	yes	down	mRNA
VIBR0546_02850	VIBR0546_02850	hypothetical protein	0.047	−4.4089	3.81E-26	4.91E-25	yes	down	mRNA
VIBR0546_17698	VIBR0546_17698	hypothetical protein	0.09	−3.47528	4E-26	5.13E-25	yes	down	mRNA
VIBR0546_04257	ulaA	PTS system ascorbate-specific transporter subunit IIC	14.532	3.861136	4.01E-26	5.13E-25	yes	up	mRNA
VIBR0546_20565	VIBR0546_20565	hypothetical protein	0.198	−2.33342	5.31E-26	6.78E-25	yes	down	mRNA
VIBR0546_10399	VIBR0546_10399	histidine kinase	0.114	−3.13674	6.2E-26	7.89E-25	yes	down	mRNA
VIBR0546_12907	VIBR0546_12907	hypothetical protein	5.27	2.397748	6.9E-26	8.75E-25	yes	up	mRNA
VIBR0546_21830	VIBR0546_21830	hypothetical protein	6.206	2.633678	7.17E-26	9.07E-25	yes	up	mRNA
VIBR0546_11089	hemA	glutamyl-tRNA reductase	11.293	3.497363	7.71E-26	9.72E-25	yes	up	mRNA
VIBR0546_00820	VIBR0546_00820	hypothetical protein	0.092	−3.44542	7.88E-26	9.91E-25	yes	down	mRNA
VIBR0546_03867	VIBR0546_03867	hypothetical protein	8.303	3.053635	7.94E-26	9.95E-25	yes	up	mRNA
VIBR0546_11817	VIBR0546_11817	DNA-binding transcriptional repressor PuuR	0.158	−2.66255	8.48E-26	1.06E-24	yes	down	mRNA
VIBR0546_09729	VIBR0546_09729	penicillin-binding protein 2	5.624	2.491609	1E-25	1.25E-24	yes	up	mRNA
VIBR0546_10244	VIBR0546_10244	hypothetical protein	0.128	−2.96597	1.03E-25	1.27E-24	yes	down	mRNA
VIBR0546_17893	VIBR0546_17893	adenosine deaminase	0.238	−2.07397	1.27E-25	1.57E-24	yes	down	mRNA
VIBR0546_09017	VIBR0546_09017	zinc-binding alcohol dehydrogenase	0.168	−2.57118	1.33E-25	1.64E-24	yes	down	mRNA
VIBR0546_13422	VIBR0546_13422	general secretion pathway protein N	5.842	2.546582	1.72E-25	2.12E-24	yes	up	mRNA
VIBR0546_20900	VIBR0546_20900	esterase YqiA	11.529	3.527157	1.78E-25	2.19E-24	yes	up	mRNA
VIBR0546_04057	VIBR0546_04057	esterase	0.07	−3.83135	1.9E-25	2.32E-24	yes	down	mRNA
VIBR0546_13805	VIBR0546_13805	isoprenoid biosynthesis protein with amidotransferase-like domain	0.128	−2.97075	2.33E-25	2.84E-24	yes	down	mRNA
VIBR0546_11262	VIBR0546_11262	transcriptional regulator	0.131	−2.92739	3.32E-25	4.04E-24	yes	down	mRNA
VIBR0546_17683	VIBR0546_17683	GntR family transcriptional regulator	0.142	−2.82109	3.55E-25	4.32E-24	yes	down	mRNA
VIBR0546_09524	VIBR0546_09524	hypothetical protein	11.248	3.491564	3.56E-25	4.32E-24	yes	up	mRNA
VIBR0546_11235	VIBR0546_11235	hypothetical protein	0.112	−3.16195	3.66E-25	4.42E-24	yes	down	mRNA
VIBR0546_11487	VIBR0546_11487	hypothetical protein	0.107	−3.21779	3.74E-25	4.51E-24	yes	down	mRNA
VIBR0546_15496	VIBR0546_15496	cysteine/glutathione ABC transporter membrane/ATP-binding component	5.374	2.426025	4.36E-25	5.24E-24	yes	up	mRNA
VIBR0546_20223	VIBR0546_20223	hypothetical protein	7.326	2.872994	4.42E-25	5.28E-24	yes	up	mRNA
VIBR0546_21690	moaA	molybdenum cofactor biosynthesis protein A	6.436	2.686138	4.81E-25	5.73E-24	yes	up	mRNA

VIBR0546_00215	VIBR0546_00215	putative PAS/PAC sensor protein	0.238	−2.07169	6.47E-25	7.68E-24	yes	down	mRNA
VIBR0546_11842	VIBR0546_11842	succinate-semialdehyde dehydrogenase	0.179	−2.48148	7.56E-25	8.95E-24	yes	down	mRNA
VIBR0546_08862	VIBR0546_08862	chemotaxis signal transduction protein	0.17	−2.55335	9.36E-25	1.11E-23	yes	down	mRNA
VIBR0546_13885	VIBR0546_13885	hypothetical protein	0.082	−3.60927	1.08E-24	1.27E-23	yes	down	mRNA
VIBR0546_06707	VIBR0546_06707	ATP-dependent OLD family endonuclease	0.056	−4.15625	1.08E-24	1.27E-23	yes	down	mRNA
VIBR0546_03730	VIBR0546_03730	putative glutamate synthetase	0.118	−3.08886	1.17E-24	1.38E-23	yes	down	mRNA
VIBR0546_01406	VIBR0546_01406	hypothetical protein	7.709	2.946601	1.81E-24	2.12E-23	yes	up	mRNA
VIBR0546_03460	VIBR0546_03460	hypothetical protein	15.154	3.921619	2.03E-24	2.37E-23	yes	up	mRNA
VIBR0546_06667	VIBR0546_06667	hypothetical protein	10.432	3.382899	2.06E-24	2.4E-23	yes	up	mRNA
VIBR0546_01188	VIBR0546_01188	cystathionine gamma-synthase	7.325	2.872787	2.13E-24	2.47E-23	yes	up	mRNA
VIBR0546_19894	VIBR0546_19894	phosphatidate cytidylyltransferase	7.631	2.931807	2.16E-24	2.5E-23	yes	up	mRNA
VIBR0546_20695	VIBR0546_20695	autonomous glycyl radical cofactor GrcA	0.12	−3.06123	2.39E-24	2.75E-23	yes	down	mRNA
VIBR0546_00430	mobA	molybdopterin-guanine dinucleotide biosynthesis protein MobA	7.948	2.990547	2.61E-24	3E-23	yes	up	mRNA
VIBR0546_09212	VIBR0546_09212	hemolysin	5.37	2.424903	2.88E-24	3.29E-23	yes	up	mRNA
VIBR0546_10554	ubiE	ubiquinone/menaquinone biosynthesis methyltransferase	5.335	2.415486	3.95E-24	4.51E-23	yes	up	mRNA
VIBR0546_19317	pyrB	aspartate carbamoyltransferase catalytic subunit	0.077	−3.69931	4.33E-24	4.93E-23	yes	down	mRNA
VIBR0546_19624	VIBR0546_19624	hypothetical protein	6.475	2.694954	5.35E-24	6.07E-23	yes	up	mRNA
VIBR0546_17353	VIBR0546_17353	hypothetical protein	0.07	−3.83801	5.48E-24	6.21E-23	yes	down	mRNA
VIBR0546_11203	VIBR0546_11203	hypothetical protein	11.566	3.531846	5.72E-24	6.46E-23	yes	up	mRNA
VIBR0546_14545	VIBR0546_14545	cytosine deaminase	0.12	−3.06469	6.89E-24	7.76E-23	yes	down	mRNA
VIBR0546_07127	VIBR0546_07127	ABC-type phosphate transport system periplasmic component	0.027	−5.2343	7.43E-24	8.34E-23	yes	down	mRNA
VIBR0546_18366	VIBR0546_18366	phosphate ABC transporter%2C permease protein	7.291	2.866189	7.6E-24	8.51E-23	yes	up	mRNA
VIBR0546_01099	VIBR0546_01099	ribosome hibernation protein YhbH	0.134	−2.90494	8.04E-24	8.99E-23	yes	down	mRNA
VIBR0546_16963	VIBR0546_16963	putative oxidoreductase	0.076	−3.71135	8.44E-24	9.4E-23	yes	down	mRNA
VIBR0546_18556	xseA	exodeoxyribonuclease VII large subunit	4.32	2.111038	8.47E-24	9.41E-23	yes	up	mRNA
VIBR0546_08952	dnaA	chromosomal replication initiation protein	4.105	2.03747	9.03E-24	1E-22	yes	up	mRNA
VIBR0546_20560	VIBR0546_20560	succinyl-CoA synthetase subunit alpha	0.112	−3.15517	9.79E-24	1.08E-22	yes	down	mRNA
VIBR0546_10609	VIBR0546_10609	sensory box/GGDEF family protein	6.778	2.760811	9.86E-24	1.09E-22	yes	up	mRNA
VIBR0546_11327	VIBR0546_11327	nitrate reductase catalytic subunit	0.089	−3.49125	1.01E-23	1.11E-22	yes	down	mRNA
VIBR0546_19504	VIBR0546_19504	condesin subunit F	5.495	2.458188	1.1E-23	1.21E-22	yes	up	mRNA
VIBR0546_05922	VIBR0546_05922	hypothetical protein	0.142	−2.81939	1.1E-23	1.21E-22	yes	down	mRNA
VIBR0546_20318	VIBR0546_20318	putative outer membrane protein	0.051	−4.28333	1.21E-23	1.32E-22	yes	down	mRNA
VIBR0546_15616	VIBR0546_15616	hypothetical protein	4.379	2.130518	1.25E-23	1.36E-22	yes	up	mRNA
VIBR0546_05528	VIBR0546_05528	Tfp pilus assembly protein PilW	4.164	2.058079	1.36E-23	1.48E-22	yes	up	mRNA
VIBR0546_14595	VIBR0546_14595	acyl-CoA thioester hydrolase-like protein	0.132	−2.92205	1.4E-23	1.51E-22	yes	down	mRNA
VIBR0546_10384	VIBR0546_10384	hypothetical protein	0.138	−2.85576	1.54E-23	1.66E-22	yes	down	mRNA

VIBR0546_02720	VIBR0546_02720	chemotactic transducer-related protein	0.195	−2.35746	1.54E-23	1.66E-22	yes	down	mRNA
VIBR0546_17798	VIBR0546_17798	chloramphenicol acetyltransferase	0.077	−3.70689	1.59E-23	1.7E-22	yes	down	mRNA
VIBR0546_00165	VIBR0546_00165	ABC transporter ATPase component	4.499	2.169716	1.69E-23	1.81E-22	yes	up	mRNA
VIBR0546_04609	VIBR0546_04609	hypothetical protein	0.161	−2.63183	1.71E-23	1.82E-22	yes	down	mRNA
VIBR0546_10789	VIBR0546_10789	elongation factor P	0.179	−2.48566	1.73E-23	1.84E-22	yes	down	mRNA
VIBR0546_09304	VIBR0546_09304	flagellin	0.201	−2.31755	1.76E-23	1.87E-22	yes	down	mRNA
VIBR0546_09924	VIBR0546_09924	6-phospho-beta-glucosidase	0.183	−2.45031	1.84E-23	1.95E-22	yes	down	mRNA
VIBR0546_10624	VIBR0546_10624	putative multidrug resistance protein	0.2	−2.32526	2.02E-23	2.13E-22	yes	down	mRNA
VIBR0546_11617	VIBR0546_11617	polyhydroxyalkanoic acid synthase	0.079	−3.65546	2.03E-23	2.14E-22	yes	down	mRNA
VIBR0546_00859	VIBR0546_00859	hypothetical protein	0.182	−2.4599	2.04E-23	2.14E-22	yes	down	mRNA
VIBR0546_16536	VIBR0546_16536	response regulator	8.177	3.03151	2.06E-23	2.16E-22	yes	up	mRNA
VIBR0546_05972	VIBR0546_05972	D-lactate dehydrogenase	0.11	−3.18301	2.13E-23	2.22E-22	yes	down	mRNA
VIBR0546_04939	VIBR0546_04939	membrane-bound lytic murein transglycosylase C	4.56	2.189089	2.17E-23	2.26E-22	yes	up	mRNA
VIBR0546_04859	VIBR0546_04859	hypothetical protein	0.209	−2.2577	2.47E-23	2.56E-22	yes	down	mRNA
VIBR0546_17373	VIBR0546_17373	hypothetical protein	15.247	3.930424	2.48E-23	2.57E-22	yes	up	mRNA
VIBR0546_14615	malE	maltose ABC transporter periplasmic protein	0.161	−2.63507	3.55E-23	3.66E-22	yes	down	mRNA
VIBR0546_00405	VIBR0546_00405	hypothetical protein	6.125	2.614745	3.82E-23	3.93E-22	yes	up	mRNA
VIBR0546_10404	VIBR0546_10404	response regulator receiver modulated diguanylate phosphodiesterase	0.075	−3.72788	3.92E-23	4.02E-22	yes	down	mRNA
VIBR0546_18021	VIBR0546_18021	pilus assembly protein CpaC	0.135	−2.8854	4.23E-23	4.33E-22	yes	down	mRNA
VIBR0546_10694	VIBR0546_10694	hypothetical protein	5.023	2.32866	4.67E-23	4.75E-22	yes	up	mRNA
VIBR0546_01426	VIBR0546_01426	protein YtfJ precursor	0.163	−2.62076	4.72E-23	4.79E-22	yes	down	mRNA
VIBR0546_19884	VIBR0546_19884	membrane-associated zinc metalloprotease	4.249	2.087196	4.91E-23	4.97E-22	yes	up	mRNA
VIBR0546_10044	VIBR0546_10044	methyl-accepting chemotaxis protein	0.041	−4.60921	5.09E-23	5.14E-22	yes	down	mRNA
VIBR0546_07592	VIBR0546_07592	heme exporter protein C	4.816	2.267867	5.11E-23	5.15E-22	yes	up	mRNA
VIBR0546_10529	VIBR0546_10529	Sec-independent protein translocase protein	5.025	2.329128	5.56E-23	5.59E-22	yes	up	mRNA
VIBR0546_03410	VIBR0546_03410	maltose operon periplasmic protein	0.053	−4.24365	5.89E-23	5.91E-22	yes	down	mRNA
VIBR0546_08249	VIBR0546_08249	prephrenate dehydratase	0.108	−3.20853	6.29E-23	6.29E-22	yes	down	mRNA
VIBR0546_17703	VIBR0546_17703	hypothetical protein	0.186	−2.4263	6.42E-23	6.41E-22	yes	down	mRNA
VIBR0546_00590	VIBR0546_00590	putative sugar transferase	0.085	−3.56031	6.89E-23	6.86E-22	yes	down	mRNA
VIBR0546_15132	deoD	purine nucleoside phosphorylase	0.209	−2.26122	7.57E-23	7.52E-22	yes	down	mRNA
VIBR0546_13237	VIBR0546_13237	PrpE protein	0.172	−2.53567	8.45E-23	8.38E-22	yes	down	mRNA
VIBR0546_20995	VIBR0546_20995	ribosome maturation protein RimP	4.253	2.08865	8.57E-23	8.47E-22	yes	up	mRNA
VIBR0546_00879	VIBR0546_00879	hypothetical protein	6.468	2.69337	9.02E-23	8.9E-22	yes	up	mRNA
VIBR0546_17123	VIBR0546_17123	ABC transporter ATP-binding protein	0.131	−2.93552	9.33E-23	9.19E-22	yes	down	mRNA
VIBR0546_12707	VIBR0546_12707	hypothetical protein	10.718	3.421909	9.74E-23	9.56E-22	yes	up	mRNA
VIBR0546_16948	VIBR0546_16948	malate synthase	0.109	−3.19284	9.76E-23	9.56E-22	yes	down	mRNA
VIBR0546_17323	VIBR0546_17323	hypothetical protein	0.121	−3.04939	1.08E-22	1.05E-21	yes	down	mRNA

VIBR0546_04157	VIBR0546_04157	putative PTS system N-acetylglucosamine and glucose permease	6.098	2.608269	1.2E-22	1.17E-21	yes	up	mRNA
VIBR0546_07904	VIBR0546_07904	iron(III) ABC transporter%2C permease protein	5.855	2.549622	1.31E-22	1.28E-21	yes	up	mRNA
VIBR0546_12937	VIBR0546_12937	cobalamin synthase	6.887	2.783878	1.34E-22	1.3E-21	yes	up	mRNA
VIBR0546_02950	VIBR0546_02950	peptide transporter	0.113	−3.14385	1.47E-22	1.43E-21	yes	down	mRNA
VIBR0546_11572	VIBR0546_11572	AcrB/AcrD/AcrF family cation efflux system	0.048	−4.38815	1.5E-22	1.45E-21	yes	down	mRNA
VIBR0546_19889	VIBR0546_19889	1-deoxy-D-xylulose 5-phosphate reductoisomerase	6.324	2.660741	1.65E-22	1.59E-21	yes	up	mRNA
VIBR0546_10339	VIBR0546_10339	hypothetical protein	10.262	3.359215	1.87E-22	1.8E-21	yes	up	mRNA
VIBR0546_18371	VIBR0546_18371	ABC transporter transmembrane protein	4.851	2.278309	2.01E-22	1.93E-21	yes	up	mRNA
VIBR0546_17128	VIBR0546_17128	hypothetical protein	0.124	−3.01539	2.09E-22	2E-21	yes	down	mRNA
VIBR0546_11672	VIBR0546_11672	putative transporter	0.174	−2.52105	2.42E-22	2.32E-21	yes	down	mRNA
VIBR0546_02041	VIBR0546_02041	putative protease	7.662	2.937677	2.43E-22	2.32E-21	yes	up	mRNA
VIBR0546_19247	VIBR0546_19247	nicotinamide riboside transporter PnuC	5.319	2.411273	2.62E-22	2.5E-21	yes	up	mRNA
VIBR0546_02144	VIBR0546_02144	hypothetical protein	4.679	2.226298	2.87E-22	2.73E-21	yes	up	mRNA
VIBR0546_16346	VIBR0546_16346	lipooligosaccharided-glycero-D-manno-heptosyltransferase	4.594	2.199822	3.03E-22	2.87E-21	yes	up	mRNA
VIBR0546_11797	VIBR0546_11797	hypothetical protein	0.095	−3.39225	3.51E-22	3.31E-21	yes	down	mRNA
VIBR0546_09664	VIBR0546_09664	PTS system glucose-specific transporter subunit	0.206	−2.28243	3.91E-22	3.68E-21	yes	down	mRNA
VIBR0546_04839	VIBR0546_04839	5-formyltetrahydrofolate cyclo-ligase	6.342	2.664955	4.09E-22	3.84E-21	yes	up	mRNA
VIBR0546_07142	VIBR0546_07142	phosphate ABC transporter ATP-binding protein	0.061	−4.03576	4.7E-22	4.39E-21	yes	down	mRNA
VIBR0546_12012	VIBR0546_12012	methylated-DNA--protein-cysteine methyltransferase	0.206	−2.27953	5.1E-22	4.75E-21	yes	down	mRNA
VIBR0546_16076	VIBR0546_16076	cell division ATP-binding protein FtsE	4.65	2.217332	5.69E-22	5.28E-21	yes	up	mRNA
VIBR0546_19674	VIBR0546_19674	putative sigma-54 modulation protein	0.17	−2.55733	5.81E-22	5.39E-21	yes	down	mRNA
VIBR0546_03230	VIBR0546_03230	lipoprotein-releasing system ATP-binding protein LolD	0.043	−4.54953	6.19E-22	5.73E-21	yes	down	mRNA
VIBR0546_15936	VIBR0546_15936	putative ribosomal-protein-serine acetyltransferase	7.384	2.884443	6.59E-22	6.09E-21	yes	up	mRNA
VIBR0546_00760	VIBR0546_00760	hypothetical protein	16.692	4.061058	6.69E-22	6.17E-21	yes	up	mRNA
VIBR0546_12752	VIBR0546_12752	hypothetical protein	0.163	−2.61895	6.73E-22	6.18E-21	yes	down	mRNA
VIBR0546_08555	ruvB	Holliday junction DNA helicase RuvB	4.807	2.265194	8.19E-22	7.51E-21	yes	up	mRNA
VIBR0546_12892	VIBR0546_12892	cytolysin secretion protein VvhB	0.077	−3.7082	8.47E-22	7.76E-21	yes	down	mRNA
VIBR0546_12802	VIBR0546_12802	hypothetical protein	4.444	2.151706	8.87E-22	8.11E-21	yes	up	mRNA
VIBR0546_02750	VIBR0546_02750	hypothetical protein	0.127	−2.97708	9.86E-22	8.99E-21	yes	down	mRNA
VIBR0546_11832	VIBR0546_11832	putative carbon-nitrogen hydrolase	0.074	−3.74729	1.27E-21	1.15E-20	yes	down	mRNA
VIBR0546_09959	VIBR0546_09959	hypothetical protein	0.064	−3.96323	1.29E-21	1.17E-20	yes	down	mRNA
VIBR0546_14135	VIBR0546_14135	translation initiation factor 3	0.167	−2.57963	1.5E-21	1.36E-20	yes	down	mRNA
VIBR0546_20925	VIBR0546_20925	hypothetical protein	0.232	−2.10886	1.69E-21	1.53E-20	yes	down	mRNA
VIBR0546_02631	VIBR0546_02631	anti-RNA polymerase sigma 70 factor	0.226	−2.14502	1.73E-21	1.56E-20	yes	down	mRNA
VIBR0546_15631	VIBR0546_15631	DnaK-related protein	4.926	2.300424	2.01E-21	1.81E-20	yes	up	mRNA
VIBR0546_09779	VIBR0546_09779	hypothetical protein	10.882	3.443935	2.04E-21	1.83E-20	yes	up	mRNA
VIBR0546_15276	VIBR0546_15276	putative thiol oxidoreductase	7.141	2.8361	2.12E-21	1.89E-20	yes	up	mRNA

VIBR0546_21815	VIBR0546_21815	hypothetical protein	5.601	2.485764	2.12E-21	1.89E-20	yes	up	mRNA
VIBR0546_07849	VIBR0546_07849	glutamyl-Q tRNA(Asp) synthetase	7.117	2.831232	2.37E-21	2.11E-20	yes	up	mRNA
VIBR0546_15386	VIBR0546_15386	Tyrosine-specific transport protein	15.938	3.994405	2.57E-21	2.28E-20	yes	up	mRNA
VIBR0546_02920	VIBR0546_02920	exopolyphosphatase-like protein	0.163	−2.61931	2.58E-21	2.28E-20	yes	down	mRNA
VIBR0546_13332	VIBR0546_13332	hypothetical protein	4.225	2.079057	2.61E-21	2.3E-20	yes	up	mRNA
VIBR0546_07317	VIBR0546_07317	putative aminotransferase	0.12	−3.05875	2.67E-21	2.35E-20	yes	down	mRNA
VIBR0546_18787	VIBR0546_18787	flagellin	0.213	−2.23162	2.77E-21	2.43E-20	yes	down	mRNA
VIBR0546_19312	VIBR0546_19312	aspartate carbamoyltransferase regulatory subunit	0.059	−4.08121	2.84E-21	2.49E-20	yes	down	mRNA
VIBR0546_10354	VIBR0546_10354	deoxyribodipyrimidine photolyase	9.096	3.185228	3.04E-21	2.66E-20	yes	up	mRNA
VIBR0546_19988	VIBR0546_19988	putative two-component response regulator	6.856	2.777432	3.47E-21	3.02E-20	yes	up	mRNA
VIBR0546_10419	VIBR0546_10419	hypothetical protein	0.137	−2.86986	3.53E-21	3.07E-20	yes	down	mRNA
VIBR0546_10169	VIBR0546_10169	maltose/maltodextrin-binding protein	12.614	3.656934	3.71E-21	3.22E-20	yes	up	mRNA
VIBR0546_20570	VIBR0546_20570	hypothetical protein	0.138	−2.85697	3.89E-21	3.37E-20	yes	down	mRNA
VIBR0546_17318	VIBR0546_17318	phosphotransferase system EIIC	0.088	−3.50446	3.94E-21	3.4E-20	yes	down	mRNA
VIBR0546_11407	VIBR0546_11407	GGDEF family protein	0.178	−2.4902	3.95E-21	3.41E-20	yes	down	mRNA
VIBR0546_07517	VIBR0546_07517	phosphohistidine phosphatase	6.193	2.630575	4.59E-21	3.95E-20	yes	up	mRNA
VIBR0546_19227	VIBR0546_19227	ABC-type amino acid transport/signal transduction systems%2C periplasmic component	6.225	2.638014	4.63E-21	3.98E-20	yes	up	mRNA
VIBR0546_08480	VIBR0546_08480	hypothetical protein	4.079	2.028062	4.7E-21	4.03E-20	yes	up	mRNA
VIBR0546_10219	VIBR0546_10219	hypothetical protein	0.169	−2.56791	4.74E-21	4.06E-20	yes	down	mRNA
VIBR0546_03897	cysN	sulfate adenylyltransferase subunit 1	0.171	−2.55143	5.01E-21	4.28E-20	yes	down	mRNA
VIBR0546_10059	VIBR0546_10059	chemotaxis protein histidine kinase	0.077	−3.69438	5.24E-21	4.47E-20	yes	down	mRNA
VIBR0546_07642	VIBR0546_07642	putative long-chain fatty acid transport protein	0.197	−2.34328	5.32E-21	4.52E-20	yes	down	mRNA
VIBR0546_07302	VIBR0546_07302	gamma-glutamylputrescine oxidase	0.159	−2.65468	5.37E-21	4.56E-20	yes	down	mRNA
VIBR0546_00170	VIBR0546_00170	hypothetical protein	4.186	2.065688	5.64E-21	4.78E-20	yes	up	mRNA
VIBR0546_06127	VIBR0546_06127	VBCS repeat-containing protein	0.128	−2.96195	6.18E-21	5.22E-20	yes	down	mRNA
VIBR0546_13865	VIBR0546_13865	SAM-dependent methyltransferase	0.221	−2.17734	6.53E-21	5.5E-20	yes	down	mRNA
VIBR0546_11322	VIBR0546_11322	NapD protein	0.145	−2.78098	7.02E-21	5.9E-20	yes	down	mRNA
VIBR0546_16301	VIBR0546_16301	hypothetical protein	5.342	2.417417	7.07E-21	5.93E-20	yes	up	mRNA
VIBR0546_15446	VIBR0546_15446	hypothetical protein	0.095	−3.38954	7.23E-21	6.06E-20	yes	down	mRNA
VIBR0546_07682	VIBR0546_07682	hypothetical protein	6.224	2.637942	7.72E-21	6.45E-20	yes	up	mRNA
VIBR0546_06422	VIBR0546_06422	thymidine kinase	8.999	3.169686	8.17E-21	6.8E-20	yes	up	mRNA
VIBR0546_03470	VIBR0546_03470	hypothetical protein	20.556	4.361508	1.06E-20	8.81E-20	yes	up	mRNA
VIBR0546_09057	VIBR0546_09057	putative acyltransferase	7.555	2.917394	1.08E-20	8.93E-20	yes	up	mRNA
VIBR0546_10944	rnfD	electron transport complex protein RnfD	7.388	2.885133	1.1E-20	9.11E-20	yes	up	mRNA
VIBR0546_00360	VIBR0546_00360	hypothetical protein	7.726	2.949799	1.16E-20	9.55E-20	yes	up	mRNA
VIBR0546_13172	VIBR0546_13172	hypothetical protein	0.112	−3.15424	1.19E-20	9.74E-20	yes	down	mRNA
VIBR0546_17513	VIBR0546_17513	putative transporter	0.097	−3.36138	1.21E-20	9.94E-20	yes	down	mRNA

VIBR0546_01221	VIBR0546_01221	small protein A	12.414	3.633869	1.25E-20	1.02E-19	yes	up	mRNA
VIBR0546_01321	VIBR0546_01321	hypothetical protein	12.352	3.626707	1.31E-20	1.07E-19	yes	up	mRNA
VIBR0546_20825	VIBR0546_20825	phosphoheptose isomerase	5.519	2.464439	1.51E-20	1.23E-19	yes	up	mRNA
VIBR0546_19377	VIBR0546_19377	lipopolysaccharide ABC transporter permease	4.438	2.149862	1.53E-20	1.24E-19	yes	up	mRNA
VIBR0546_05458	VIBR0546_05458	regulatory protein CsrD	5.537	2.469106	1.57E-20	1.28E-19	yes	up	mRNA
VIBR0546_10204	VIBR0546_10204	Oxidoreductase%2C short-chain dehydrogenase/reductase family protein	0.201	−2.31446	1.65E-20	1.33E-19	yes	down	mRNA
VIBR0546_16056	VIBR0546_16056	antibiotic transporter	8.337	3.059488	1.85E-20	1.49E-19	yes	up	mRNA
VIBR0546_04794	VIBR0546_04794	hypothetical protein	4.939	2.304346	1.9E-20	1.53E-19	yes	up	mRNA
VIBR0546_12522	VIBR0546_12522	periplasmic substrate-binding transport protein%2C putative	0.153	−2.71246	1.93E-20	1.55E-19	yes	down	mRNA
VIBR0546_01936	rbgA	GTPase YlqF	0.183	−2.45208	1.93E-20	1.55E-19	yes	down	mRNA
VIBR0546_05239	rpsU	30S ribosomal protein S21	0.191	−2.38902	1.94E-20	1.55E-19	yes	down	mRNA
VIBR0546_13382	VIBR0546_13382	general secretion pathway protein F	5.223	2.384765	1.95E-20	1.56E-19	yes	up	mRNA
VIBR0546_13162	VIBR0546_13162	hypothetical protein	0.121	−3.04635	2.17E-20	1.73E-19	yes	down	mRNA
VIBR0546_07372	VIBR0546_07372	hypothetical protein	0.174	−2.52388	2.35E-20	1.87E-19	yes	down	mRNA
VIBR0546_19459	VIBR0546_19459	scaffold protein	5.237	2.388747	2.61E-20	2.07E-19	yes	up	mRNA
VIBR0546_11074	VIBR0546_11074	hypothetical protein	23.034	4.52567	2.76E-20	2.19E-19	yes	up	mRNA
VIBR0546_15586	VIBR0546_15586	ATP-dependent RNA helicase HrpA	4.015	2.005562	2.81E-20	2.22E-19	yes	up	mRNA
VIBR0546_11198	VIBR0546_11198	sulfur oxidation protein dsrF	18.349	4.197622	3.64E-20	2.87E-19	yes	up	mRNA
VIBR0546_04142	VIBR0546_04142	hypothetical protein	7.488	2.904529	3.81E-20	2.99E-19	yes	up	mRNA
VIBR0546_07012	VIBR0546_07012	thioesterase	0.091	−3.45681	3.83E-20	3E-19	yes	down	mRNA
VIBR0546_08917	VIBR0546_08917	hypothetical protein	6.663	2.736211	3.89E-20	3.05E-19	yes	up	mRNA
VIBR0546_01089	VIBR0546_01089	ABC-type transport system%2C ATPase component	4.217	2.076077	4.24E-20	3.31E-19	yes	up	mRNA
VIBR0546_19167	VIBR0546_19167	1-aminocyclopropane-1-carboxylate deaminase	5.383	2.428365	4.35E-20	3.38E-19	yes	up	mRNA
VIBR0546_03630	VIBR0546_03630	TRAP dicarboxylate family transporter%2C DctP subunit	0.083	−3.5894	5.09E-20	3.95E-19	yes	down	mRNA
VIBR0546_13312	glnA	glutamine synthetase	0.211	−2.24747	5.81E-20	4.5E-19	yes	down	mRNA
VIBR0546_17363	VIBR0546_17363	hypothetical protein	0.149	−2.74536	5.87E-20	4.54E-19	yes	down	mRNA
VIBR0546_05129	mtlR	mannitol repressor protein	5.782	2.531496	6.47E-20	5E-19	yes	up	mRNA
VIBR0546_20423	VIBR0546_20423	hypothetical protein	0.12	−3.05819	6.94E-20	5.35E-19	yes	down	mRNA
VIBR0546_21025	VIBR0546_21025	hypothetical protein	4.566	2.191075	7.77E-20	5.97E-19	yes	up	mRNA
VIBR0546_18281	VIBR0546_18281	putative acetoin utilization protein	0.208	−2.26641	8.48E-20	6.49E-19	yes	down	mRNA
VIBR0546_06337	VIBR0546_06337	hypothetical protein	6.173	2.625867	8.58E-20	6.56E-19	yes	up	mRNA
VIBR0546_19854	lpxB	lipid-A-disaccharide synthase	4.621	2.208186	1.05E-19	7.99E-19	yes	up	mRNA
VIBR0546_00150	VIBR0546_00150	hypothetical protein	10.139	3.341913	1.09E-19	8.33E-19	yes	up	mRNA
VIBR0546_03150	VIBR0546_03150	soxR protein	0.119	−3.06731	1.1E-19	8.38E-19	yes	down	mRNA
VIBR0546_18581	VIBR0546_18581	sodium/alanine symporter	0.171	−2.55151	1.12E-19	8.47E-19	yes	down	mRNA
VIBR0546_10054	VIBR0546_10054	purine-binding chemotaxis protein CheW	0.083	−3.59796	1.29E-19	9.75E-19	yes	down	mRNA
VIBR0546_06987	VIBR0546_06987	hypothetical protein	0.074	−3.75082	1.34E-19	1.01E-18	yes	down	mRNA

VIBR0546_19579	clpA	ATP-dependent Clp protease ATP-binding subunit	0.249	−2.00495	1.35E-19	1.01E-18	yes	down	mRNA
VIBR0546_00045	VIBR0546_00045	amino acid ABC transporter%2C permease protein	0.217	−2.20557	1.4E-19	1.06E-18	yes	down	mRNA
VIBR0546_20213	VIBR0546_20213	hypothetical protein	4.4	2.13766	1.54E-19	1.16E-18	yes	up	mRNA
VIBR0546_11737	VIBR0546_11737	L-2%2C4-diaminobutyric acid acetyltransferase	22.352	4.482305	1.69E-19	1.26E-18	yes	up	mRNA
VIBR0546_14245	VIBR0546_14245	hypothetical protein	0.231	−2.11457	1.81E-19	1.35E-18	yes	down	mRNA
VIBR0546_07572	VIBR0546_07572	chemotaxis signal transduction protein	0.192	−2.37791	1.85E-19	1.38E-18	yes	down	mRNA
VIBR0546_15426	VIBR0546_15426	hypothetical protein	0.204	−2.29504	1.96E-19	1.45E-18	yes	down	mRNA
VIBR0546_05573	VIBR0546_05573	hypothetical protein	4.4	2.137582	2E-19	1.48E-18	yes	up	mRNA
VIBR0546_11827	VIBR0546_11827	putative oxidoreductase	0.093	−3.42777	2.15E-19	1.59E-18	yes	down	mRNA
VIBR0546_06247	VIBR0546_06247	hypothetical protein	0.117	−3.09265	2.24E-19	1.66E-18	yes	down	mRNA
VIBR0546_06802	VIBR0546_06802	glyoxalase family protein	0.205	−2.28466	2.43E-19	1.79E-18	yes	down	mRNA
VIBR0546_03380	VIBR0546_03380	hypothetical protein	0.21	−2.24907	2.5E-19	1.83E-18	yes	down	mRNA
VIBR0546_09899	VIBR0546_09899	LysR family transcriptional regulator	0.224	−2.15748	2.5E-19	1.83E-18	yes	down	mRNA
VIBR0546_16341	VIBR0546_16341	3-deoxy-D-manno-octulosonic-acid kinase	4.236	2.082798	2.56E-19	1.88E-18	yes	up	mRNA
VIBR0546_08962	recF	recombination protein F	6.529	2.706791	2.58E-19	1.89E-18	yes	up	mRNA
VIBR0546_11337	VIBR0546_11337	periplasmic nitrate reductase%2C cytochrome c-type protein	0.142	−2.81284	2.71E-19	1.98E-18	yes	down	mRNA
VIBR0546_01014	VIBR0546_01014	methyl-accepting chemotaxis protein	0.131	−2.93766	2.9E-19	2.12E-18	yes	down	mRNA
VIBR0546_09769	VIBR0546_09769	hypothetical protein	0.155	−2.68774	3.39E-19	2.47E-18	yes	down	mRNA
VIBR0546_13750	VIBR0546_13750	hypothetical protein	0.031	−5.02205	3.59E-19	2.6E-18	yes	down	mRNA
VIBR0546_09929	VIBR0546_09929	universal stress protein family 8	0.16	−2.64695	3.93E-19	2.85E-18	yes	down	mRNA
VIBR0546_01631	VIBR0546_01631	DNA-binding transcriptional regulator FruR	0.175	−2.51698	3.94E-19	2.85E-18	yes	down	mRNA
VIBR0546_10839	VIBR0546_10839	hypothetical protein	7.674	2.939943	4.28E-19	3.08E-18	yes	up	mRNA
VIBR0546_16868	VIBR0546_16868	hypothetical protein	0.212	−2.24056	4.34E-19	3.13E-18	yes	down	mRNA
VIBR0546_16461	VIBR0546_16461	Coenzyme F390 synthetase-like protein	4.486	2.165305	4.57E-19	3.29E-18	yes	up	mRNA
VIBR0546_19182	VIBR0546_19182	PTS system fructose-specific transporter subunit IIABC	5.066	2.340735	4.63E-19	3.32E-18	yes	up	mRNA
VIBR0546_13242	VIBR0546_13242	hypothetical protein	9.006	3.170925	4.68E-19	3.35E-18	yes	up	mRNA
VIBR0546_10134	VIBR0546_10134	hypothetical protein	10.775	3.429588	4.69E-19	3.35E-18	yes	up	mRNA
VIBR0546_15791	VIBR0546_15791	putative transporter	5.153	2.365302	5.33E-19	3.81E-18	yes	up	mRNA
VIBR0546_02464	VIBR0546_02464	transcriptional regulator	7.579	2.921974	5.44E-19	3.88E-18	yes	up	mRNA
VIBR0546_17008	VIBR0546_17008	keto-hydroxyglutarate-aldolase/keto-deoxy-phosphogluconate aldolase	0.174	−2.52224	5.82E-19	4.14E-18	yes	down	mRNA
VIBR0546_04559	VIBR0546_04559	methyl-accepting chemotaxis protein	4.074	2.026615	7.06E-19	5.01E-18	yes	up	mRNA
VIBR0546_20765	murG	undecaprenyldiphospho-muramoylpentapeptide beta-N-acetylglucosaminyltransferase	4.697	2.231824	7.29E-19	5.16E-18	yes	up	mRNA
VIBR0546_18451	VIBR0546_18451	small integral membrane protein-like protein	0.067	−3.89423	8.25E-19	5.83E-18	yes	down	mRNA
VIBR0546_17488	VIBR0546_17488	hypothetical protein	0.086	−3.53749	8.45E-19	5.97E-18	yes	down	mRNA
VIBR0546_15461	VIBR0546_15461	hypothetical protein	10.215	3.35265	9.41E-19	6.63E-18	yes	up	mRNA
VIBR0546_01566	pntA	NAD(P) transhydrogenase subunit alpha	0.217	−2.20735	1.01E-18	7.08E-18	yes	down	mRNA

VIBR0546_03200	VIBR0546_03200	hypothetical protein	0.232	−2.10805	1.19E-18	8.35E-18	yes	down	mRNA
VIBR0546_06677	VIBR0546_06677	Outer membrane receptor protein	5.357	2.421524	1.21E-18	8.44E-18	yes	up	mRNA
VIBR0546_08847	VIBR0546_08847	Signal transduction histidine kinase	0.129	−2.95993	1.29E-18	9.03E-18	yes	down	mRNA
VIBR0546_17348	VIBR0546_17348	hypothetical protein	0.144	−2.79712	1.33E-18	9.31E-18	yes	down	mRNA
VIBR0546_04262	VIBR0546_04262	PTS system%2C IIB component	18.065	4.175157	1.34E-18	9.32E-18	yes	up	mRNA
VIBR0546_05548	VIBR0546_05548	rod shape-determining protein MreC	6.858	2.777884	1.51E-18	1.05E-17	yes	up	mRNA
VIBR0546_14210	VIBR0546_14210	hypothetical protein	0.177	−2.50059	1.65E-18	1.14E-17	yes	down	mRNA
VIBR0546_02369	VIBR0546_02369	Signal transduction histidine kinase	4.893	2.290669	1.76E-18	1.22E-17	yes	up	mRNA
VIBR0546_12942	cobU	adenosylcobinamide kinase/adenosylcobinamide-phosphate guanylyltransferase	6.129	2.615597	2.03E-18	1.4E-17	yes	up	mRNA
VIBR0546_08667	VIBR0546_08667	hypothetical protein	5.517	2.463827	2.17E-18	1.49E-17	yes	up	mRNA
VIBR0546_02001	VIBR0546_02001	hypothetical protein	0.118	−3.07775	2.17E-18	1.49E-17	yes	down	mRNA
VIBR0546_19744	VIBR0546_19744	efflux pump component MtrF	8.078	3.013927	2.18E-18	1.49E-17	yes	up	mRNA
VIBR0546_13137	VIBR0546_13137	putative protein disaggregation chaperone	0.093	−3.41941	2.49E-18	1.7E-17	yes	down	mRNA
VIBR0546_14185	VIBR0546_14185	anti-anti-sigma regulatory factor	0.103	−3.27363	2.62E-18	1.79E-17	yes	down	mRNA
VIBR0546_06122	VIBR0546_06122	ABC transporter: Membrane fusion protein	0.113	−3.14071	2.65E-18	1.81E-17	yes	down	mRNA
VIBR0546_10429	VIBR0546_10429	putative acetyltransferase	0.217	−2.20452	2.8E-18	1.91E-17	yes	down	mRNA
VIBR0546_05438	uvrA	excinuclease ABC subunit A	4.897	2.292029	2.82E-18	1.92E-17	yes	up	mRNA
VIBR0546_14712	VIBR0546_14712	nucleoid DNA-binding protein	0.203	−2.2973	2.96E-18	2.01E-17	yes	down	mRNA
VIBR0546_06087	VIBR0546_06087	alkaline serine exoprotease A precursor	0.193	−2.37258	3.02E-18	2.04E-17	yes	down	mRNA
VIBR0546_08470	VIBR0546_08470	hypothetical protein	8.502	3.087874	3.73E-18	2.52E-17	yes	up	mRNA
VIBR0546_17103	VIBR0546_17103	hypothetical protein	0.145	−2.7818	3.78E-18	2.55E-17	yes	down	mRNA
VIBR0546_05024	VIBR0546_05024	glutathione synthetase	0.219	−2.19003	3.82E-18	2.57E-17	yes	down	mRNA
VIBR0546_20053	VIBR0546_20053	hypothetical protein	0.178	−2.4927	3.96E-18	2.66E-17	yes	down	mRNA
VIBR0546_04232	VIBR0546_04232	hypothetical protein	6.057	2.59853	4.24E-18	2.85E-17	yes	up	mRNA
VIBR0546_18256	VIBR0546_18256	hypothetical protein	5.856	2.549798	4.29E-18	2.87E-17	yes	up	mRNA
VIBR0546_15981	VIBR0546_15981	hypothetical protein	0.116	−3.11007	4.31E-18	2.88E-17	yes	down	mRNA
VIBR0546_13980	VIBR0546_13980	hypothetical protein	0.193	−2.37175	4.53E-18	3.03E-17	yes	down	mRNA
VIBR0546_11537	VIBR0546_11537	hypothetical protein	0.209	−2.25937	4.63E-18	3.08E-17	yes	down	mRNA
VIBR0546_00904	VIBR0546_00904	transcriptional activator	0.248	−2.0141	4.63E-18	3.08E-17	yes	down	mRNA
VIBR0546_03130	VIBR0546_03130	GGDEF family protein	0.085	−3.54828	4.98E-18	3.3E-17	yes	down	mRNA
VIBR0546_18216	VIBR0546_18216	hypothetical protein	7.755	2.955153	5.29E-18	3.49E-17	yes	up	mRNA
VIBR0546_13925	VIBR0546_13925	putative ABC transporter ATP-binding protein	0.062	−4.01675	6.31E-18	4.16E-17	yes	down	mRNA
VIBR0546_05728	VIBR0546_05728	aminoacyl-histidine dipeptidase	0.205	−2.28921	6.54E-18	4.31E-17	yes	down	mRNA
VIBR0546_04342	VIBR0546_04342	DNA polymerase III subunit delta'	4.956	2.309189	6.61E-18	4.35E-17	yes	up	mRNA
VIBR0546_03075	VIBR0546_03075	hypothetical protein	0.199	−2.33064	6.93E-18	4.55E-17	yes	down	mRNA
VIBR0546_18947	VIBR0546_18947	putative cytoplasmic protein	0.087	−3.51648	7.17E-18	4.7E-17	yes	down	mRNA
VIBR0546_07472	VIBR0546_07472	hypothetical protein	5.542	2.470466	7.25E-18	4.74E-17	yes	up	mRNA

VIBR0546_07342	VIBR0546_07342	transcriptional regulator%2C XRE family protein	0.097	−3.36315	7.29E-18	4.76E-17	yes	down	mRNA
VIBR0546_04754	rnc	ribonuclease III	4.266	2.092903	7.51E-18	4.9E-17	yes	up	mRNA
VIBR0546_14977	VIBR0546_14977	chlorohydrolase/deaminase family protein	5.252	2.392779	7.58E-18	4.94E-17	yes	up	mRNA
VIBR0546_15621	VIBR0546_15621	GGDEF family protein	5.17	2.370121	8.05E-18	5.23E-17	yes	up	mRNA
VIBR0546_02705	VIBR0546_02705	hypothetical protein	0.11	−3.18637	8.4E-18	5.46E-17	yes	down	mRNA
VIBR0546_16551	VIBR0546_16551	putative integral membrane protein	4.318	2.110522	9.5E-18	6.15E-17	yes	up	mRNA
VIBR0546_16196	VIBR0546_16196	sensor histidine kinase	4.16	2.056412	9.68E-18	6.26E-17	yes	up	mRNA
VIBR0546_11717	VIBR0546_11717	hypothetical protein	10.026	3.325667	1.02E-17	6.56E-17	yes	up	mRNA
VIBR0546_09107	VIBR0546_09107	hypothetical protein	4.671	2.22386	1.05E-17	6.74E-17	yes	up	mRNA
VIBR0546_07622	VIBR0546_07622	putative cytochrome c-type biogenesis protein	4.138	2.048852	1.32E-17	8.47E-17	yes	up	mRNA
VIBR0546_01481	VIBR0546_01481	hypothetical protein	0.061	−4.02644	1.7E-17	1.09E-16	yes	down	mRNA
VIBR0546_08565	VIBR0546_08565	hypothetical protein	4.973	2.314134	1.86E-17	1.19E-16	yes	up	mRNA
VIBR0546_20453	VIBR0546_20453	ATP-dependent DNA helicase RecQ	4.884	2.288063	1.87E-17	1.19E-16	yes	up	mRNA
VIBR0546_11792	VIBR0546_11792	NadC family protein	0.175	−2.51383	1.94E-17	1.23E-16	yes	down	mRNA
VIBR0546_18051	VIBR0546_18051	Flp pilus assembly protein	0.192	−2.38313	2.06E-17	1.31E-16	yes	down	mRNA
VIBR0546_01241	VIBR0546_01241	proton/glutamate symport protein	4.243	2.085244	2.23E-17	1.41E-16	yes	up	mRNA
VIBR0546_13610	VIBR0546_13610	diguanylate cyclase/phosphodiesterase domain 2	0.1	−3.31913	2.23E-17	1.41E-16	yes	down	mRNA
VIBR0546_19968	VIBR0546_19968	two component transcriptional regulator%2C winged helix family protein	18.15	4.181895	2.37E-17	1.5E-16	yes	up	mRNA
VIBR0546_14025	VIBR0546_14025	Glyoxalase/bleomycin resistance protein/dioxygenase	0.077	−3.69863	2.62E-17	1.66E-16	yes	down	mRNA
VIBR0546_17548	VIBR0546_17548	methyl-accepting chemotaxis sensory transducer	0.106	−3.2408	2.69E-17	1.7E-16	yes	down	mRNA
VIBR0546_06072	VIBR0546_06072	MerR family transcriptional regulator	0.086	−3.54555	2.89E-17	1.81E-16	yes	down	mRNA
VIBR0546_08647	VIBR0546_08647	hypothetical protein	4.245	2.085596	3.03E-17	1.9E-16	yes	up	mRNA
VIBR0546_11787	VIBR0546_11787	putative hydrolase or acyltransferase	0.16	−2.64086	3.08E-17	1.93E-16	yes	down	mRNA
VIBR0546_10049	VIBR0546_10049	chemotaxis signal transduction protein	0.099	−3.34324	3.1E-17	1.94E-16	yes	down	mRNA
VIBR0546_20228	VIBR0546_20228	ISSwp2%2C transposase	0.202	−2.31113	3.29E-17	2.05E-16	yes	down	mRNA
VIBR0546_12722	VIBR0546_12722	putative threonine efflux protein	4.404	2.138921	3.57E-17	2.22E-16	yes	up	mRNA
VIBR0546_12962	VIBR0546_12962	vtamin B12-transporter permease	8.15	3.026733	3.76E-17	2.34E-16	yes	up	mRNA
VIBR0546_12487	VIBR0546_12487	transporter AcrB/D/F family protein	4.515	2.174708	4.26E-17	2.65E-16	yes	up	mRNA
VIBR0546_03220	VIBR0546_03220	hypothetical protein	0.092	−3.44575	4.41E-17	2.74E-16	yes	down	mRNA
VIBR0546_00035	VIBR0546_00035	phosphate ABC transporter ATP-binding protein	0.101	−3.31277	4.58E-17	2.83E-16	yes	down	mRNA
VIBR0546_07062	VIBR0546_07062	hypothetical protein	0.158	−2.66458	5.49E-17	3.38E-16	yes	down	mRNA
VIBR0546_08737	hslU	ATP-dependent protease ATP-binding subunit HslU	0.134	−2.8995	5.61E-17	3.45E-16	yes	down	mRNA
VIBR0546_03977	VIBR0546_03977	hypothetical protein	6.035	2.593366	5.65E-17	3.47E-16	yes	up	mRNA
VIBR0546_18036	VIBR0546_18036	hypothetical protein	0.173	−2.52736	6.37E-17	3.89E-16	yes	down	mRNA
VIBR0546_18391	VIBR0546_18391	hypothetical protein	7.41	2.889492	6.97E-17	4.25E-16	yes	up	mRNA
VIBR0546_04352	VIBR0546_04352	hypothetical protein	4.973	2.314164	7.43E-17	4.53E-16	yes	up	mRNA
VIBR0546_10154	VIBR0546_10154	Neopullulanase	4.992	2.319677	7.5E-17	4.56E-16	yes	up	mRNA

VIBR0546_13032	VIBR0546_13032	homoserine O-succinyltransferase	5.779	2.530767	9.51E-17	5.77E-16	yes	up	mRNA
VIBR0546_05937	lldD	L-lactate dehydrogenase	0.158	−2.66432	1.01E-16	6.14E-16	yes	down	mRNA
VIBR0546_01198	metF	5%2C10-methylenetetrahydrofolate reductase	0.237	−2.07713	1.11E-16	6.72E-16	yes	down	mRNA
VIBR0546_16186	VIBR0546_16186	hypothetical protein	7.492	2.90544	1.14E-16	6.87E-16	yes	up	mRNA
VIBR0546_14240	VIBR0546_14240	hypothetical protein	0.227	−2.14126	1.29E-16	7.78E-16	yes	down	mRNA
VIBR0546_05683	VIBR0546_05683	hypothetical protein	6.968	2.800756	1.44E-16	8.64E-16	yes	up	mRNA
VIBR0546_13417	VIBR0546_13417	type II secretory pathway%2C component EpsM	4.856	2.27968	1.51E-16	9.05E-16	yes	up	mRNA
VIBR0546_01696	mgsA	methylglyoxal synthase	0.103	−3.28525	1.74E-16	1.04E-15	yes	down	mRNA
VIBR0546_04187	VIBR0546_04187	2-succinyl-5-enolpyruvyl-6-hydroxy-3-cyclohexene-1-carboxylate synthase	4.946	2.306144	1.74E-16	1.04E-15	yes	up	mRNA
VIBR0546_18016	VIBR0546_18016	Flp pilus assembly protein CpaB	0.151	−2.73059	1.84E-16	1.09E-15	yes	down	mRNA
VIBR0546_14480	VIBR0546_14480	hypothetical protein	0.171	−2.54851	1.85E-16	1.1E-15	yes	down	mRNA
VIBR0546_18967	VIBR0546_18967	3-oxoacyl-(acyl carrier protein) synthase II	4.337	2.116789	1.92E-16	1.14E-15	yes	up	mRNA
VIBR0546_16863	VIBR0546_16863	hypothetical protein	0.136	−2.88056	2.03E-16	1.2E-15	yes	down	mRNA
VIBR0546_10009	VIBR0546_10009	hypothetical protein	0.202	−2.3064	2.08E-16	1.23E-15	yes	down	mRNA
VIBR0546_10409	VIBR0546_10409	hypothetical protein	0.065	−3.94344	2.25E-16	1.33E-15	yes	down	mRNA
VIBR0546_08334	VIBR0546_08334	MshA%2C mannose-sensitive hemaglutinin	7.673	2.939854	2.28E-16	1.34E-15	yes	up	mRNA
VIBR0546_08922	rnpA	ribonuclease P	10.26	3.358937	2.35E-16	1.38E-15	yes	up	mRNA
VIBR0546_20665	VIBR0546_20665	5'-nucleotidase	0.176	−2.50572	2.48E-16	1.46E-15	yes	down	mRNA
VIBR0546_11807	VIBR0546_11807	putative glutamine synthetase	0.042	−4.5841	2.53E-16	1.48E-15	yes	down	mRNA
VIBR0546_00745	VIBR0546_00745	malate dehydrogenase	6.988	2.804977	2.72E-16	1.59E-15	yes	up	mRNA
VIBR0546_12657	VIBR0546_12657	hypothetical protein	9.708	3.279167	2.74E-16	1.6E-15	yes	up	mRNA
VIBR0546_12282	VIBR0546_12282	Transcriptional regulator	0.167	−2.58479	2.82E-16	1.65E-15	yes	down	mRNA
VIBR0546_11632	VIBR0546_11632	acetoacetyl-CoA reductase	0.082	−3.60714	2.9E-16	1.69E-15	yes	down	mRNA
VIBR0546_07147	VIBR0546_07147	hypothetical protein	106.553	6.735431	3.24E-16	1.89E-15	yes	up	mRNA
VIBR0546_19162	VIBR0546_19162	putative two-component sensor	4.226	2.079326	3.36E-16	1.95E-15	yes	up	mRNA
VIBR0546_00465	VIBR0546_00465	hypothetical protein	6.859	2.778079	3.71E-16	2.15E-15	yes	up	mRNA
VIBR0546_06077	VIBR0546_06077	acetate kinase	0.213	−2.2344	4.11E-16	2.37E-15	yes	down	mRNA
VIBR0546_16271	VIBR0546_16271	lipid A biosynthesis lauroyl acyltransferase	4.657	2.219303	4.56E-16	2.63E-15	yes	up	mRNA
VIBR0546_17198	VIBR0546_17198	GCN5-like N-acetyltransferase	0.218	−2.19737	5.03E-16	2.9E-15	yes	down	mRNA
VIBR0546_01491	VIBR0546_01491	hypothetical protein	0.126	−2.98996	5.19E-16	2.98E-15	yes	down	mRNA
VIBR0546_20705	VIBR0546_20705	hypothetical protein	0.15	−2.73994	5.42E-16	3.11E-15	yes	down	mRNA
VIBR0546_07412	VIBR0546_07412	hypothetical protein	0.166	−2.59191	5.78E-16	3.31E-15	yes	down	mRNA
VIBR0546_02324	VIBR0546_02324	C4-dicarboxylate transport sensor protein	4.99	2.319022	5.88E-16	3.37E-15	yes	up	mRNA
VIBR0546_14565	VIBR0546_14565	hypothetical protein	0.096	−3.37706	5.92E-16	3.38E-15	yes	down	mRNA
VIBR0546_08812	VIBR0546_08812	argininosuccinate synthase	0.06	−4.04809	6.02E-16	3.44E-15	yes	down	mRNA
VIBR0546_08787	VIBR0546_08787	transcriptional regulator	4.557	2.188078	6.38E-16	3.64E-15	yes	up	mRNA
VIBR0546_03625	VIBR0546_03625	hypothetical protein	0.089	−3.49534	6.61E-16	3.77E-15	yes	down	mRNA

VIBR0546_03335	VIBR0546_03335	lysine-arginine-ornithine-binding periplasmic protein	0.15	−2.73506	6.8E-16	3.87E-15	yes	down	mRNA
VIBR0546_01074	VIBR0546_01074	3-deoxy-D-manno-octulosonate 8-phosphate phosphatase	4.144	2.05102	7.48E-16	4.25E-15	yes	up	mRNA
VIBR0546_01981	VIBR0546_01981	Thioredoxin 2	0.038	−4.73213	7.69E-16	4.36E-15	yes	down	mRNA
VIBR0546_09112	VIBR0546_09112	hypothetical protein	7.067	2.821188	7.98E-16	4.52E-15	yes	up	mRNA
VIBR0546_09127	VIBR0546_09127	putative outer membrane receptor protein	7.404	2.888297	8.48E-16	4.8E-15	yes	up	mRNA
VIBR0546_14812	VIBR0546_14812	hypothetical protein	6.72	2.748419	9.28E-16	5.24E-15	yes	up	mRNA
VIBR0546_03665	VIBR0546_03665	malate synthase	0.097	−3.3699	9.71E-16	5.47E-15	yes	down	mRNA
VIBR0546_14190	VIBR0546_14190	methyl-accepting chemotaxis protein	0.097	−3.37301	1.02E-15	5.75E-15	yes	down	mRNA
VIBR0546_00989	VIBR0546_00989	membrane protein	4.584	2.196543	1.04E-15	5.84E-15	yes	up	mRNA
VIBR0546_03780	VIBR0546_03780	glycine cleavage system protein H	0.174	−2.52242	1.17E-15	6.56E-15	yes	down	mRNA
VIBR0546_06017	VIBR0546_06017	transcriptional regulator	7.329	2.873662	1.19E-15	6.66E-15	yes	up	mRNA
VIBR0546_20675	VIBR0546_20675	hypothetical protein	0.109	−3.20196	1.25E-15	6.95E-15	yes	down	mRNA
VIBR0546_16611	VIBR0546_16611	hypothetical protein	5.273	2.398761	1.27E-15	7.06E-15	yes	up	mRNA
VIBR0546_09919	VIBR0546_09919	putative transcriptional regulator	0.014	−6.15314	1.34E-15	7.43E-15	yes	down	mRNA
VIBR0546_08279	VIBR0546_08279	potassium uptake protein TrkH	8.127	3.022755	1.45E-15	8.06E-15	yes	up	mRNA
VIBR0546_08525	VIBR0546_08525	hypothetical protein	4.577	2.194482	1.52E-15	8.43E-15	yes	up	mRNA
VIBR0546_18026	VIBR0546_18026	hypothetical protein	0.063	−3.98726	1.57E-15	8.68E-15	yes	down	mRNA
VIBR0546_00205	VIBR0546_00205	OmpA/MotB domain-containing protein	5.066	2.340754	1.63E-15	8.96E-15	yes	up	mRNA
VIBR0546_10949	VIBR0546_10949	electron transport complex protein RnfG	4.082	2.029231	1.65E-15	9.06E-15	yes	up	mRNA
VIBR0546_12382	VIBR0546_12382	hypothetical protein	0.007	−7.09046	1.76E-15	9.68E-15	yes	down	mRNA
VIBR0546_05269	VIBR0546_05269	undecaprenyl pyrophosphate phosphatase	4.836	2.273908	1.89E-15	1.03E-14	yes	up	mRNA
VIBR0546_03225	VIBR0546_03225	ABC-type transport system involved in lipoprotein release permease component	0.118	−3.08469	1.97E-15	1.07E-14	yes	down	mRNA
VIBR0546_09082	VIBR0546_09082	hypothetical protein	15.773	3.979359	2E-15	1.09E-14	yes	up	mRNA
VIBR0546_16713	VIBR0546_16713	omega amino acid--pyruvate transaminase	9.647	3.270121	2.5E-15	1.36E-14	yes	up	mRNA
VIBR0546_18712	VIBR0546_18712	hypothetical protein	0.183	−2.44977	2.54E-15	1.37E-14	yes	down	mRNA
VIBR0546_13900	VIBR0546_13900	GCN5-related N-acetyltransferase	0.173	−2.53209	2.8E-15	1.51E-14	yes	down	mRNA
VIBR0546_12767	VIBR0546_12767	hypothetical protein	0.229	−2.12843	2.97E-15	1.6E-14	yes	down	mRNA
VIBR0546_01486	VIBR0546_01486	hypothetical protein	0.069	−3.85035	3.03E-15	1.63E-14	yes	down	mRNA
VIBR0546_05384	VIBR0546_05384	methylation site containing protein	13.243	3.727118	3.3E-15	1.77E-14	yes	up	mRNA
VIBR0546_00270	VIBR0546_00270	universal stress protein UspE	0.221	−2.17899	3.5E-15	1.87E-14	yes	down	mRNA
VIBR0546_15491	VIBR0546_15491	thioredoxin reductase	0.146	−2.77853	4.02E-15	2.14E-14	yes	down	mRNA
VIBR0546_12117	VIBR0546_12117	oligopeptide ABC transporter periplasmic oligopeptide-binding protein	0.175	−2.51155	4.23E-15	2.25E-14	yes	down	mRNA
VIBR0546_19669	VIBR0546_19669	amino-acid ABC transporter binding protein	0.191	−2.38981	4.48E-15	2.38E-14	yes	down	mRNA
VIBR0546_20353	VIBR0546_20353	peptide ABC transporter%2C permease component	6.235	2.640497	4.5E-15	2.39E-14	yes	up	mRNA
VIBR0546_20378	VIBR0546_20378	glutathione reductase	0.232	−2.10666	4.53E-15	2.4E-14	yes	down	mRNA
VIBR0546_04122	VIBR0546_04122	hypothetical protein	0.19	−2.39636	4.54E-15	2.4E-14	yes	down	mRNA

VIBR0546_08069	VIBR0546_08069	DNA polymerase III subunit psi	4.134	2.047698	4.65E-15	2.46E-14	yes	up	mRNA
VIBR0546_01671	VIBR0546_01671	iron-containing alcohol dehydrogenase	0.178	−2.48629	4.65E-15	2.46E-14	yes	down	mRNA
VIBR0546_11512	VIBR0546_11512	response regulator	0.179	−2.48077	4.74E-15	2.5E-14	yes	down	mRNA
VIBR0546_17313	VIBR0546_17313	TRAP dicarboxylate transporter subunit DctP	0.148	−2.75262	4.8E-15	2.53E-14	yes	down	mRNA
VIBR0546_19934	VIBR0546_19934	hypothetical protein	0.211	−2.2428	5.99E-15	3.14E-14	yes	down	mRNA
VIBR0546_10979	VIBR0546_10979	ribonuclease T	5.3	2.406002	6.12E-15	3.2E-14	yes	up	mRNA
VIBR0546_07507	VIBR0546_07507	hypothetical protein	5.036	2.332344	6.46E-15	3.38E-14	yes	up	mRNA
VIBR0546_10374	VIBR0546_10374	peptidase S8/S53 subtilisin kexin sedolisin	0.051	−4.29684	6.64E-15	3.46E-14	yes	down	mRNA
VIBR0546_18907	VIBR0546_18907	hypothetical protein	4.686	2.228284	7.91E-15	4.12E-14	yes	up	mRNA
VIBR0546_12042	VIBR0546_12042	proton/glutamate symporter	0.23	−2.12277	8.13E-15	4.23E-14	yes	down	mRNA
VIBR0546_13570	VIBR0546_13570	NAD(P)H-nitrite reductase	0.191	−2.38582	8.51E-15	4.42E-14	yes	down	mRNA
VIBR0546_16813	VIBR0546_16813	catalase/hydroperoxidase HPI(I)	0.148	−2.75715	8.82E-15	4.57E-14	yes	down	mRNA
VIBR0546_09207	VIBR0546_09207	SpoOM-related protein	0.214	−2.22605	9.89E-15	5.12E-14	yes	down	mRNA
VIBR0546_12407	VIBR0546_12407	hypothetical protein	4.94	2.304425	1.01E-14	5.24E-14	yes	up	mRNA
VIBR0546_07327	potG	putrescine transporter ATP-binding subunit	0.157	−2.67297	1.04E-14	5.35E-14	yes	down	mRNA
VIBR0546_03040	VIBR0546_03040	membrane associated GGDEF protein	0.207	−2.27018	1.07E-14	5.5E-14	yes	down	mRNA
VIBR0546_14010	VIBR0546_14010	MerR family transcriptional regulator	0.124	−3.00729	1.08E-14	5.54E-14	yes	down	mRNA
VIBR0546_14110	VIBR0546_14110	hypothetical protein	0.13	−2.94029	1.1E-14	5.64E-14	yes	down	mRNA
VIBR0546_05558	VIBR0546_05558	Maf-like protein	4.411	2.141263	1.11E-14	5.71E-14	yes	up	mRNA
VIBR0546_11193	VIBR0546_11193	sulfur transfer complex subunit TusD	5.281	2.400809	1.18E-14	6.04E-14	yes	up	mRNA
VIBR0546_15486	VIBR0546_15486	hypothetical protein	0.183	−2.45103	1.19E-14	6.08E-14	yes	down	mRNA
VIBR0546_04579	VIBR0546_04579	hypothetical protein	6.498	2.700071	1.21E-14	6.18E-14	yes	up	mRNA
VIBR0546_16456	VIBR0546_16456	CapK related-protein	4.887	2.289011	1.33E-14	6.81E-14	yes	up	mRNA
VIBR0546_19724	metN	DL-methionine transporter ATP-binding subunit	6.191	2.63019	1.39E-14	7.06E-14	yes	up	mRNA
VIBR0546_18031	VIBR0546_18031	hypothetical protein	0.177	−2.49582	1.43E-14	7.27E-14	yes	down	mRNA
VIBR0546_06112	VIBR0546_06112	ABC transporter: Transmembrane and ATP-binding protein	0.21	−2.25092	1.44E-14	7.33E-14	yes	down	mRNA
VIBR0546_00655	VIBR0546_00655	methyl-accepting chemotaxis protein	4.398	2.136868	1.67E-14	8.5E-14	yes	up	mRNA
VIBR0546_04037	tnaA	tryptophanase/L-cysteine desulfhydrase%2C PLP-dependent	0.138	−2.8527	1.73E-14	8.75E-14	yes	down	mRNA
VIBR0546_01341	VIBR0546_01341	branched-chain amino acid transport system II carrier protein	5.195	2.377041	1.95E-14	9.88E-14	yes	up	mRNA
VIBR0546_19544	VIBR0546_19544	hypothetical protein	4.961	2.310647	2.04E-14	1.03E-13	yes	up	mRNA
VIBR0546_16668	VIBR0546_16668	type IV pilus assembly protein PilQ	7.652	2.935924	2.07E-14	1.04E-13	yes	up	mRNA
VIBR0546_19464	VIBR0546_19464	cysteine desulfurase	4.608	2.204225	2.44E-14	1.22E-13	yes	up	mRNA
VIBR0546_07462	VIBR0546_07462	zinc ABC transporter permease protein ZnuB	5.328	2.413552	2.94E-14	1.47E-13	yes	up	mRNA
VIBR0546_04809	VIBR0546_04809	hypothetical protein	5.187	2.374905	3.17E-14	1.58E-13	yes	up	mRNA
VIBR0546_06762	VIBR0546_06762	short-chain dehydrogenase/reductase SDR	0.232	−2.10491	3.63E-14	1.8E-13	yes	down	mRNA
VIBR0546_05713	VIBR0546_05713	succinylglutamate desuccinylase/aspartoacylase family protein	0.103	−3.28557	3.65E-14	1.82E-13	yes	down	mRNA
VIBR0546_00455	VIBR0546_00455	hypothetical protein	4.662	2.220996	3.82E-14	1.9E-13	yes	up	mRNA

VIBR0546_18657	VIBR0546_18657	flap endonuclease-like protein	0.228	−2.13166	3.84E-14	1.9E-13	yes	down	mRNA
VIBR0546_07407	VIBR0546_07407	Multidrug resistance protein	0.171	−2.54443	3.93E-14	1.95E-13	yes	down	mRNA
VIBR0546_10814	VIBR0546_10814	CDP-diacylglycerol--glycerol-3-phosphate 3-phosphatidyl-transferase	4.415	2.142402	4.24E-14	2.09E-13	yes	up	mRNA
VIBR0546_01496	VIBR0546_01496	hypothetical protein	0.182	−2.45461	4.83E-14	2.38E-13	yes	down	mRNA
VIBR0546_03830	VIBR0546_03830	D-ribose transporter subunit RbsB	0.126	−2.98902	4.87E-14	2.4E-13	yes	down	mRNA
VIBR0546_05902	VIBR0546_05902	arsenite transporter	0.122	−3.03912	4.91E-14	2.41E-13	yes	down	mRNA
VIBR0546_13402	VIBR0546_13402	general secretion pathway protein J	4.707	2.234837	5.3E-14	2.6E-13	yes	up	mRNA
VIBR0546_07287	VIBR0546_07287	hypothetical protein	0.246	−2.02351	5.39E-14	2.64E-13	yes	down	mRNA
VIBR0546_10754	VIBR0546_10754	hypothetical protein	13.435	3.747873	5.74E-14	2.81E-13	yes	up	mRNA
VIBR0546_12022	VIBR0546_12022	Na+/H+ antiporter NhaD type	0.131	−2.93122	6.19E-14	3.02E-13	yes	down	mRNA
VIBR0546_14887	VIBR0546_14887	para-aminobenzoate synthase component II	4.237	2.08295	6.37E-14	3.1E-13	yes	up	mRNA
VIBR0546_08339	VIBR0546_08339	lysine 2%2C3-aminomutase	4.052	2.018571	6.38E-14	3.11E-13	yes	up	mRNA
VIBR0546_19904	frr	ribosome recycling factor	0.229	−2.12907	7.28E-14	3.54E-13	yes	down	mRNA
VIBR0546_11412	VIBR0546_11412	hypothetical protein	0.212	−2.23862	7.87E-14	3.82E-13	yes	down	mRNA
VIBR0546_02479	VIBR0546_02479	phage integrase family protein	10.082	3.333687	8.27E-14	4E-13	yes	up	mRNA
VIBR0546_19122	VIBR0546_19122	hypothetical protein	0.218	−2.19899	9.69E-14	4.67E-13	yes	down	mRNA
VIBR0546_16581	pheT	phenylalanyl-tRNA synthetase subunit beta	4.165	2.058345	1.02E-13	4.91E-13	yes	up	mRNA
VIBR0546_13795	VIBR0546_13795	transcriptional regulator	0.211	−2.24161	1.08E-13	5.2E-13	yes	down	mRNA
VIBR0546_12337	VIBR0546_12337	Hypothetical dipeptidase	0.098	−3.35655	1.14E-13	5.47E-13	yes	down	mRNA
VIBR0546_00979	VIBR0546_00979	phospho-2-dehydro-3-deoxyheptonate aldolase	4.87	2.283903	1.31E-13	6.25E-13	yes	up	mRNA
VIBR0546_02299	VIBR0546_02299	DNA-binding protein HU-beta	0.173	−2.52808	1.38E-13	6.58E-13	yes	down	mRNA
VIBR0546_16903	VIBR0546_16903	hypothetical protein	4.458	2.156345	1.45E-13	6.89E-13	yes	up	mRNA
VIBR0546_11612	VIBR0546_11612	aspartate racemase	0.247	−2.01782	1.53E-13	7.26E-13	yes	down	mRNA
VIBR0546_10449	VIBR0546_10449	transcriptional regulator	4.116	2.041413	1.57E-13	7.41E-13	yes	up	mRNA
VIBR0546_21350	VIBR0546_21350	glyoxalase/bleomycin resistance protein/dioxygenase	0.099	−3.34081	1.61E-13	7.62E-13	yes	down	mRNA
VIBR0546_01926	VIBR0546_01926	methyl-accepting chemotaxis protein	0.188	−2.40765	1.64E-13	7.75E-13	yes	down	mRNA
VIBR0546_07392	VIBR0546_07392	hypothetical protein	4.435	2.148872	1.67E-13	7.86E-13	yes	up	mRNA
VIBR0546_00944	VIBR0546_00944	putative carbon starvation protein A	7.047	2.816916	1.74E-13	8.16E-13	yes	up	mRNA
VIBR0546_14620	VIBR0546_14620	maltose/maltodextrin transporter ATP-binding protein	0.245	−2.02632	1.75E-13	8.23E-13	yes	down	mRNA
VIBR0546_08627	VIBR0546_08627	signal transduction protein	4.059	2.021251	1.81E-13	8.51E-13	yes	up	mRNA
VIBR0546_02735	VIBR0546_02735	6-phosphogluconate dehydrogenase	0.157	−2.66993	1.85E-13	8.64E-13	yes	down	mRNA
VIBR0546_17203	VIBR0546_17203	glyoxalase/bleomycin resistance protein/dioxygenase	0.199	−2.32562	1.85E-13	8.64E-13	yes	down	mRNA
VIBR0546_09484	VIBR0546_09484	outer membrane protein OmpA	0.232	−2.10993	1.9E-13	8.88E-13	yes	down	mRNA
VIBR0546_11138	mutL	DNA mismatch repair protein	5.522	2.465224	2.34E-13	1.09E-12	yes	up	mRNA
VIBR0546_01571	VIBR0546_01571	hypothetical protein	0.152	−2.71499	2.39E-13	1.11E-12	yes	down	mRNA
VIBR0546_15336	VIBR0546_15336	fumarate hydratase%2C class I	0.175	−2.51611	2.53E-13	1.17E-12	yes	down	mRNA
VIBR0546_03355	VIBR0546_03355	N-acetylglucosamine-6-phosphate deacetylase	0.218	−2.20032	2.83E-13	1.31E-12	yes	down	mRNA

VIBR0546_12582	VIBR0546_12582	hypothetical protein	10.665	3.414879	2.86E-13	1.32E-12	yes	up	mRNA
VIBR0546_04382	VIBR0546_04382	3-oxoacyl-(acyl carrier protein) synthase III	4.063	2.02255	2.89E-13	1.33E-12	yes	up	mRNA
VIBR0546_02990	VIBR0546_02990	transcriptional regulator	0.127	−2.97423	2.94E-13	1.35E-12	yes	down	mRNA
VIBR0546_07337	VIBR0546_07337	binding-protein-dependent transport systems inner membrane component	0.111	−3.16981	3.22E-13	1.47E-12	yes	down	mRNA
VIBR0546_09714	holA	DNA polymerase III subunit delta	5.677	2.505017	3.34E-13	1.53E-12	yes	up	mRNA
VIBR0546_14832	VIBR0546_14832	aspartate carbamoyltransferase regulatory subunit	0.104	−3.26882	3.43E-13	1.57E-12	yes	down	mRNA
VIBR0546_11332	VIBR0546_11332	periplasmic nitrate reductase%2C cytochrome c-type protein	0.137	−2.86858	3.49E-13	1.59E-12	yes	down	mRNA
VIBR0546_00730	VIBR0546_00730	histidine kinase	9.72	3.280921	3.64E-13	1.66E-12	yes	up	mRNA
VIBR0546_09102	VIBR0546_09102	hypothetical protein	7.787	2.961059	3.65E-13	1.67E-12	yes	up	mRNA
VIBR0546_00929	lspA	lipoprotein signal peptidase	5.871	2.553508	4.06E-13	1.85E-12	yes	up	mRNA
VIBR0546_01561	pntB	pyridine nucleotide transhydrogenase	0.189	−2.40533	4.1E-13	1.86E-12	yes	down	mRNA
VIBR0546_10114	VIBR0546_10114	putative amino acid ABC transporter%2C periplasmic amino acid-binding protein	0.2	−2.32507	4.18E-13	1.9E-12	yes	down	mRNA
VIBR0546_12697	VIBR0546_12697	hypothetical protein	23.184	4.53508	4.32E-13	1.95E-12	yes	up	mRNA
VIBR0546_11822	VIBR0546_11822	putative aldehyde dehydrogenase	0.205	−2.28882	4.41E-13	1.99E-12	yes	down	mRNA
VIBR0546_11627	VIBR0546_11627	putative acyltransferase	0.098	−3.3499	4.45E-13	2.01E-12	yes	down	mRNA
VIBR0546_09734	VIBR0546_09734	rod shape-determining protein RodA	4.172	2.060607	4.57E-13	2.06E-12	yes	up	mRNA
VIBR0546_10209	VIBR0546_10209	hypothetical protein	0.152	−2.71449	4.98E-13	2.24E-12	yes	down	mRNA
VIBR0546_04107	VIBR0546_04107	hypothetical protein	0.194	−2.36241	5.51E-13	2.47E-12	yes	down	mRNA
VIBR0546_03785	VIBR0546_03785	serine hydroxymethyltransferase	0.151	−2.72317	5.64E-13	2.53E-12	yes	down	mRNA
VIBR0546_08642	VIBR0546_08642	hypothetical protein	0.042	−4.56805	6.19E-13	2.76E-12	yes	down	mRNA
VIBR0546_02056	VIBR0546_02056	hypothetical protein	4.559	2.188791	7.32E-13	3.26E-12	yes	up	mRNA
VIBR0546_07272	VIBR0546_07272	hypothetical protein	0.226	−2.14701	7.7E-13	3.42E-12	yes	down	mRNA
VIBR0546_04719	VIBR0546_04719	GGDEF domain-containing protein	5.951	2.573224	8.28E-13	3.67E-12	yes	up	mRNA
VIBR0546_13910	VIBR0546_13910	hypothetical protein	0.047	−4.41713	8.64E-13	3.83E-12	yes	down	mRNA
VIBR0546_00320	VIBR0546_00320	GGDEF family protein	0.229	−2.124	8.86E-13	3.92E-12	yes	down	mRNA
VIBR0546_03210	VIBR0546_03210	autolysin sensor kinase	0.244	−2.03633	9.1E-13	4.02E-12	yes	down	mRNA
VIBR0546_13252	VIBR0546_13252	putative hydrolase	6.284	2.65168	9.2E-13	4.06E-12	yes	up	mRNA
VIBR0546_14225	VIBR0546_14225	methyl-accepting chemotaxis protein	0.189	−2.40343	9.62E-13	4.24E-12	yes	down	mRNA
VIBR0546_00020	VIBR0546_00020	acylphosphatase	0.243	−2.03813	9.85E-13	4.33E-12	yes	down	mRNA
VIBR0546_01104	VIBR0546_01104	hypothetical protein	7.1	2.827816	9.92E-13	4.36E-12	yes	up	mRNA
VIBR0546_11767	VIBR0546_11767	hypothetical protein	0.205	−2.28637	1.04E-12	4.56E-12	yes	down	mRNA
VIBR0546_20358	VIBR0546_20358	peptide ABC transporter permease component	5.076	2.343578	1.08E-12	4.74E-12	yes	up	mRNA
VIBR0546_14020	VIBR0546_14020	hypothetical protein	0.178	−2.49024	1.09E-12	4.77E-12	yes	down	mRNA
VIBR0546_21060	VIBR0546_21060	biotin synthesis protein BioC	8.724	3.125028	1.09E-12	4.79E-12	yes	up	mRNA
VIBR0546_01746	VIBR0546_01746	putative fatty acid desaturase	0.191	−2.39074	1.1E-12	4.8E-12	yes	down	mRNA
VIBR0546_03035	VIBR0546_03035	hypothetical protein	0.133	−2.9062	1.11E-12	4.83E-12	yes	down	mRNA

VIBR0546_06617	VIBR0546_06617	proline iminopeptidase	8.32	3.056587	1.24E-12	5.38E-12	yes	up	mRNA
VIBR0546_07152	VIBR0546_07152	hypothetical protein	5.088	2.34697	1.25E-12	5.43E-12	yes	up	mRNA
VIBR0546_02081	VIBR0546_02081	adenosylcobinamide-phosphate synthase	4.462	2.157548	1.63E-12	7.05E-12	yes	up	mRNA
VIBR0546_13640	VIBR0546_13640	hypothetical protein	0.215	−2.21778	1.8E-12	7.78E-12	yes	down	mRNA
VIBR0546_20910	VIBR0546_20910	putative dehydrogenase	4.102	2.036367	2.21E-12	9.51E-12	yes	up	mRNA
VIBR0546_07082	VIBR0546_07082	hypothetical protein	4.486	2.16532	2.23E-12	9.58E-12	yes	up	mRNA
VIBR0546_00565	VIBR0546_00565	acetyltransferase	0.052	−4.2645	2.27E-12	9.75E-12	yes	down	mRNA
VIBR0546_17093	VIBR0546_17093	putative transport protein of outer membrane lipoproteins	0.179	−2.48215	3.02E-12	1.29E-11	yes	down	mRNA
VIBR0546_04172	VIBR0546_04172	hypothetical protein	7.165	2.840926	3.12E-12	1.33E-11	yes	up	mRNA
VIBR0546_11872	VIBR0546_11872	arginine decarboxylase	0.06	−4.06571	3.22E-12	1.37E-11	yes	down	mRNA
VIBR0546_14590	VIBR0546_14590	twin-arginine translocation pathway signal	0.239	−2.06221	3.3E-12	1.4E-11	yes	down	mRNA
VIBR0546_10094	VIBR0546_10094	hypothetical protein	0.133	−2.90823	3.31E-12	1.4E-11	yes	down	mRNA
VIBR0546_08274	VIBR0546_08274	hypothetical protein	5.212	2.381842	3.34E-12	1.41E-11	yes	up	mRNA
VIBR0546_20388	VIBR0546_20388	oligopeptidase A	0.244	−2.03281	3.55E-12	1.5E-11	yes	down	mRNA
VIBR0546_16231	VIBR0546_16231	hypothetical protein	4.413	2.141814	3.69E-12	1.56E-11	yes	up	mRNA
VIBR0546_14415	VIBR0546_14415	dihydroorotase	0.194	−2.36438	3.78E-12	1.59E-11	yes	down	mRNA
VIBR0546_00735	VIBR0546_00735	transcriptional regulatory protein CpxR	18.938	4.24323	4.07E-12	1.71E-11	yes	up	mRNA
VIBR0546_03205	VIBR0546_03205	putative response regulator	0.199	−2.33161	4.91E-12	2.06E-11	yes	down	mRNA
VIBR0546_05344	rplS	50S ribosomal protein L19	0.148	−2.75852	4.92E-12	2.07E-11	yes	down	mRNA
VIBR0546_03705	VIBR0546_03705	putative ATP-dependent RNA helicase RhlE	0.171	−2.54645	4.95E-12	2.07E-11	yes	down	mRNA
VIBR0546_19729	VIBR0546_19729	ABC transporter%2C permease protein	4.065	2.023096	5.1E-12	2.13E-11	yes	up	mRNA
VIBR0546_07732	VIBR0546_07732	hypothetical protein	4.667	2.222598	5.29E-12	2.21E-11	yes	up	mRNA
VIBR0546_08997	VIBR0546_08997	hypothetical protein	5.932	2.568625	5.54E-12	2.31E-11	yes	up	mRNA
VIBR0546_13517	VIBR0546_13517	hypothetical protein	4.583	2.196395	5.62E-12	2.34E-11	yes	up	mRNA
VIBR0546_06837	VIBR0546_06837	hypothetical protein	0.142	−2.81098	5.73E-12	2.38E-11	yes	down	mRNA
VIBR0546_03505	VIBR0546_03505	hypothetical protein	0.062	−4.01891	5.85E-12	2.43E-11	yes	down	mRNA
VIBR0546_11307	VIBR0546_11307	response regulator	0.221	−2.17999	6.05E-12	2.51E-11	yes	down	mRNA
VIBR0546_10279	VIBR0546_10279	hypothetical protein	0.151	−2.72633	6.11E-12	2.53E-11	yes	down	mRNA
VIBR0546_07854	VIBR0546_07854	DnaK suppressor protein	0.218	−2.19645	6.26E-12	2.59E-11	yes	down	mRNA
VIBR0546_10149	VIBR0546_10149	hypothetical protein	9.361	3.226653	6.51E-12	2.69E-11	yes	up	mRNA
VIBR0546_01636	VIBR0546_01636	bifunctional PTS system fructose-specific transporter subunit IIA/HPr protein	0.131	−2.93313	6.78E-12	2.79E-11	yes	down	mRNA
VIBR0546_06332	VIBR0546_06332	type VI secretion system protein ImpG	8.531	3.092659	6.95E-12	2.86E-11	yes	up	mRNA
VIBR0546_03825	rbsC	ribose ABC transporter permease protein	0.19	−2.39325	7.09E-12	2.91E-11	yes	down	mRNA
VIBR0546_16928	smpB	SsrA-binding protein	0.193	−2.37636	7.28E-12	2.98E-11	yes	down	mRNA
VIBR0546_16246	VIBR0546_16246	hypothetical protein	5.828	2.543043	7.32E-12	3E-11	yes	up	mRNA
VIBR0546_16626	VIBR0546_16626	Hypothetical dipeptidase	0.171	−2.54794	7.62E-12	3.11E-11	yes	down	mRNA

VIBR0546_17653	VIBR0546_17653	putative amino acid ABC transporter%2C periplasmic amino acid-binding protein	0.214	−2.22211	7.73E-12	3.16E-11	yes	down	mRNA
VIBR0546_20775	murD	UDP-N-acetylmuramoyl-L-alanyl-D-glutamate synthetase	4.061	2.021826	9.92E-12	4.01E-11	yes	up	mRNA
VIBR0546_21390	VIBR0546_21390	hypothetical protein	0.195	−2.35991	9.99E-12	4.03E-11	yes	down	mRNA
VIBR0546_12572	VIBR0546_12572	high-affinity zinc uptake system membrane protein ZnuB	10.104	3.336814	1.06E-11	4.26E-11	yes	up	mRNA
VIBR0546_13257	VIBR0546_13257	hypothetical protein	5.853	2.549275	1.12E-11	4.51E-11	yes	up	mRNA
VIBR0546_11867	VIBR0546_11867	agmatinase	0.106	−3.23321	1.15E-11	4.61E-11	yes	down	mRNA
VIBR0546_13002	VIBR0546_13002	hypothetical protein	8.408	3.071704	1.33E-11	5.32E-11	yes	up	mRNA
VIBR0546_05379	VIBR0546_05379	type IV pilus assembly protein PilB	7.689	2.942832	1.46E-11	5.86E-11	yes	up	mRNA
VIBR0546_20555	sucC	succinyl-CoA synthetase subunit beta	0.208	−2.26295	1.53E-11	6.09E-11	yes	down	mRNA
VIBR0546_21770	VIBR0546_21770	uridine/cytidine kinase	0.219	−2.19279	1.59E-11	6.32E-11	yes	down	mRNA
VIBR0546_02890	VIBR0546_02890	putative oxidoreductase	0.211	−2.2432	1.62E-11	6.45E-11	yes	down	mRNA
VIBR0546_02985	VIBR0546_02985	Outer membrane protein	0.101	−3.31356	1.62E-11	6.45E-11	yes	down	mRNA
VIBR0546_19037	VIBR0546_19037	permease	8.516	3.090195	1.81E-11	7.18E-11	yes	up	mRNA
VIBR0546_18611	VIBR0546_18611	O-acetylhomoserine sulfhydrylase/O-succinylhomoserine sulfhydrylase	0.136	−2.87353	1.86E-11	7.38E-11	yes	down	mRNA
VIBR0546_15696	VIBR0546_15696	transcriptional regulator	4.258	2.090282	1.86E-11	7.4E-11	yes	up	mRNA
VIBR0546_10579	VIBR0546_10579	universal stress protein UspB	0.249	−2.00485	1.88E-11	7.47E-11	yes	down	mRNA
VIBR0546_19297	VIBR0546_19297	malate dehydrogenase	0.175	−2.51275	2.32E-11	9.16E-11	yes	down	mRNA
VIBR0546_10029	VIBR0546_10029	protein-glutamate methylesterase CheB	0.071	−3.80649	2.38E-11	9.39E-11	yes	down	mRNA
VIBR0546_00260	VIBR0546_00260	membrane protein	4.706	2.234513	2.62E-11	1.03E-10	yes	up	mRNA
VIBR0546_09854	VIBR0546_09854	hypothetical protein	0.136	−2.87968	3.06E-11	1.2E-10	yes	down	mRNA
VIBR0546_13710	VIBR0546_13710	dehydrogenase	0.15	−2.73933	3.12E-11	1.22E-10	yes	down	mRNA
VIBR0546_18496	VIBR0546_18496	hypothetical protein	4.548	2.18516	3.33E-11	1.29E-10	yes	up	mRNA
VIBR0546_17278	VIBR0546_17278	Aldose 1-epimerase	0.202	−2.30864	3.33E-11	1.29E-10	yes	down	mRNA
VIBR0546_13985	VIBR0546_13985	hypothetical protein	0.195	−2.36071	3.34E-11	1.3E-10	yes	down	mRNA
VIBR0546_06982	VIBR0546_06982	bifunctional proline dehydrogenase/pyrroline-5-carboxylate dehydrogenase	0.202	−2.31033	3.48E-11	1.35E-10	yes	down	mRNA
VIBR0546_02685	tdh	L-threonine 3-dehydrogenase	0.225	−2.15123	3.7E-11	1.43E-10	yes	down	mRNA
VIBR0546_18927	VIBR0546_18927	hypothetical protein	6.357	2.668388	3.83E-11	1.48E-10	yes	up	mRNA
VIBR0546_03180	malE	maltose ABC transporter periplasmic protein	0.152	−2.72232	4.16E-11	1.6E-10	yes	down	mRNA
VIBR0546_05603	VIBR0546_05603	hypothetical protein	4.588	2.197956	5.4E-11	2.08E-10	yes	up	mRNA
VIBR0546_03795	VIBR0546_03795	glycine cleavage system protein T2	0.206	−2.27965	5.78E-11	2.21E-10	yes	down	mRNA
VIBR0546_12292	VIBR0546_12292	GCN5-like N-acetyltransferase	0.123	−3.02466	5.89E-11	2.26E-10	yes	down	mRNA
VIBR0546_03907	VIBR0546_03907	adenylylsulfate kinase	0.189	−2.402	5.96E-11	2.28E-10	yes	down	mRNA
VIBR0546_19864	fabZ	(3R)-hydroxymyristoyl-ACP dehydratase	5.236	2.3886	6.22E-11	2.37E-10	yes	up	mRNA
VIBR0546_08887	VIBR0546_08887	ABC transporter%2C periplasmic substrate-binding protein	0.152	−2.71327	6.42E-11	2.44E-10	yes	down	mRNA
VIBR0546_10159	VIBR0546_10159	maltodextrin transport system permease MalD	10.821	3.435828	6.91E-11	2.62E-10	yes	up	mRNA

VIBR0546_00650	VIBR0546_00650	nitrite reductase subunit NirD	5.18	2.373063	7.22E-11	2.74E-10	yes	up	mRNA
VIBR0546_15786	grxA	glutaredoxin 1	0.242	−2.047	7.49E-11	2.84E-10	yes	down	mRNA
VIBR0546_13027	VIBR0546_13027	type IV pilus biogenesis/stability protein PilW	17.188	4.103331	7.53E-11	2.85E-10	yes	up	mRNA
VIBR0546_20023	VIBR0546_20023	hypothetical protein	5.014	2.325822	8.47E-11	3.2E-10	yes	up	mRNA
VIBR0546_05553	VIBR0546_05553	rod shape-determining protein MreD	6.148	2.620171	8.53E-11	3.22E-10	yes	up	mRNA
VIBR0546_18501	VIBR0546_18501	sulfur acceptor protein SufE for iron-sulfur cluster assembly	5.609	2.487793	8.88E-11	3.34E-10	yes	up	mRNA
VIBR0546_16106	VIBR0546_16106	chorismate--pyruvate lyase	4.391	2.134556	9.21E-11	3.46E-10	yes	up	mRNA
VIBR0546_08515	VIBR0546_08515	TolR membrane protein	4.052	2.018568	9.22E-11	3.46E-10	yes	up	mRNA
VIBR0546_03045	VIBR0546_03045	transcriptional regulator	0.243	−2.03826	9.23E-11	3.46E-10	yes	down	mRNA
VIBR0546_06172	VIBR0546_06172	putative phospholipase C	0.154	−2.69879	9.38E-11	3.52E-10	yes	down	mRNA
VIBR0546_16718	VIBR0546_16718	transcriptional regulator%2C substrate-binding of LysR family protein	4.907	2.294755	1.01E-10	3.76E-10	yes	up	mRNA
VIBR0546_13635	VIBR0546_13635	transcriptional regulator%2C LysR family protein	0.195	−2.35581	1.08E-10	4.02E-10	yes	down	mRNA
VIBR0546_08692	VIBR0546_08692	hypothetical protein	4.131	2.046665	1.37E-10	5.08E-10	yes	up	mRNA
VIBR0546_14707	VIBR0546_14707	hemolysin	0.191	−2.3882	1.38E-10	5.11E-10	yes	down	mRNA
VIBR0546_17633	VIBR0546_17633	urease accessory protein	0.162	−2.62553	1.56E-10	5.74E-10	yes	down	mRNA
VIBR0546_18106	VIBR0546_18106	ABC transporter extracellular solute-binding protein	0.169	−2.5677	1.68E-10	6.18E-10	yes	down	mRNA
VIBR0546_20685	VIBR0546_20685	hypothetical protein	0.063	−3.99632	1.91E-10	7E-10	yes	down	mRNA
VIBR0546_01596	VIBR0546_01596	hypothetical protein	0.104	−3.26471	1.96E-10	7.18E-10	yes	down	mRNA
VIBR0546_05942	VIBR0546_05942	L-lactate permease	0.209	−2.25637	2.27E-10	8.27E-10	yes	down	mRNA
VIBR0546_11377	VIBR0546_11377	allophanate hydrolase subunit 2	0.157	−2.67232	2.37E-10	8.61E-10	yes	down	mRNA
VIBR0546_06037	VIBR0546_06037	methyl-accepting chemotaxis protein	0.222	−2.16917	2.88E-10	1.05E-09	yes	down	mRNA
VIBR0546_00210	VIBR0546_00210	hypothetical protein	6.728	2.750123	2.95E-10	1.07E-09	yes	up	mRNA
VIBR0546_01173	rpmE	50S ribosomal protein L31	0.221	−2.17816	2.95E-10	1.07E-09	yes	down	mRNA
VIBR0546_21795	VIBR0546_21795	hypothetical protein	5.149	2.364262	3.06E-10	1.11E-09	yes	up	mRNA
VIBR0546_13920	VIBR0546_13920	hypothetical protein	0.196	−2.34853	3.35E-10	1.21E-09	yes	down	mRNA
VIBR0546_14390	VIBR0546_14390	AraC/XylS family transcriptional regulator	0.219	−2.19067	3.57E-10	1.28E-09	yes	down	mRNA
VIBR0546_14837	pyrB	aspartate carbamoyltransferase catalytic subunit	0.222	−2.17401	3.57E-10	1.29E-09	yes	down	mRNA
VIBR0546_18436	VIBR0546_18436	ABC transporter ATP-binding protein	0.212	−2.23923	3.63E-10	1.3E-09	yes	down	mRNA
VIBR0546_03952	VIBR0546_03952	sulfite reductase [NADPH] flavoprotein alpha-component	0.227	−2.1418	4.06E-10	1.45E-09	yes	down	mRNA
VIBR0546_11452	VIBR0546_11452	hypothetical protein	8.797	3.137041	5.2E-10	1.85E-09	yes	up	mRNA
VIBR0546_13560	VIBR0546_13560	signal transduction histidine kinase	6.205	2.633404	5.5E-10	1.94E-09	yes	up	mRNA
VIBR0546_00040	VIBR0546_00040	hypothetical protein	0.175	−2.51632	6.02E-10	2.13E-09	yes	down	mRNA
VIBR0546_09954	VIBR0546_09954	hypothetical protein	0.208	−2.26275	6.15E-10	2.17E-09	yes	down	mRNA
VIBR0546_07899	VIBR0546_07899	hypothetical protein	4.943	2.305281	6.29E-10	2.22E-09	yes	up	mRNA
VIBR0546_15646	VIBR0546_15646	membrane protein	4.86	2.281038	6.4E-10	2.25E-09	yes	up	mRNA
VIBR0546_19202	VIBR0546_19202	PTS system%2C fructose-specific IIABC component	7.184	2.844885	6.41E-10	2.25E-09	yes	up	mRNA
VIBR0546_06992	VIBR0546_06992	sodium/proline symporter	0.111	−3.17055	6.54E-10	2.3E-09	yes	down	mRNA

VIBR0546_06437	VIBR0546_06437	UDP-2%2C3-diacylglucosamine hydrolase	6.667	2.737041	6.69E-10	2.35E-09	yes	up	mRNA
VIBR0546_07772	VIBR0546_07772	hypothetical protein	4.79	2.259898	6.81E-10	2.38E-09	yes	up	mRNA
VIBR0546_15891	VIBR0546_15891	ComEC/rec2 family protein	4.996	2.320711	7.04E-10	2.46E-09	yes	up	mRNA
VIBR0546_14380	VIBR0546_14380	putative hemolysin	0.155	−2.68669	7.51E-10	2.61E-09	yes	down	mRNA
VIBR0546_02586	VIBR0546_02586	putative transporter	4.571	2.192461	8.12E-10	2.82E-09	yes	up	mRNA
VIBR0546_04042	VIBR0546_04042	alkylphosphonate utilization operon protein PhnA	0.113	−3.14719	8.38E-10	2.9E-09	yes	down	mRNA
VIBR0546_03425	VIBR0546_03425	2-hydroxyhepta-2%2C4-diene-1%2C7-dioate isomerase	0.241	−2.05074	8.44E-10	2.92E-09	yes	down	mRNA
VIBR0546_01476	VIBR0546_01476	hypothetical protein	0.237	−2.07802	8.56E-10	2.96E-09	yes	down	mRNA
VIBR0546_02820	VIBR0546_02820	transcriptional regulator BetI	0.152	−2.72149	8.7E-10	3E-09	yes	down	mRNA
VIBR0546_12587	VIBR0546_12587	dihydroorotase	4.793	2.260976	8.88E-10	3.06E-09	yes	up	mRNA
VIBR0546_20605	VIBR0546_20605	hypothetical protein	9.47	3.243403	9.5E-10	3.26E-09	yes	up	mRNA
VIBR0546_09514	VIBR0546_09514	hypothetical protein	0.216	−2.21402	1.23E-09	4.21E-09	yes	down	mRNA
VIBR0546_13675	VIBR0546_13675	putative lipoprotein	0.054	−4.21285	1.32E-09	4.51E-09	yes	down	mRNA
VIBR0546_03685	VIBR0546_03685	NAD-dependent epimerase/dehydratase	0.231	−2.11489	1.41E-09	4.82E-09	yes	down	mRNA
VIBR0546_02449	minC	septum formation inhibitor	4.112	2.039818	1.57E-09	5.33E-09	yes	up	mRNA
VIBR0546_19022	VIBR0546_19022	ATPase of the PP-loop superfamily protein	4.179	2.063122	1.92E-09	6.49E-09	yes	up	mRNA
VIBR0546_17183	VIBR0546_17183	ABC transporter: transmembrane protein	0.142	−2.81244	2.02E-09	6.8E-09	yes	down	mRNA
VIBR0546_05264	VIBR0546_05264	2-amino-4-hydroxy-6-hydroxymethyldihydropteridine pyro-phosphokinase	4.21	2.073831	2.19E-09	7.37E-09	yes	up	mRNA
VIBR0546_10024	VIBR0546_10024	methyl-accepting chemotaxis protein	0.092	−3.44768	2.35E-09	7.87E-09	yes	down	mRNA
VIBR0546_06887	VIBR0546_06887	hypothetical protein	0.197	−2.34584	2.44E-09	8.17E-09	yes	down	mRNA
VIBR0546_00775	VIBR0546_00775	LysR family transcriptional regulator	4.135	2.048036	2.64E-09	8.82E-09	yes	up	mRNA
VIBR0546_13850	phhB	pterin-4-alpha-carbinolamine dehydratase	0.245	−2.03158	2.93E-09	9.74E-09	yes	down	mRNA
VIBR0546_17243	VIBR0546_17243	TRAP dicarboxylate transporter subunit DctP	0.145	−2.78186	2.99E-09	9.92E-09	yes	down	mRNA
VIBR0546_10019	VIBR0546_10019	putative anti-sigma F factor antagonist	0.091	−3.45975	3.04E-09	1.01E-08	yes	down	mRNA
VIBR0546_12457	VIBR0546_12457	maltoporin	4.613	2.20569	3.07E-09	1.02E-08	yes	up	mRNA
VIBR0546_05274	ftsB	cell division protein FtsB	5.365	2.423647	3.12E-09	1.03E-08	yes	up	mRNA
VIBR0546_03600	VIBR0546_03600	hypothetical protein	0.147	−2.76972	3.17E-09	1.05E-08	yes	down	mRNA
VIBR0546_00160	VIBR0546_00160	thiol-disulfide isomerase and thioredoxin	4.693	2.230632	3.24E-09	1.07E-08	yes	up	mRNA
VIBR0546_09819	VIBR0546_09819	glutathione S-transferase-related protein	0.125	−2.99758	3.45E-09	1.14E-08	yes	down	mRNA
VIBR0546_07597	VIBR0546_07597	heme exporter protein D	4.805	2.264501	3.47E-09	1.14E-08	yes	up	mRNA
VIBR0546_00570	VIBR0546_00570	putative glycosyltransferase	0.199	−2.32718	3.9E-09	1.28E-08	yes	down	mRNA
VIBR0546_16943	VIBR0546_16943	hypothetical protein	0.173	−2.53514	3.92E-09	1.29E-08	yes	down	mRNA
VIBR0546_20413	VIBR0546_20413	putative signal peptide protein	0.104	−3.26689	4.03E-09	1.32E-08	yes	down	mRNA
VIBR0546_21415	VIBR0546_21415	enoyl-[acyl-carrier-protein] reductase (FMN)	0.22	−2.18238	4.08E-09	1.34E-08	yes	down	mRNA
VIBR0546_05718	VIBR0546_05718	ribosomal protein S6 modification protein	0.08	−3.63831	4.44E-09	1.45E-08	yes	down	mRNA
VIBR0546_07137	VIBR0546_07137	phosphate ABC transporter%2C permease protein	0.176	−2.5095	4.54E-09	1.48E-08	yes	down	mRNA
VIBR0546_07207	VIBR0546_07207	hypothetical protein	0.105	−3.24877	4.63E-09	1.51E-08	yes	down	mRNA

VIBR0546_10034	VIBR0546_10034	chemoreceptor glutamine deamidase CheD	0.104	−3.2595	5.05E-09	1.64E-08	yes	down	mRNA
VIBR0546_10289	VIBR0546_10289	bifunctional nitric oxide dioxygenase/dihydropteridine reductase 2	0.217	−2.20491	5.32E-09	1.73E-08	yes	down	mRNA
VIBR0546_06482	VIBR0546_06482	hypothetical protein	0.066	−3.92025	5.38E-09	1.75E-08	yes	down	mRNA
VIBR0546_00545	VIBR0546_00545	putative galactosyltransferase	0.238	−2.06904	5.44E-09	1.76E-08	yes	down	mRNA
VIBR0546_13650	VIBR0546_13650	hypothetical protein	4.097	2.034459	5.59E-09	1.81E-08	yes	up	mRNA
VIBR0546_18882	VIBR0546_18882	polar flagellar assembly protein FliO	5.211	2.381604	5.96E-09	1.93E-08	yes	up	mRNA
VIBR0546_19272	VIBR0546_19272	hypothetical protein	4.961	2.310606	6.63E-09	2.14E-08	yes	up	mRNA
VIBR0546_15896	VIBR0546_15896	hypothetical protein	4.285	2.099167	6.77E-09	2.18E-08	yes	up	mRNA
VIBR0546_14180	VIBR0546_14180	anti-anti-sigma regulatory factor	0.158	−2.66395	7.12E-09	2.29E-08	yes	down	mRNA
VIBR0546_17933	VIBR0546_17933	ABC-type hydroxamate-dependent iron transport system%2C ATPase component	6.193	2.630745	7.4E-09	2.36E-08	yes	up	mRNA
VIBR0546_02895	VIBR0546_02895	hypothetical protein	0.177	−2.49492	8.04E-09	2.56E-08	yes	down	mRNA
VIBR0546_12672	VIBR0546_12672	methyltransferase type 11	4.174	2.061554	8.31E-09	2.64E-08	yes	up	mRNA
VIBR0546_12437	VIBR0546_12437	ABC-type dipeptide transport system%2C periplasmic component	7.833	2.969607	8.35E-09	2.65E-08	yes	up	mRNA
VIBR0546_01736	VIBR0546_01736	membrane-fusion protein	4.013	2.004524	9.14E-09	2.89E-08	yes	up	mRNA
VIBR0546_13262	VIBR0546_13262	organic radical activating enzyme	5.134	2.360191	1.05E-08	3.29E-08	yes	up	mRNA
VIBR0546_10389	VIBR0546_10389	endoribonuclease L-PSP domain	11.568	3.532015	1.08E-08	3.39E-08	yes	up	mRNA
VIBR0546_01296	VIBR0546_01296	hypothetical protein	0.218	−2.20013	1.17E-08	3.67E-08	yes	down	mRNA
VIBR0546_05593	VIBR0546_05593	2-dehydropantoate 2-reductase	5.04	2.333463	1.23E-08	3.87E-08	yes	up	mRNA
VIBR0546_06292	VIBR0546_06292	Lysine exporter protein (LYSE/YGGA)	0.1	−3.32764	1.45E-08	4.54E-08	yes	down	mRNA
VIBR0546_16673	VIBR0546_16673	fimbrial assembly protein PilO	9.307	3.218274	1.47E-08	4.58E-08	yes	up	mRNA
VIBR0546_10599	VIBR0546_10599	hypothetical protein	4.708	2.235176	1.55E-08	4.84E-08	yes	up	mRNA
VIBR0546_10324	VIBR0546_10324	hypothetical protein	0.235	−2.08766	1.62E-08	5.03E-08	yes	down	mRNA
VIBR0546_04062	VIBR0546_04062	hypothetical protein	0.104	−3.26435	1.69E-08	5.26E-08	yes	down	mRNA
VIBR0546_13565	VIBR0546_13565	putative nitrite reductase NADPH (small subunit) oxidoreductase protein	0.092	−3.44331	1.74E-08	5.38E-08	yes	down	mRNA
VIBR0546_07607	VIBR0546_07607	cytochrome c-type biogenesis protein CcmF	4.392	2.134922	1.82E-08	5.63E-08	yes	up	mRNA
VIBR0546_01911	VIBR0546_01911	transketolase	0.203	−2.30328	1.86E-08	5.74E-08	yes	down	mRNA
VIBR0546_11019	VIBR0546_11019	hypothetical protein	4.25	2.087612	2.13E-08	6.55E-08	yes	up	mRNA
VIBR0546_19604	VIBR0546_19604	ABC transporter	0.234	−2.09533	2.27E-08	6.94E-08	yes	down	mRNA
VIBR0546_12297	VIBR0546_12297	putative Azoreductase	0.164	−2.60527	2.35E-08	7.18E-08	yes	down	mRNA
VIBR0546_09092	VIBR0546_09092	hypothetical protein	17.588	4.13656	2.37E-08	7.23E-08	yes	up	mRNA
VIBR0546_01986	VIBR0546_01986	hypothetical protein	0.007	−7.17418	2.65E-08	8.05E-08	yes	down	mRNA
VIBR0546_08410	VIBR0546_08410	hypothetical protein	6.31	2.657743	2.76E-08	8.36E-08	yes	up	mRNA
VIBR0546_06117	VIBR0546_06117	ABC transporter: Transmembrane and ATP-binding protein	0.203	−2.30251	2.96E-08	8.94E-08	yes	down	mRNA
VIBR0546_20800	VIBR0546_20800	cell division protein FtsL	5.864	2.551998	3.09E-08	9.33E-08	yes	up	mRNA

VIBR0546_01461	VIBR0546_01461	hypothetical protein	0.153	−2.70417	3.1E-08	9.35E-08	yes	down	mRNA
VIBR0546_19484	VIBR0546_19484	acid phosphatase	21.698	4.439472	3.35E-08	1E-07	yes	up	mRNA
VIBR0546_03520	VIBR0546_03520	heme A synthase cytochrome oxidase biogenesis protein Cox15-CtaA	0.176	−2.50797	3.45E-08	1.03E-07	yes	down	mRNA
VIBR0546_10654	VIBR0546_10654	ThiF protein	4.427	2.146399	3.61E-08	1.08E-07	yes	up	mRNA
VIBR0546_19973	VIBR0546_19973	hypothetical protein	116.468	6.863794	3.75E-08	1.12E-07	yes	up	mRNA
VIBR0546_07712	VIBR0546_07712	putative proteinase inhibitor	5.228	2.386331	3.92E-08	1.17E-07	yes	up	mRNA
VIBR0546_13855	phhA	phenylalanine 4-monooxygenase	0.166	−2.58796	4.03E-08	1.2E-07	yes	down	mRNA
VIBR0546_08104	VIBR0546_08104	lipoprotein-related protein	0.145	−2.78133	4.61E-08	1.36E-07	yes	down	mRNA
VIBR0546_12547	VIBR0546_12547	LysR family transcriptional regulator	4.263	2.092038	4.96E-08	1.46E-07	yes	up	mRNA
VIBR0546_10039	VIBR0546_10039	chemotaxis protein methyltransferase CheR	0.144	−2.7928	4.98E-08	1.47E-07	yes	down	mRNA
VIBR0546_03115	VIBR0546_03115	hypothetical protein	0.177	−2.50016	5.2E-08	1.53E-07	yes	down	mRNA
VIBR0546_10129	VIBR0546_10129	hypothetical protein	0.228	−2.13169	5.27E-08	1.55E-07	yes	down	mRNA
VIBR0546_09979	VIBR0546_09979	putative transmembrane protein	0.234	−2.09237	5.44E-08	1.6E-07	yes	down	mRNA
VIBR0546_10784	VIBR0546_10784	dsDNA-mimic protein	0.218	−2.19705	6.18E-08	1.81E-07	yes	down	mRNA
VIBR0546_11662	VIBR0546_11662	regulatory protein MerR	0.123	−3.01827	6.34E-08	1.85E-07	yes	down	mRNA
VIBR0546_21505	VIBR0546_21505	3-hydroxyisobutyrate dehydrogenase	0.23	−2.12078	8.14E-08	2.35E-07	yes	down	mRNA
VIBR0546_08580	VIBR0546_08580	hypothetical protein	0.123	−3.02068	8.15E-08	2.35E-07	yes	down	mRNA
VIBR0546_00615	VIBR0546_00615	hypothetical protein	4.805	2.264394	8.42E-08	2.43E-07	yes	up	mRNA
VIBR0546_09579	VIBR0546_09579	putative nitrogen regulatory protein P-II family protein	0.249	−2.00684	8.95E-08	2.57E-07	yes	down	mRNA
VIBR0546_01681	VIBR0546_01681	acetyltransferase	0.206	−2.28172	9.26E-08	2.65E-07	yes	down	mRNA
VIBR0546_17133	VIBR0546_17133	hypothetical protein	0.001	−9.67856	1E-07	2.85E-07	yes	down	mRNA
VIBR0546_03570	VIBR0546_03570	hypothetical protein	4.035	2.01264	1.1E-07	3.13E-07	yes	up	mRNA
VIBR0546_14897	VIBR0546_14897	arginine N-succinyltransferase	4.186	2.065506	1.11E-07	3.16E-07	yes	up	mRNA
VIBR0546_18897	fliR	flagellar biosynthesis protein FliR	4.758	2.250322	1.15E-07	3.27E-07	yes	up	mRNA
VIBR0546_12192	VIBR0546_12192	ketoglutarate semialdehyde dehydrogenase	0.214	−2.22581	1.17E-07	3.29E-07	yes	down	mRNA
VIBR0546_21485	VIBR0546_21485	methylmalonate-semialdehyde dehydrogenase	0.199	−2.3311	1.19E-07	3.36E-07	yes	down	mRNA
VIBR0546_03845	VIBR0546_03845	putative response regulator	0.129	−2.95014	1.22E-07	3.44E-07	yes	down	mRNA
VIBR0546_19072	VIBR0546_19072	elongation factor Tu	0.245	−2.02831	1.27E-07	3.56E-07	yes	down	mRNA
VIBR0546_19469	VIBR0546_19469	hypothetical protein	4.544	2.183993	1.37E-07	3.84E-07	yes	up	mRNA
VIBR0546_20575	VIBR0546_20575	acetyltransferase-like protein	4.271	2.094658	1.44E-07	4.02E-07	yes	up	mRNA
VIBR0546_13012	VIBR0546_13012	putative metal-dependent hydrolase	4.895	2.291232	1.67E-07	4.65E-07	yes	up	mRNA
VIBR0546_20690	VIBR0546_20690	transcriptional regulatory protein%2C C	0.067	−3.90784	1.68E-07	4.69E-07	yes	down	mRNA
VIBR0546_06597	VIBR0546_06597	hypothetical protein	7.529	2.912433	1.81E-07	5.03E-07	yes	up	mRNA
VIBR0546_11372	VIBR0546_11372	Allophanate hydrolase subunit 1	0.215	−2.21574	2.12E-07	5.87E-07	yes	down	mRNA
VIBR0546_12302	VIBR0546_12302	Protein pecM	0.22	−2.18699	2.16E-07	5.97E-07	yes	down	mRNA
VIBR0546_17188	VIBR0546_17188	phosphonate ABC transporter%2C permease protein PhnE	0.15	−2.73849	2.26E-07	6.24E-07	yes	down	mRNA
VIBR0546_13705	VIBR0546_13705	electron transfer flavoprotein subunit beta	0.163	−2.61532	2.66E-07	7.3E-07	yes	down	mRNA

VIBR0546_19362	VIBR0546_19362	DNA polymerase III subunit chi	5.305	2.407325	2.72E-07	7.44E-07	yes	up	mRNA
VIBR0546_18286	queA	S-adenosylmethionine:tRNA ribosyltransferase-isomerase	5.273	2.398627	2.93E-07	8E-07	yes	up	mRNA
VIBR0546_01466	VIBR0546_01466	hypothetical protein	0.25	−2.00052	2.97E-07	8.09E-07	yes	down	mRNA
VIBR0546_13690	VIBR0546_13690	branched-chain alpha-keto acid dehydrogenase E1 component beta subunit	0.211	−2.24421	3.1E-07	8.42E-07	yes	down	mRNA
VIBR0546_10589	VIBR0546_10589	universal stress protein A	0.245	−2.02632	3.43E-07	9.28E-07	yes	down	mRNA
VIBR0546_03525	VIBR0546_03525	Protoheme IX farnesyltransferase	0.196	−2.34836	3.64E-07	9.86E-07	yes	down	mRNA
VIBR0546_09217	VIBR0546_09217	hypothetical protein	0.193	−2.37528	3.83E-07	1.03E-06	yes	down	mRNA
VIBR0546_18942	VIBR0546_18942	hypothetical protein	0.056	−4.14697	3.99E-07	1.08E-06	yes	down	mRNA
VIBR0546_21510	fabG	3-ketoacyl-(acyl-carrier-protein) reductase	0.226	−2.14335	4.82E-07	1.29E-06	yes	down	mRNA
VIBR0546_11577	VIBR0546_11577	hypothetical protein	0.199	−2.32999	5.17E-07	1.38E-06	yes	down	mRNA
VIBR0546_12152	VIBR0546_12152	hypothetical protein	0.201	−2.31825	5.23E-07	1.39E-06	yes	down	mRNA
VIBR0546_11712	VIBR0546_11712	hypothetical protein	0.243	−2.03957	5.28E-07	1.41E-06	yes	down	mRNA
VIBR0546_19302	VIBR0546_19302	hypothetical protein	0.127	−2.97448	5.74E-07	1.52E-06	yes	down	mRNA
VIBR0546_16006	VIBR0546_16006	cell division protein FtsK	4.64	2.214024	5.9E-07	1.56E-06	yes	up	mRNA
VIBR0546_03305	VIBR0546_03305	glutathione S-transferase	0.22	−2.1835	6.14E-07	1.62E-06	yes	down	mRNA
VIBR0546_14555	VIBR0546_14555	putative deoxycytidylate deaminase	0.19	−2.39424	6.97E-07	1.83E-06	yes	down	mRNA
VIBR0546_10014	VIBR0546_10014	response regulator	0.139	−2.84348	7.57E-07	1.98E-06	yes	down	mRNA
VIBR0546_06407	VIBR0546_06407	LysM domain protein	0.117	−3.09774	7.65E-07	2E-06	yes	down	mRNA
VIBR0546_01666	VIBR0546_01666	AraC family transcriptional regulator	0.223	−2.16806	8.37E-07	2.19E-06	yes	down	mRNA
VIBR0546_19147	VIBR0546_19147	hypothetical protein	4.143	2.050651	8.64E-07	2.25E-06	yes	up	mRNA
VIBR0546_08550	VIBR0546_08550	hypothetical protein	9.364	3.227159	8.66E-07	2.26E-06	yes	up	mRNA
VIBR0546_06672	VIBR0546_06672	hypothetical protein	6.314	2.65865	8.77E-07	2.28E-06	yes	up	mRNA
VIBR0546_08757	VIBR0546_08757	fumarate reductase flavoprotein subunit	4.274	2.095548	8.81E-07	2.29E-06	yes	up	mRNA
VIBR0546_18952	VIBR0546_18952	hypothetical protein	5.902	2.56112	8.99E-07	2.33E-06	yes	up	mRNA
VIBR0546_03760	VIBR0546_03760	Permease of the drug/metabolite transporter (DMT) superfamily protein	4.659	2.219904	9.09E-07	2.36E-06	yes	up	mRNA
VIBR0546_14540	codB	cytosine permease	0.239	−2.06202	9.25E-07	2.4E-06	yes	down	mRNA
VIBR0546_03775	VIBR0546_03775	glycine dehydrogenase	0.188	−2.41247	9.29E-07	2.4E-06	yes	down	mRNA
VIBR0546_20113	VIBR0546_20113	phosphonate metabolism transcriptional regulator PhnF	4.47	2.160348	9.29E-07	2.4E-06	yes	up	mRNA
VIBR0546_17768	VIBR0546_17768	deaminase-reductase domain-containing protein	0.202	−2.30571	9.6E-07	2.48E-06	yes	down	mRNA
VIBR0546_06327	VIBR0546_06327	hypothetical protein	4.771	2.254412	9.9E-07	2.55E-06	yes	up	mRNA
VIBR0546_09459	VIBR0546_09459	hypothetical protein	0.24	−2.0607	1.01E-06	2.61E-06	yes	down	mRNA
VIBR0546_13695	VIBR0546_13695	branched-chain alpha-keto acid dehydrogenase subunit E2	0.2	−2.32421	1.07E-06	2.75E-06	yes	down	mRNA
VIBR0546_11168	VIBR0546_11168	hypothetical protein	9.298	3.216951	1.13E-06	2.89E-06	yes	up	mRNA
VIBR0546_15311	VIBR0546_15311	ribosomal-protein-alanine acetyltransferase	4.24	2.084091	1.19E-06	3.04E-06	yes	up	mRNA
VIBR0546_12032	VIBR0546_12032	LysR family transcriptional regulator	0.236	−2.0851	1.21E-06	3.08E-06	yes	down	mRNA
VIBR0546_13555	VIBR0546_13555	response regulator	7.221	2.852295	1.34E-06	3.39E-06	yes	up	mRNA

VIBR0546_02760	VIBR0546_02760	glyoxalase/bleomycin resistance protein/dioxygenase	0.158	−2.66076	1.38E-06	3.48E-06	yes	down	mRNA
VIBR0546_01114	VIBR0546_01114	phosphocarrier protein NPr	4.953	2.308327	1.38E-06	3.48E-06	yes	up	mRNA
VIBR0546_13915	VIBR0546_13915	hypothetical protein	0.208	−2.26675	1.38E-06	3.5E-06	yes	down	mRNA
VIBR0546_01251	dnaK	molecular chaperone DnaK	0.193	−2.37446	1.54E-06	3.88E-06	yes	down	mRNA
VIBR0546_09499	VIBR0546_09499	hypothetical protein	5.618	2.490044	1.56E-06	3.93E-06	yes	up	mRNA
VIBR0546_21195	VIBR0546_21195	putative manganese transporter 11 TMS	4.717	2.237983	1.56E-06	3.93E-06	yes	up	mRNA
VIBR0546_17753	VIBR0546_17753	hypothetical protein	0.151	−2.73111	1.58E-06	3.96E-06	yes	down	mRNA
VIBR0546_05723	VIBR0546_05723	hypothetical protein	0.043	−4.55303	1.67E-06	4.18E-06	yes	down	mRNA
VIBR0546_11802	VIBR0546_11802	hypothetical protein	0.243	−2.04265	2.18E-06	5.4E-06	yes	down	mRNA
VIBR0546_12217	VIBR0546_12217	dihydrodipicolinate synthase	0.17	−2.55829	2.22E-06	5.51E-06	yes	down	mRNA
VIBR0546_19192	VIBR0546_19192	PTS system nitrogen regulatory IIA component	4.505	2.171645	2.35E-06	5.81E-06	yes	up	mRNA
VIBR0546_10214	VIBR0546_10214	hypothetical protein	0.179	−2.47893	2.46E-06	6.05E-06	yes	down	mRNA
VIBR0546_13720	VIBR0546_13720	hypothetical protein	4.695	2.231206	2.52E-06	6.21E-06	yes	up	mRNA
VIBR0546_20463	VIBR0546_20463	hypothetical protein	6.828	2.771471	2.69E-06	6.59E-06	yes	up	mRNA
VIBR0546_18421	VIBR0546_18421	hypothetical protein	4.518	2.175619	3.02E-06	7.36E-06	yes	up	mRNA
VIBR0546_11992	VIBR0546_11992	hypothetical protein	0.003	−8.41905	3.5E-06	8.48E-06	yes	down	mRNA
VIBR0546_14175	VIBR0546_14175	anti-sigma regulatory factor	0.238	−2.07073	4.08E-06	9.83E-06	yes	down	mRNA
VIBR0546_18406	VIBR0546_18406	copper homeostasis protein cutC	0.141	−2.83103	4.11E-06	9.9E-06	yes	down	mRNA
VIBR0546_03215	VIBR0546_03215	hypothetical protein	0.165	−2.5958	4.19E-06	1.01E-05	yes	down	mRNA
VIBR0546_10414	VIBR0546_10414	hypothetical protein	0.092	−3.44089	4.39E-06	1.05E-05	yes	down	mRNA
VIBR0546_21600	VIBR0546_21600	putative sodium-type flagellar protein MotY precursor	6.971	2.801438	5.54E-06	1.31E-05	yes	up	mRNA
VIBR0546_18932	VIBR0546_18932	hypothetical protein	0.114	−3.1299	5.63E-06	1.33E-05	yes	down	mRNA
VIBR0546_04087	VIBR0546_04087	hypothetical protein	0.249	−2.00562	5.7E-06	1.35E-05	yes	down	mRNA
VIBR0546_17688	VIBR0546_17688	adenylate kinase	0.156	−2.67813	6.01E-06	1.42E-05	yes	down	mRNA
VIBR0546_17563	VIBR0546_17563	hypothetical protein	0.124	−3.00606	6.51E-06	1.53E-05	yes	down	mRNA
VIBR0546_10224	VIBR0546_10224	hypothetical protein	0.237	−2.07515	6.57E-06	1.55E-05	yes	down	mRNA
VIBR0546_06267	VIBR0546_06267	oxidoreductase	0.151	−2.7279	7.07E-06	1.66E-05	yes	down	mRNA
VIBR0546_21470	VIBR0546_21470	isovaleryl-CoA dehydrogenase	0.242	−2.04687	7.35E-06	1.72E-05	yes	down	mRNA
VIBR0546_05733	VIBR0546_05733	hypothetical protein	0.187	−2.42206	7.55E-06	1.76E-05	yes	down	mRNA
VIBR0546_10924	VIBR0546_10924	membrane-bound lytic murein transglycosylase D	8.3	3.053168	8.94E-06	2.07E-05	yes	up	mRNA
VIBR0546_08732	VIBR0546_08732	ATP-dependent protease subunit HslV	0.131	−2.93772	9.31E-06	2.15E-05	yes	down	mRNA
VIBR0546_05982	VIBR0546_05982	MutT/NUDIX protein	0.186	−2.42776	9.41E-06	2.17E-05	yes	down	mRNA
VIBR0546_14862	VIBR0546_14862	hypothetical protein	5.269	2.397443	1.25E-05	2.85E-05	yes	up	mRNA
VIBR0546_03510	VIBR0546_03510	hypothetical protein	0.244	−2.03229	1.25E-05	2.86E-05	yes	down	mRNA
VIBR0546_13950	VIBR0546_13950	transcriptional regulator	0.062	−4.00006	1.34E-05	3.04E-05	yes	down	mRNA
VIBR0546_17383	VIBR0546_17383	ferredoxin	0.136	−2.88137	1.45E-05	3.28E-05	yes	down	mRNA
VIBR0546_01641	VIBR0546_01641	1-phosphofructokinase	0.24	−2.0595	1.45E-05	3.28E-05	yes	down	mRNA
VIBR0546_09424	VIBR0546_09424	hypothetical protein	4.572	2.192968	1.48E-05	3.34E-05	yes	up	mRNA

VIBR0546_00135	VIBR0546_00135	hypothetical protein	7.066	2.820831	1.49E-05	3.36E-05	yes	up	mRNA
VIBR0546_14525	VIBR0546_14525	permease	0.169	−2.56604	1.58E-05	3.54E-05	yes	down	mRNA
VIBR0546_17483	VIBR0546_17483	group 1 glycosyl transferase	0.244	−2.03638	1.63E-05	3.64E-05	yes	down	mRNA
VIBR0546_13840	VIBR0546_13840	hypothetical protein	0.101	−3.30704	1.7E-05	3.8E-05	yes	down	mRNA
VIBR0546_21900	VIBR0546_21900	NAD/NADP octopine/nopalinedehydrogenase	5.886	2.557184	1.72E-05	3.83E-05	yes	up	mRNA
VIBR0546_07727	VIBR0546_07727	hypothetical protein	0.141	−2.82452	1.78E-05	3.97E-05	yes	down	mRNA
VIBR0546_03485	VIBR0546_03485	cytochrome c oxidase%2C subunit II	0.249	−2.00858	1.78E-05	3.97E-05	yes	down	mRNA
VIBR0546_11812	VIBR0546_11812	putative glutamine amidotransferase	0.23	−2.1203	1.8E-05	4E-05	yes	down	mRNA
VIBR0546_19047	VIBR0546_19047	hypothetical protein	6.133	2.616601	1.87E-05	4.16E-05	yes	up	mRNA
VIBR0546_01421	VIBR0546_01421	hypothetical protein	4.556	2.187875	2.2E-05	4.83E-05	yes	up	mRNA
VIBR0546_01876	VIBR0546_01876	pilus assembly protein CpaB	7.762	2.956336	2.23E-05	4.9E-05	yes	up	mRNA
VIBR0546_16678	VIBR0546_16678	type IV pilus assembly protein PilN	4.861	2.281198	2.31E-05	5.06E-05	yes	up	mRNA
VIBR0546_09147	VIBR0546_09147	hypothetical protein	4.705	2.234211	2.35E-05	5.14E-05	yes	up	mRNA
VIBR0546_06587	VIBR0546_06587	hypothetical protein	16.631	4.055819	2.59E-05	5.65E-05	yes	up	mRNA
VIBR0546_07267	VIBR0546_07267	tonB1 protein	8.178	3.031663	2.61E-05	5.69E-05	yes	up	mRNA
VIBR0546_17478	VIBR0546_17478	glycosyl transferase group 1	0.177	−2.50086	3.55E-05	7.62E-05	yes	down	mRNA
VIBR0546_01236	VIBR0546_01236	heat shock protein GrpE	0.158	−2.65999	3.98E-05	8.49E-05	yes	down	mRNA
VIBR0546_13685	VIBR0546_13685	putative pyruvate dehydrogenase E1 component%2C alpha subunit	0.242	−2.04472	4.38E-05	9.3E-05	yes	down	mRNA
VIBR0546_03495	VIBR0546_03495	putative cytochrome c oxidase assembly transmembrane protein	0.176	−2.50667	4.41E-05	9.35E-05	yes	down	mRNA
VIBR0546_21075	VIBR0546_21075	heat shock protein HtpX	0.226	−2.14637	4.52E-05	9.56E-05	yes	down	mRNA
VIBR0546_06137	VIBR0546_06137	dihydrodipicolinate synthase	0.159	−2.65468	4.67E-05	9.87E-05	yes	down	mRNA
VIBR0546_21475	VIBR0546_21475	putative transcriptional regulator	0.212	−2.23945	4.97E-05	0.000105	yes	down	mRNA
VIBR0546_21280	VIBR0546_21280	lipase chaperone	0.233	−2.09957	5.04E-05	0.000106	yes	down	mRNA
VIBR0546_12312	VIBR0546_12312	acetyltransferase	0.229	−2.12435	5.22E-05	0.000109	yes	down	mRNA
VIBR0546_00605	VIBR0546_00605	hypothetical protein	10.153	3.343831	7.6E-05	0.000156	yes	up	mRNA
VIBR0546_10229	VIBR0546_10229	hypothetical protein	0.194	−2.36666	7.8E-05	0.00016	yes	down	mRNA
VIBR0546_13775	VIBR0546_13775	hypothetical protein	0.245	−2.02973	8.82E-05	0.00018	yes	down	mRNA
VIBR0546_18601	VIBR0546_18601	hypothetical protein	6.515	2.703657	9.02E-05	0.000184	yes	up	mRNA
VIBR0546_00790	VIBR0546_00790	hypothetical protein	5.087	2.346827	9.35E-05	0.00019	yes	up	mRNA
VIBR0546_11562	VIBR0546_11562	hypothetical protein	0.124	−3.00958	9.73E-05	0.000198	yes	down	mRNA
VIBR0546_00894	VIBR0546_00894	NptA protein	0.231	−2.11632	0.000103	0.000208	yes	down	mRNA
VIBR0546_12747	VIBR0546_12747	hypothetical protein	5.027	2.329833	0.000111	0.000223	yes	up	mRNA
VIBR0546_10124	VIBR0546_10124	hypothetical protein	0.225	−2.15244	0.000112	0.000225	yes	down	mRNA
VIBR0546_14440	VIBR0546_14440	MoxR-like ATPase	0.228	−2.135	0.000116	0.000233	yes	down	mRNA
VIBR0546_06412	VIBR0546_06412	hypothetical protein	0.185	−2.43412	0.000118	0.000238	yes	down	mRNA
VIBR0546_17078	VIBR0546_17078	hypothetical protein	0.218	−2.19551	0.00012	0.000241	yes	down	mRNA

VIBR0546_19262	VIBR0546_19262	RNase E inhibitor protein	0.199	−2.32701	0.000121	0.000242	yes	down	mRNA
VIBR0546_20328	VIBR0546_20328	putative outer membrane adhesin like protein	4.369	2.127269	0.000125	0.00025	yes	up	mRNA
VIBR0546_10659	thiE	thiamine-phosphate pyrophosphorylase	4.006	2.002209	0.000131	0.000262	yes	up	mRNA
VIBR0546_04217	VIBR0546_04217	Multidrug resistance protein B	4.256	2.089498	0.000133	0.000264	yes	up	mRNA
VIBR0546_09152	VIBR0546_09152	hypothetical protein	4.361	2.124673	0.000161	0.000317	yes	up	mRNA
VIBR0546_06612	VIBR0546_06612	ABC-2 type transporter	4.012	2.004388	0.000162	0.000319	yes	up	mRNA
VIBR0546_17558	VIBR0546_17558	transcriptional regulator	0.191	−2.38979	0.000175	0.000343	yes	down	mRNA
VIBR0546_18476	VIBR0546_18476	hypothetical protein	4.336	2.116419	0.000239	0.000461	yes	up	mRNA
VIBR0546_21835	VIBR0546_21835	hypothetical protein	0.145	−2.79054	0.000241	0.000464	yes	down	mRNA
VIBR0546_15911	VIBR0546_15911	hypothetical protein	18.225	4.187858	0.000254	0.000489	yes	up	mRNA
VIBR0546_20248	VIBR0546_20248	hypothetical protein	5.214	2.382505	0.000266	0.000509	yes	up	mRNA
VIBR0546_06737	VIBR0546_06737	putative TRAP-transporter extracellular solute-binding protein	0.055	−4.1943	0.000281	0.000537	yes	down	mRNA
VIBR0546_03500	VIBR0546_03500	cytochrome c oxidase%2C subunit III	0.204	−2.29647	0.000282	0.000537	yes	down	mRNA
VIBR0546_02549	VIBR0546_02549	hypothetical protein	4.533	2.180461	0.000303	0.000576	yes	up	mRNA
VIBR0546_00815	VIBR0546_00815	DSBA oxidoreductase	0.239	−2.06634	0.000338	0.000637	yes	down	mRNA
VIBR0546_14360	VIBR0546_14360	hypothetical protein	0.109	−3.19572	0.000345	0.00065	yes	down	mRNA
VIBR0546_17518	VIBR0546_17518	putative transthyretin family protein	0.188	−2.41321	0.000366	0.000686	yes	down	mRNA
VIBR0546_06187	VIBR0546_06187	hypothetical protein	0.164	−2.61154	0.000453	0.000837	yes	down	mRNA
VIBR0546_08309	cpxP	periplasmic repressor CpxP	0.245	−2.02864	0.000474	0.000873	yes	down	mRNA
VIBR0546_17708	VIBR0546_17708	hypothetical protein	0.163	−2.61547	0.000586	0.001068	yes	down	mRNA
VIBR0546_15611	VIBR0546_15611	hypothetical protein	0.244	−2.03398	0.000592	0.001078	yes	down	mRNA
VIBR0546_13062	VIBR0546_13062	ABC transporter transmembrane protein	6.335	2.663439	0.000612	0.001112	yes	up	mRNA
VIBR0546_21595	VIBR0546_21595	putative flagellar motor switch protein	7.828	2.968635	0.000775	0.001394	yes	up	mRNA
VIBR0546_09087	VIBR0546_09087	hypothetical protein	30.916	4.950304	0.000856	0.001532	yes	up	mRNA
VIBR0546_08385	VIBR0546_08385	hypothetical protein	26.39	4.721928	0.001155	0.002033	yes	up	mRNA
VIBR0546_07257	VIBR0546_07257	TonB system transport protein ExbD1	7.999	2.999836	0.001446	0.002519	yes	up	mRNA
VIBR0546_10914	VIBR0546_10914	LfgM protein	7.38	2.88356	0.001466	0.00255	yes	up	mRNA
VIBR0546_11492	VIBR0546_11492	hypothetical protein	0.154	−2.70354	0.001528	0.00265	yes	down	mRNA
VIBR0546_19042	VIBR0546_19042	hypothetical protein	23.338	4.544616	0.002022	0.003449	yes	up	mRNA
VIBR0546_01501	VIBR0546_01501	hypothetical protein	0.167	−2.5833	0.002117	0.0036	yes	down	mRNA
VIBR0546_01821	VIBR0546_01821	Outer membrane protein	0.217	−2.20235	0.002255	0.003821	yes	down	mRNA
VIBR0546_01866	VIBR0546_01866	hypothetical protein	8.345	3.060863	0.002909	0.004834	yes	up	mRNA
VIBR0546_06207	VIBR0546_06207	metal dependent phosphohydrolase	6.759	2.756861	0.003404	0.005596	yes	up	mRNA
VIBR0546_17608	ureB	urease subunit beta	0.153	−2.71028	0.003869	0.006322	yes	down	mRNA
VIBR0546_09849	VIBR0546_09849	hypothetical protein	0.137	−2.86379	0.005177	0.008308	yes	down	mRNA
VIBR0546_06572	VIBR0546_06572	FMN-binding domain-containing protein	4.064	2.023018	0.00571	0.009102	yes	up	mRNA
VIBR0546_10434	VIBR0546_10434	hypothetical protein	0.248	−2.01323	0.006056	0.009625	yes	down	mRNA

VIBR0546_03635	VIBR0546_03635	TRAP dicarboxylate family transporter%2C DctQ subunit	0.093	−3.43102	0.006309	0.009995	yes	down	mRNA
VIBR0546_01846	VIBR0546_01846	putative Flp pilus assembly protein TadC	4.042	2.015025	0.0069	0.010887	yes	up	mRNA
VIBR0546_06262	VIBR0546_06262	acetyltransferase	0.214	−2.22119	0.00813	0.012655	yes	down	mRNA
VIBR0546_07482	VIBR0546_07482	Fe2+ transport system protein A	0.249	−2.00429	0.009423	0.014512	yes	down	mRNA
VIBR0546_01261	VIBR0546_01261	putative fimbrial assembly protein PilE	4.762	2.25154	0.010769	0.016496	yes	up	mRNA
VIBR0546_10349	VIBR0546_10349	hypothetical protein	0.032	−4.97872	0.01124	0.017151	yes	down	mRNA
VIBR0546_09439	VIBR0546_09439	hypothetical protein	4.008	2.00299	0.011773	0.017901	yes	up	mRNA
VIBR0546_14275	VIBR0546_14275	hypothetical protein	4.796	2.261816	0.01698	0.025213	yes	up	mRNA
VIBR0546_07939	VIBR0546_07939	putative N-acetylglucosamine kinase	5.812	2.53896	0.01739	0.025751	yes	up	mRNA
VIBR0546_06167	VIBR0546_06167	hypothetical protein	0.157	−2.67226	0.023728	0.03453	yes	down	mRNA
VIBR0546_10919	VIBR0546_10919	hypothetical protein	5.535	2.468692	0.024247	0.035251	yes	up	mRNA
VIBR0546_21585	fliP	flagellar biosynthesis protein FliP	4.34	2.117579	0.027723	0.039926	yes	up	mRNA
VIBR0546_13580	VIBR0546_13580	nitrate ABC transporter permease protein	0.241	−2.05546	0.030806	0.043898	yes	down	mRNA

Note: where the E stands for the times 10 to the power, for example, 1.8E-12 = 1.8×10^{-12}.

Table S4. Differentially expressed sigma factors of *V. brasiliensis* cultured with different NaCl concentrations in transcriptome analysis.

Gene_id	Gene name	Gene description	FC(Nacl_0/CK)	Log2FC(Nacl_0/CK)	*p* Value	*p* Adjust	Significant	Regulate	Type	
VIBR0546_10019	VIBR0546_10019	putative anti-sigma F factor antagonist	0.085	−3.5619292	7.784E-27	8.3936E-25	yes	down	mRNA	NaCl_0
VIBR0546_00505	VIBR0546_00505	putative anti-sigma regulatory factor	0.053	−4.2255434	1.663E-23	1.3039E-21	yes	down	mRNA	
VIBR0546_05309	VIBR0546_05309	RNA polymerase sigma factor RpoS	0.183	−2.4473742	2.986E-18	1.4471E-16	yes	down	mRNA	
VIBR0546_05608	VIBR0546_05608	RNA polymerase sigma factor	4.189	2.0666119	4.556E-16	1.8363E-14	yes	up	mRNA	
VIBR0546_04769	VIBR0546_04769	sigma factor RpoE regulatory protein RseC	20.889	4.3846912	5.168E-60	3.9796E-58	yes	up	mRNA	NaCl_7
VIBR0546_04774	rseB	anti-sigma E factor	8.397	3.0698963	1.373E-45	5.6371E-44	yes	up	mRNA	
VIBR0546_05309	VIBR0546_05309	RNA polymerase sigma factor RpoS	0.028	−5.162849	7.736E-90	2.2238E-87	yes	down	mRNA	
VIBR0546_00505	VIBR0546_00505	putative anti-sigma regulatory factor	0.03	−5.065646	5.078E-36	1.2164E-34	yes	down	mRNA	
VIBR0546_19674	VIBR0546_19674	putative sigma-54 modulation protein	0.17	−2.557331	5.811E-22	5.3888E-21	yes	down	mRNA	
VIBR0546_02631	VIBR0546_02631	anti-RNA polymerase sigma 70 factor	0.226	−2.1450219	1.731E-21	1.5584E-20	yes	down	mRNA	
VIBR0546_14185	VIBR0546_14185	anti-anti-sigma regulatory factor	0.103	−3.2736327	2.615E-18	1.7872E-17	yes	down	mRNA	
VIBR0546_10019	VIBR0546_10019	putative anti-sigma F factor antagonist	0.091	−3.459751	3.04E-09	1.0084E-08	yes	down	mRNA	
VIBR0546_14180	VIBR0546_14180	anti-anti-sigma regulatory factor	0.158	−2.6639467	7.125E-09	2.2875E-08	yes	down	mRNA	
VIBR0546_14175	VIBR0546_14175	anti-sigma regulatory factor	0.238	−2.0707281	4.078E-06	9.8345E-06	yes	down	mRNA	

Note: where the E stands for the times 10 to the power, for example, 1.8E-12 = 1.8×10^{-1}
